# Polymeric alpha-1 antitrypsin perturbs Paneth cell proteostasis and exacerbates intestinal inflammation

**DOI:** 10.1038/s41392-026-02925-9

**Published:** 2026-09-29

**Authors:** Francesco Annunziata, Felipe Dos Santos Matos, Irene Relvini, Jing Lu, Giovanna Federico, Claudia D’Agostino, Valentina Schiano, Veronica Maffia, Bruno Maria Custode, Gaetano Raiola, Barbara De Marino, Rossella De Cegli, Eugenio Del Prete, Elena Polishchuk, Carmen Ambrosio, Debora Compare, Gerardo Nardone, Francesco Neri, Nunzia Pastore

**Affiliations:** 1 Telethon Institute of Genetics and Medicine (TIGEM), Pozzuoli, Italy; 2 Scuola Superiore Meridionale (SSM), Genomics and Experimental Medicine Program, Naples, Italy; 3 Leibniz Institute on Aging, Fritz Lipmann Institute (FLI), Jena, Germany; 4 Gastroenterology Unit, Department of Clinical Medicine and Surgery, Federico II University, Naples, Italy; 5 Department of Life Sciences and Systems Biology and Molecular Biotechnology Center, University of Turin, Torino, Italy; 6 Department of Translational Medicine, Medical Genetics, Federico II University, Naples, Italy

**Keywords:** Medical genetics, Gastrointestinal diseases

## Abstract

Alpha-1 antitrypsin (AAT) is a serine protease inhibitor that protects tissue from neutrophil elastase and other proteases, particularly in the lung. Mutations in *SERPINA1*, including the Z mutation, lead to AAT deficiency (AATD), characterized by reduced circulating AAT and increased risk of pulmonary emphysema, liver cirrhosis, and hepatocellular carcinoma. Beyond these well-characterized manifestations, AATD has been associated with panniculitis, rheumatoid arthritis, and glomerulonephritis. Emerging evidence has also suggested a link between AATD and inflammatory bowel diseases (IBDs), although experimental validation is lacking. In this study, we demonstrate that PiZ transgenic mice expressing the polymer-forming ATZ display increased susceptibility to dextran sodium sulfate (DSS)-induced colitis, accompanied by marked Paneth cell abnormalities. The accumulation of polymeric ATZ in Paneth cells is associated with the endoplasmic reticulum (ER) stress response, impaired lysosomal clearance, altered association of Lysozyme-1 (Lyz1) with LC3-containing compartments, and increased Lyz1 secretion. These intestinal alterations were accompanied by changes in microbiota composition, whereas DSS exposure and exogenous lysozyme administration were associated with aggravated intestinal and hepatic pathology. Pharmacological inhibition of ER stress restored crypt homeostasis and normalized Lyz1 secretion. Human Pi*ZZ iPSC-derived intestinal organoids similarly showed ATZ polymer accumulation in secretory epithelial cells and transcriptional alterations involving ER protein processing and epithelial homeostasis. In addition, polymeric ATZ was detected in ileal crypts from a single individual with AATD and intestinal disease. Together, our data reveal a Paneth cell-intrinsic ER stress mechanism linking ATZ accumulation to gut epithelial dysfunction, highlighting a previously underexplored role of the gut–liver axis in AATD.

## Introduction

Alpha-1 antitrypsin (AAT) deficiency (AATD) is a genetic disorder caused by mutations in the highly polymorphic *SERPINA1* gene.^1^ The most common and severe mutation, known as the Pi*Z allele, results from a single amino acid substitution (Glu342Lys) that destabilizes the native conformation of AAT, promotes protein misfolding and polymerization within the endoplasmic reticulum (ER) of hepatocytes, and markedly reduces its secretion into the circulation. The clinical consequences of AATD reflect both loss- and gain-of-function mechanisms. Reduced circulating AAT compromises protection against neutrophil elastase and other serine proteases, thereby increasing susceptibility to early-onset pulmonary emphysema, whereas intracellular accumulation of polymeric ATZ within hepatocytes causes proteotoxic stress and contributes to chronic liver disease, cirrhosis, and hepatocellular carcinoma.^2^

AAT is a plasma glycoprotein primarily synthesized and secreted by hepatocytes in response to inflammatory signals.^3^ However, there is growing evidence of extrahepatic AAT production in various cell types, including neutrophils, monocytes, macrophages, pancreatic islets, and renal and intestinal epithelial cells (IECs).^4^ Within the small intestine, AAT-positive IECs have been identified along the crypt‒villus axis of the jejunum and ileum.^4^ Studies in PiZ mice, a widely used mouse model for AATD-associated liver disease,^5,6^ have further confirmed the presence of AAT-expressing IECs, specifically Paneth cells (PCs) and absorptive enterocytes. Despite these findings, the functional significance of AAT expression in IECs and the impact of ATZ mutation remain poorly understood.

Inflammatory bowel diseases (IBDs), including Crohn’s disease (CD) and ulcerative colitis (UC), are chronic inflammatory disorders of the gastrointestinal tract characterized by symptoms such as weight loss, diarrhea, nausea, and abdominal pain.^7^ The etiology of IBDs is multifactorial and involves a complex interplay of genetic predisposition and environmental factors.^8^ Several studies suggest that AATD may contribute to the pathogenesis of IBDs. A Swedish study reported that 8.5% of patients with UC were MZ heterozygotes, a significantly higher percentage than the 4.7% observed in the general Swedish population.^9^ Additionally, a broader review of AATD revealed that 1.2% of patients with the Pi*ZZ genotype also had IBDs. A UK-based study reviewing 651 patients with severe AATD (Pi*ZZ or PiZ null genotypes) reported a 1.5% prevalence of IBDs, notably higher than the 0.4% expected in the general population.^10,11^ Moreover, a retrospective study at the Mayo Clinic demonstrated that 9 out of 10 patients with concurrent AATD and UC developed more aggressive disease, were refractory to medical therapy, and required rapid colectomy, suggesting a protective role of endogenous AAT in intestinal homeostasis.^12^ A more recent study also reported a 10.6% incidence rate in individuals with the Pi*ZZ genotype compared with 3.6% for controls.^13^ These findings support the hypothesis that AATD may contribute to IBD susceptibility, particularly UC. Nevertheless, these associations do not establish causality, and the mechanisms through which AATD might modify intestinal disease susceptibility remain uncertain. Existing models have largely focused on systemic protease‒antiprotease imbalance, paralleling the mechanism of AATD-associated emphysema and proposing that reduced AAT levels may enhance neutrophil-mediated tissue injury during intestinal inflammation. However, this model does not account for the possible effects of mutant ATZ accumulation within IECs themselves. In addition, AAT and AAT-derived peptides have been reported to influence inflammatory signaling, epithelial responses, and microbial interactions,^14,15^ further suggesting that the relationship between *SERPINA1* dysfunction and intestinal inflammation may extend beyond circulating antiprotease deficiency. Thus, it remains important to determine whether polymerogenic ATZ directly perturbs intestinal epithelial homeostasis and whether such alterations modify susceptibility to inflammatory injury.

The intestine and liver are connected through bidirectional metabolic, immune, and microbial signals collectively referred to as the gut–liver axis.^16^ Changes in epithelial integrity, antimicrobial peptide release, and microbiota composition can influence hepatic inflammation and fibrosis, while pre-existing liver disease can, in turn, modify intestinal physiology. This interaction may be particularly relevant in AATD, where the liver already experiences chronic proteotoxic stress due to ATZ accumulation. We therefore hypothesized that intestinal expression of polymerogenic ATZ creates an epithelial vulnerability that becomes evident under inflammatory challenge and may aggravate extraintestinal pathology.

In this study, we investigated whether intestinal expression of polymerogenic ATZ perturbs epithelial proteostasis and modifies susceptibility to inflammatory injury. We focused particularly on PCs because of their high secretory activity and their sensitivity to disturbances in ER homeostasis, vesicular trafficking, and autophagy-related degradation.^17^ Using complementary mouse, cellular, molecular, and human experimental systems, we examined the consequences of ATZ accumulation on intestinal epithelial architecture, stress responses, PC homeostasis, Lyz1 trafficking and secretion, microbiota composition, and the response to chemically induced colitis. We also explored whether intestinal alterations might influence the hepatic phenotype through the gut–liver axis. By addressing these questions, our study aimed to define the intestinal consequences of ATZ proteotoxicity and to determine whether AATD acts as a modifier of intestinal inflammatory susceptibility rather than as a direct cause of spontaneous disease.

Our findings identify PCs as a previously unrecognized target of ATZ-mediated proteotoxic stress and reveal a novel mechanism linking protein misfolding disorders to intestinal inflammation.

## Results

### AATD exacerbates susceptibility to DSS-induced acute colitis in mice

**Fig. 1:**
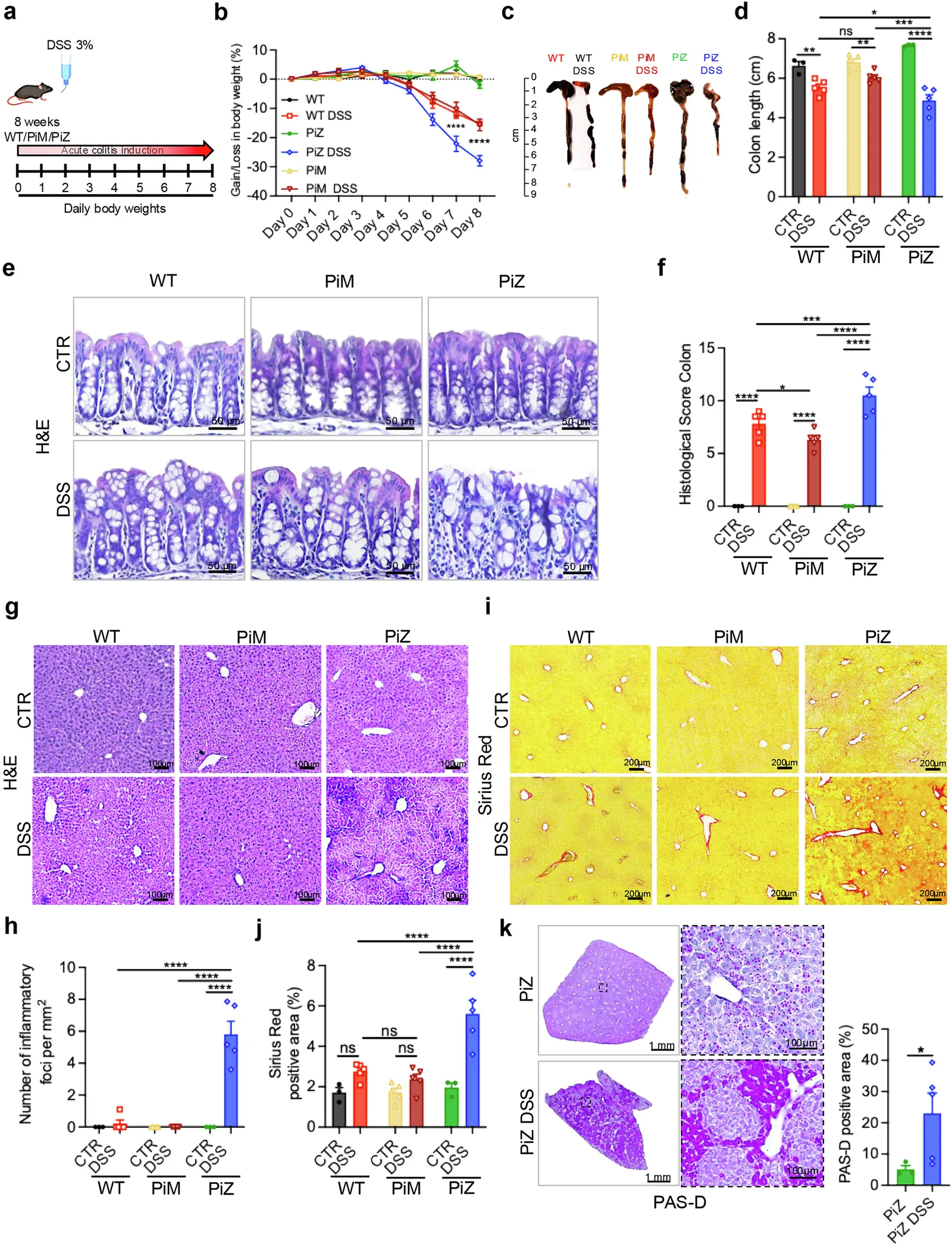
PiZ mice are more susceptible to DSS-induced colitis than control mice. **a** Schematic representation of the experimental design for the DSS experiment in 8-week-old WT, PiM, and PiZ mice. Mice received 3% DSS in their drinking water for 8 days. **b** Body weight changes in WT, PiM, and PiZ mice, with or without DSS treatment, over the course of the experiment. **c**, **d** Gross morphology of colons from mice of the indicated genotypes and quantification of colon length. **e**, **f** Representative images of H&E-stained colon sections from mice of the indicated genotypes and relative histopathological scores. **g**, **h** Representative images of H&E-stained liver sections from mice of the indicated genotypes and relative quantification of inflammatory foci. **i**, **j** Representative images of Sirius red-stained liver sections from mice of the indicated genotypes and relative quantification. **k** Representative images of PAS-D-stained liver sections from control and DSS-treated PiZ mice and relative quantification. Each dot represents an individual mouse. All the data refer to male mice. Data are presented as the mean ± standard error. Data normality was assessed using the Shapiro‒Wilk test. Statistical significance was determined using two-way ANOVA. For the data in (**h**), statistical significance was determined using an unpaired two-tailed *t*-test with Welch’s correction. ns not significant, *p < 0.05, **p < 0.01, ***p < 0.001, and ****p < 0.0001

To investigate whether the expression of polymerogenic ATZ alters susceptibility to intestinal inflammatory injury, we induced acute colitis in 8-week-old wild-type (WT), human normal AAT transgenic (PiM),^18^ and human Z mutant AAT transgenic (PiZ)^4^ mice by administering 3% dextran sodium sulfate (DSS) in the drinking water for 8 days (Fig. 1a). This well-established protocol is widely used to disrupt the intestinal epithelium and model colitis in mice.^19^ Body weight (BW) was monitored daily throughout the experiment. Compared with WT and PiM mice, PiZ mice exhibited significantly greater BW loss following DSS treatment (Fig. 1b). Macroscopic analysis revealed that compared with DSS-treated WT and PiM mice, DSS-treated PiZ mice had a marked reduction in colon length (Fig. 1c, d). Additionally, compared with untreated PiZ controls, DSS-treated PiZ mice displayed a shortened intestinal length, which was not observed in control mice (Supplementary Fig. 1a).

Histological analysis of the colon further confirmed a more severe colitis phenotype in the DSS-treated PiZ mice than in the DSS-treated WT and PiM mice, as indicated by a higher histopathology score (Fig. 1e, f). Interestingly, compared with control mice, PiM mice showed partial protection against intestinal injury, likely due to increased AAT secretion, which is consistent with previous reports.^20^ Notably, we observed an aberrant distribution of Lysozyme-positive cells (Lyz1^+^) along the intestinal villi in the ileum of DSS-treated PiZ mice, presumably ectopic PCs, which under physiological conditions are restricted to the base of intestinal crypts (Supplementary Fig. 1b).

In addition to intestinal pathology, compared with both control PiZ mice and DSS-treated WT and PiM mice, DSS-treated PiZ mice also exhibited exacerbated hepatic abnormalities. These mice showed increased hepatic inflammation and fibrosis, as demonstrated by a greater number of inflammatory foci (Fig. 1g, h), elevated mRNA levels of proinflammatory cytokines (*Tnfα* and *Il1β*) (Supplementary Fig. 1c), and increased collagen deposition (Fig. 1i, j and Supplementary Fig. 1d). Liver damage was further supported by increased serum ALT and AST levels in DSS-treated PiZ mice (Supplementary Fig. 1e, f). Furthermore, the accumulation of periodic acid-Schiff with diastase (PAS-D)-positive globules, a hallmark of AATD-associated liver disease, increased in DSS-treated PiZ mice (Fig. 1k).

Taken together, these findings indicate that PiZ mice are more susceptible to chemically induced colitis and suggest that intestinal inflammation can aggravate the pre-existing hepatic phenotype in this model.

### Mutant hATZ is expressed and forms polymers in IECs, altering intestinal morphology in mice

**Fig. 2:**
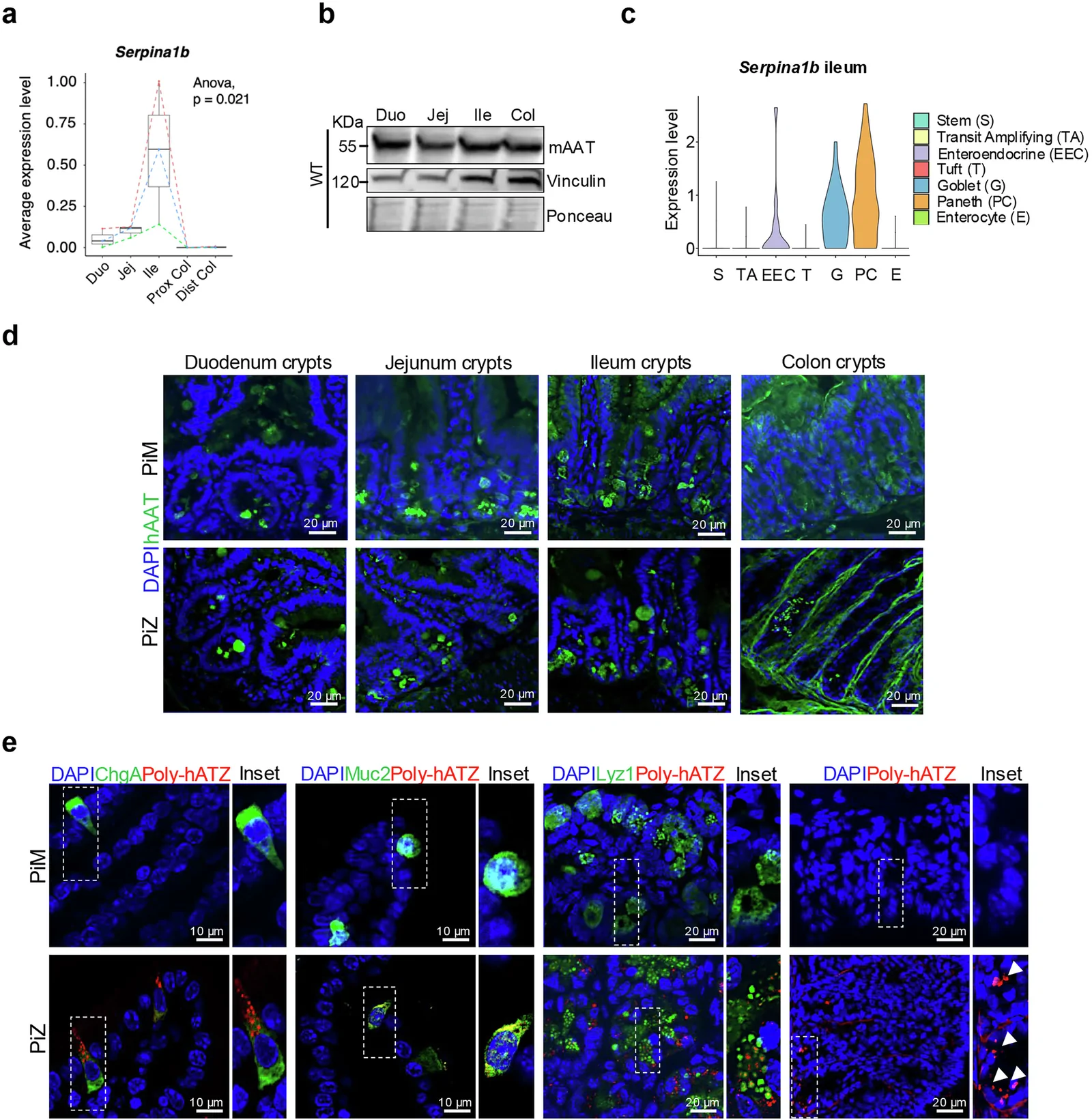
Mutant hATZ Forms Polymers in IECs and Disrupts Intestinal Architecture. **a** *Serpina1b* expression levels in the duodenum (Duo), jejunum (Jej), ileum (Ile), proximal colon (Prox Col), and distal colon (Dist Col) of WT mice. **b** Immunoblot analysis of murine AAT in different intestinal regions of WT mice. **c** Cell type-specific expression of *Serpina1b* across intestinal regions. Violin plots showing the distribution of *Serpina1b* expression levels across distinct epithelial cell types within the ileum based on single-cell RNA sequencing. **d** Representative immunofluorescence images showing hAAT accumulation in intestinal sections (duodenum, jejunum, ileum and colon) of PiM and PiZ mice. **e** Representative coimmunofluorescence images of ileal sections from PiM and PiZ mice stained for poly-hATZ and the cell-type markers ChgA, Muc2, and Lyz1

Previous studies have shown that AAT is expressed in the small intestine, specifically in secretory PCs and absorptive enterocytes.^4^ To further investigate the expression levels and distribution of AAT in the small intestine of adult WT mice, we analyzed previously published RNA-seq data from intestinal crypts isolated from different regions of the small intestine (duodenum, jejunum, and ileum) and two compartments of the large intestine (proximal and distal colon).^21^ Analysis of *Serpina1* isoforms in the intestine revealed that *Serpina1b* was the most highly expressed transcript, whereas the other five isoforms (*Serpina1a*, *c*, *d*, *e*, and *f*) were present at lower levels (Fig. 2a and Supplementary Fig. 2a). *Serpina1b* expression was predominantly localized to the small intestine, particularly in the ileum, with expression below the detection threshold in the analyzed RNA-seq dataset in the large intestine (Fig. 2a). In contrast to mRNA expression, AAT protein expression was detected in the colons of WT mice (Fig. 2b).

To further assess *Serpina1b* expression at the single-cell level, we used previously published single-cell RNA-seq data.^21^ We found that *Serpina1b* was broadly expressed across all IECs of the small intestine, with the highest levels observed in enteroendocrine cells (EECs), goblet cells, and PCs, all of which belong to the secretory lineage (Fig. 2c, Supplementary Fig. 2b, c).

Next, we evaluated the expression of the WT and the mutant human ATZ (hATZ) proteins in the small intestines of PiM and PiZ mice, respectively. Immunostaining and immunoblot analyses revealed extensive expression of hAAT along the crypt–villus axis of the small intestine and in the colonic epithelium in both PiM and PiZ mice (Fig. 2d and Supplementary Fig. 2d).

To determine whether hATZ forms polymers in IECs, as in hepatocytes, we performed coimmunostaining for cell type-specific markers alongside a polymer-specific hAAT antibody. This analysis revealed the presence of hATZ globules in EECs, goblet cells, and PCs in PiZ mice (Fig. 2e). Notably, we also detected hATZ globules in colon crypt cells, further confirming its expression in the colon (Fig. 2e).

Collectively, these findings confirm that hAAT is expressed in both small intestinal and colonic epithelial cells in mice and identify secretory cells as key sites of polymeric hATZ accumulation.

### Mutant hATZ expression disrupts intestinal architecture and induces ER stress-associated transcriptional responses

**Fig. 3:**
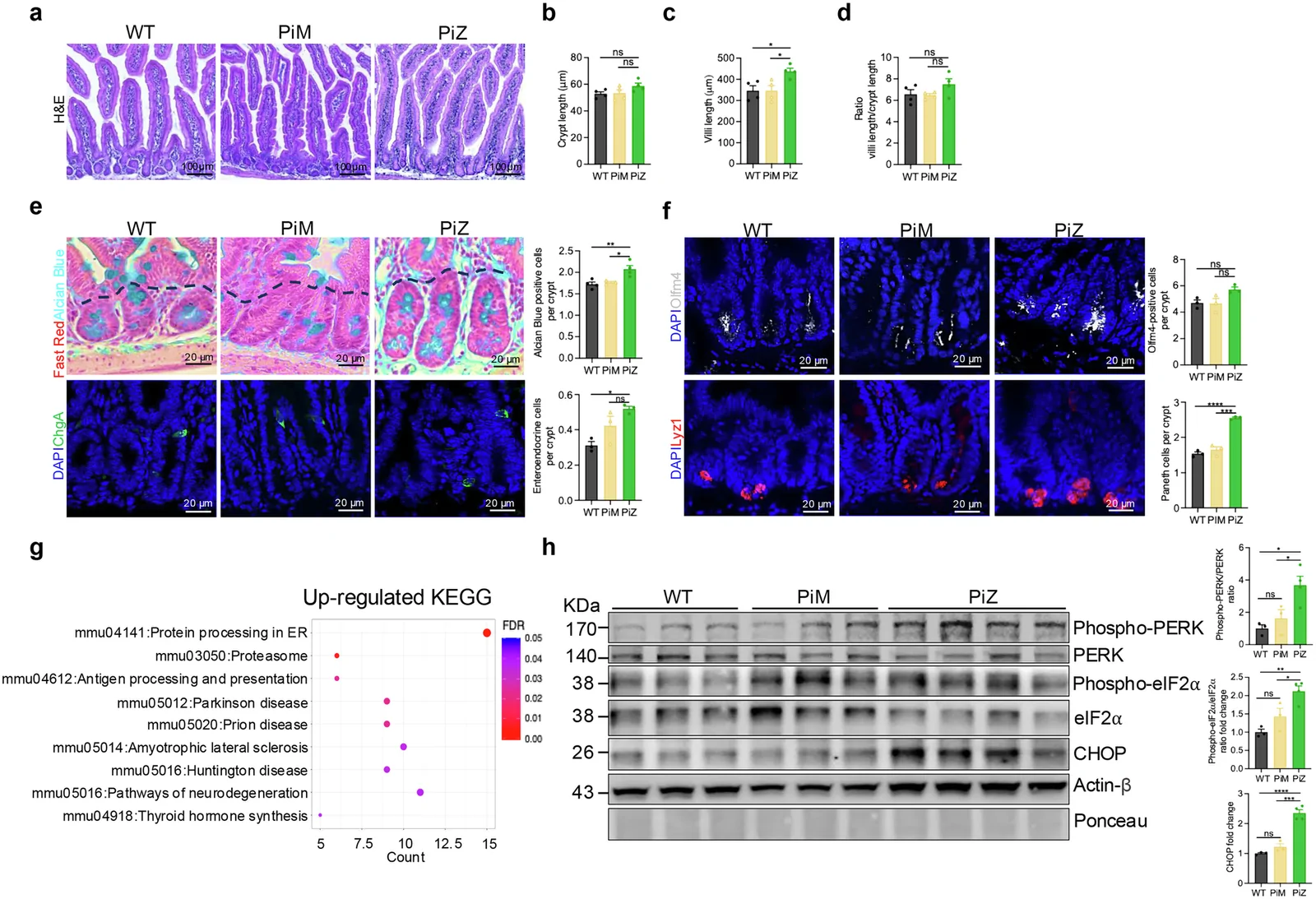
Intestinal epithelial remodeling and ER stress activation in PiZ mice expressing mutant hAAT. **a**–**d** Representative H&E images of duodenal sections from WT, PiM, and PiZ mice and quantification of the average crypt length, villus length, and villi-on-crypt ratio. **e**, **f** Representative images of duodenal sections stained for Alcian blue (goblet cells), ChgA (EECs), Lyz1 (PCs), and Olfm4 (stem cells) in WT, PiM and PiZ mice plus relative quantification. **g** KEGG pathway enrichment analysis of genes differentially expressed between intestinal crypts from PiZ and WT mice (FDR < 0.05). **h** Immunoblot analysis of UPR- and ER stress-related markers in WT, PiM, and PiZ livers under basal conditions with relative quantification. Each dot represents an individual mouse. All the data refer to male mice. Data are presented as the mean ± standard error. Data normality was assessed using the Shapiro‒Wilk test. Statistical significance was determined using one-way ANOVA. ns not significant, *p < 0.05, **p < 0.01, ***p < 0.001, and ****p < 0.0001

To assess the biological effects of mutant hATZ expression and accumulation in IECs, we conducted comparative morphological analyses of intestinal tissue from WT, PiM, and PiZ male mice. Histological analyses of the small intestine revealed a significant increase in villus length and a trend toward an increase in crypt length in PiZ mice compared with those in control mice (Fig. 3a–d). PiM mice showed no apparent differences compared with WT controls (Fig. 3a).

We next characterized the cellular composition of small intestinal crypts by evaluating the major cell types, including goblet cells, EECs, intestinal stem cells (ISCs), and PCs. Using Alcian blue staining, Chromogranin-A (ChgA), Olfactomedin-4 (Olfm4), and immunostaining for Lyz1, which are specific markers of goblet cells, EECs, ISCs, and PCs, respectively, we detected increases in the numbers of goblet and PCs in PiZ mice compared with those in WT and PiM mice (Fig. 3e, f).

Collectively, these findings indicate that mutant hATZ expression and accumulation disrupt small intestinal structure and alter crypt cellular composition.

To further explore the molecular consequences of mutant hATZ expression in the intestine, we performed a transcriptomic analysis on small intestinal crypts from 8-week-old WT and PiZ male mice. Gene expression analysis revealed 193 significantly differentially expressed genes (DEGs), 107 of which were upregulated and 86 of which were downregulated in PiZ mice. Pathway enrichment analysis revealed that the top 3 upregulated KEGG pathways in PiZ mice were protein processing in the ER, proteasome, and antigen processing and presentation (Fig. 3g and Supplementary Table 1), which are indicative of ER stress, proteasomal activation, and increased adaptive immune responses, respectively, suggesting that the accumulation of polymeric hATZ in IECs is associated with selected proteostasis-related responses, including intracellular polymer accumulation and enrichment of ER protein-processing and proteasomal pathways, as described in the PiZ liver. These results were further supported by proteomic analysis showing enrichment of proteins involved in protein processing in the ER (Supplementary Fig. 3a and Supplementary Table 2) and by immunoblot analysis showing increased activation of ER stress and unfolded protein response (UPR) markers, including the P-PERK/PERK ratio, the P-eIF2α/eIF2α ratio, and CHOP (Fig. 3h). In contrast, the other 2 branches of the UPR (IRE1α and ATF6α) appeared to be only minimally involved (Supplementary Fig. 3b). Overall, these results highlight ATZ-dependent cellular defects in intestinal cells.

While PiZ mice display a marked increase in susceptibility to intestinal injury, they do not develop spontaneous colitis over time. Although no evidence of colitis was observed in 80-week-old PiZ mice, aging was associated with progressive epithelial abnormalities, as demonstrated by the reduction in villi-to-crypt length (Supplementary Fig. 4a–d) and exacerbation of goblet (Supplementary Fig. 4e, f) and PC defects (Supplementary Fig. 4g, h), which is consistent with progressive age-associated epithelial alterations in the presence of sustained ATZ expression. This pattern aligns with many IBD susceptibility models in which genetically encoded epithelial vulnerabilities require environmental or inflammatory triggers to elicit overt disease.^22–24^

Taken together, our results support a model in which AATD acts as a disease modifier that sensitizes the intestinal epithelium to injury via PC ER stress, thereby increasing susceptibility to colitis without implying spontaneous disease in the absence of additional triggers.

### Impact of mutant ATZ on human tissue and iPSC-derived small intestinal organoids

**Fig. 4:**
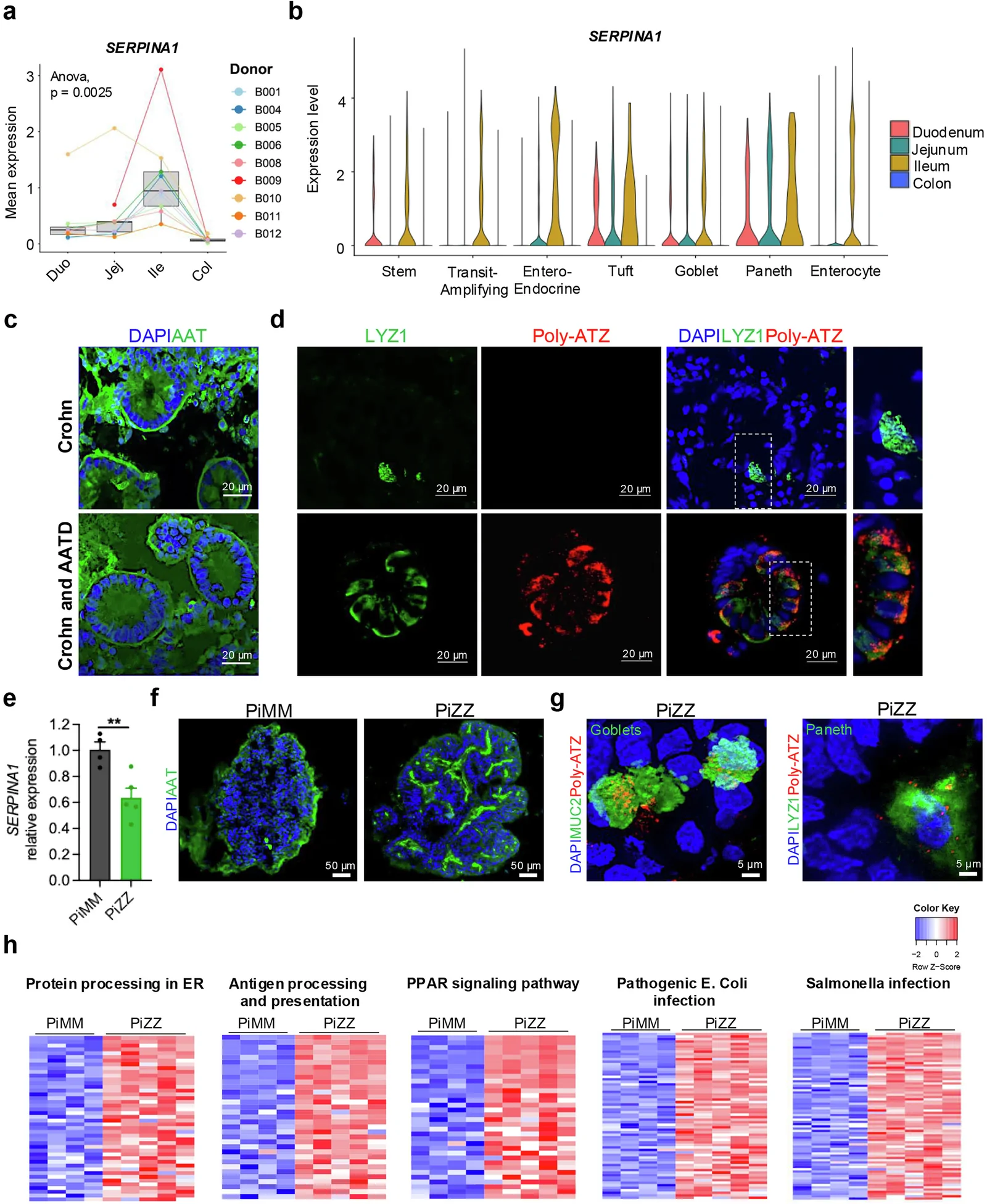
Polymeric ATZ Accumulates in AATD-affected intestinal tissue and hSIO. **a** *SERPINA1* expression levels in the duodenum (Duo), jejunum (Jej), ileum (Ile), and colon (Col) of the human intestine. **b** Cell type-specific expression of *SERPINA1* across intestinal regions. Violin plots showing the distribution of *SERPINA1* expression levels across distinct epithelial cell types within the duodenum, jejunum, ileum, and colon based on single-cell RNA sequencing. **c** Representative immunofluorescence image showing AAT expression in ileal biopsies of Crohn’s disease- and AATD/Chron-affected patients. **d** Representative immunofluorescence images showing LYZ1 and poly-ATZ expression and accumulation in ileal biopsies of Crohn’s disease- and Crohn’s disease-/AATD-affected patients. **e** *SERPINA1* transcript levels normalized to those of *S16* in the Pi*MM and Pi*ZZ hSIOs. **f** Representative immunofluorescence images showing AAT accumulation in cryosections of Pi*MM and Pi*ZZ hSIOs. **g** Representative coimmunofluorescence images of cryosections stained for poly-ATZ and either MUC2 or LYZ1 in Pi*MM and Pi*ZZ organoids. **h** Heatmaps for genes involved in the indicated pathways that are differentially regulated in **t**he Pi*MM and Pi*ZZ hSIO groups. Each dot represents an individual sample. Data are presented as the mean ± standard error. Data normality was assessed using the Shapiro‒Wilk test. Statistical significance was determined using one-way ANOVA or an unpaired two-tailed *t*-test with Welch’s correction (**e**): *p < 0.05, **p < 0.01, ***p < 0.001, and ****p < 0.0001

To address potential species- or tissue-specific differences, we examined publicly available human single-cell atlases. With respect to the intestinal epithelium, the abundance of *SERPINA1* transcripts varied among the epithelial lineages, with more consistent expression in the jejunum and ileum and detectable signals in the enteroendocrine, tuft, goblet, PCs, and enterocytes of both the small intestine and the colon (Fig. 4a, b). Although transcript levels were generally lower in the colon, AAT protein was readily detected in human colon tissue by both immunofluorescence and immunoblot analyses (Supplementary Fig. 5a, b), supporting the presence of AAT protein in the human colonic epithelium.

Notably, AAT expression was confirmed by immunofluorescence analysis of biopsies from CD patients (Fig. 4c). Moreover, ATZ polymers were detected by immunostaining in intestinal crypts from a single AATD patient with CD (Fig. 4d).

To determine whether ATZ is expressed and forms polymers in the human IECs of AATD patients, we generated human small intestinal organoids (hSIOs) from human Pi*MM and isogenic Pi*ZZ iPSCs^25^ using a previously described protocol^26^ (Supplementary Fig. 5c, d). This hSIO model provided a physiologically relevant 3D in vitro setting to study the impact of mutant ATZ on human IECs.

We assessed AAT expression in hSIOs by quantifying *SERPINA1* transcript levels in Pi*MM and Pi*ZZ organoids (Fig. 4e) and confirmed protein expression by immunostaining (Fig. 4f). To determine whether ATZ forms polymers in human IECs, we performed coimmunostaining with cell type-specific markers for EECs, goblet cells, and PCs, along with a polymer-specific ATZ antibody. Consistent with the findings in PiZ mice, ATZ polymers were detected in goblet cells and PCs (Fig. 4g). However, we were unable to identify EECs in our hSIOs. This may be attributed to the fact that EECs were not detected in our hSIOs, likely because of their known rarity in iPSC-derived intestinal organoids, which limits their study in this model system.

To explore the broader consequences of ATZ accumulation, we performed transcriptomic profiling of Pi*MM and Pi*ZZ hSIOs. We identified 4,206 DEGs, with 2525 genes upregulated and 1681 genes downregulated in the Pi*ZZ hSIO. Pathway enrichment analysis revealed significant upregulation of genes associated with protein processing in the ER and inflammatory pathways, including antigen processing and presentation, PPAR signaling, and bacterial infection responses, paralleling the inflammatory signature seen in PiZ mice (Fig. 4h and Supplementary Tables 3 and 4). Conversely, key pathways involved in cell cycle regulation, DNA repair, and intestinal stem cell maintenance, such as WNT signaling, were strongly downregulated (Supplementary Table 3), suggesting impaired epithelial renewal and homeostasis.

### Defective Paneth cell homeostasis and lysozyme secretion in PiZ mice

Electron microscopy (EM) analysis revealed enlarged goblet cells in PiZ mice compared with control mice, characterized by a greater number of electron-lucent mucin granules and partial discharge toward the lumen (Supplementary Fig. 6a). WT mice displayed fewer, denser, and more compact granules, which is consistent with normal mucin synthesis and turnover. However, although these ultrastructural changes indicate goblet cell hyperplasia in PiZ mice, no increase in intestinal permeability was observed in 8-week-old PiZ mice, as demonstrated by a FITC-dextran assay (Supplementary Fig. 6b). Hence, we focused on PC dysfunction as the major contributor to intestinal susceptibility to colitis.

PCs play a critical role in maintaining intestinal immune homeostasis by secreting antimicrobial peptides, including α-defensins, lysozyme, and phospholipase A1, which help regulate the intestinal microbiota and prevent pathogen overgrowth.^27^ Defects in PC homeostasis have been implicated in IBDs and are associated with increased susceptibility to DSS-induced colitis in mice.^17,28,29^ Hence, we focused our attention on alterations in the PCs of PiZ mice.

**Fig. 5:**
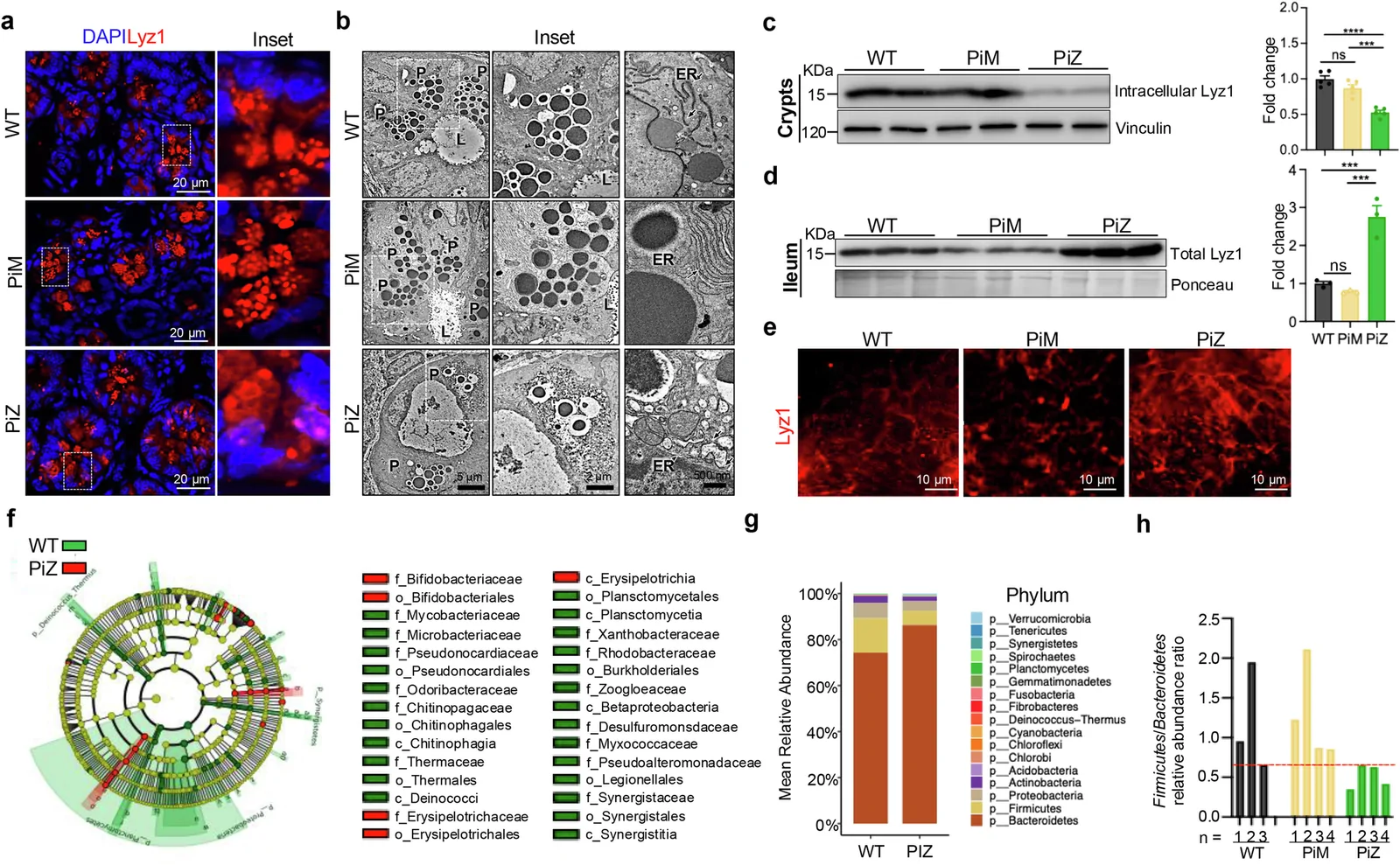
ER Stress-Induced Lysozyme Secretion and Microbiota Alterations in PiZ Mice. **a** Representative immunofluorescence images showing Lyz1 expression in ileal sections from WT, PiM, and PiZ mice. **b** Representative electron microscopy images of the ileal crypts and PCs of WT, PiM, and PiZ mice. The arrows indicate the endoplasmic reticulum (ER). P = PCs; L = lumen. **c** Immunoblot for Lyz1 in the intracellular protein fraction of ex vivo WT, PiM, and PiZ small intestinal crypts plus relative quantification. Vinculin was used as a loading control. **d** Immunoblotting for Lyz1 in the intracellular protein fraction of ileum tissues from WT, PiM, and PiZ mice, plus relative quantification. Ponceau was used as a loading control. **e** Representative immunofluorescence images showing Lyz1 expression in the whole-mount ileum of WT, PiM, and PiZ mice. **f** Cladogram showing the relative bacterial abundance in WT and PiZ mice. **g** Relative *phylum* abundance in WT and PiZ mice. **h** qPCR for *Firmicutes* and *Bacteroidetes* was performed on DNA extracted from the feces of WT, PiM, and PiZ mice. Each dot represents an individual sample. All the data refer to male mice. Data are presented as the mean ± standard error. Data normality was assessed using the Shapiro‒Wilk test. Statistical significance was determined using an unpaired one-way ANOVA. ns not significant, *p < 0.05, **p < 0.01, ***p < 0.001, and ****p < 0.0001

Histological analysis of small intestinal crypts from PiZ mice revealed striking abnormalities in PC morphology, including an abnormal granule appearance compared with that of controls (Fig. 5a). Interestingly, we observed diffuse Lyz1 staining in crypts from PiZ mice. EM analysis provided further insights into these defects, revealing severe ultrastructural abnormalities in PiZ PCs, such as disruption of the ER and accumulation of cytoplasmic vesicles (Fig. 5b), suggesting alterations in the ER‒Golgi axis. These features closely resemble PC defects previously described in CD patients, reinforcing a potential link between AATD and intestinal inflammation.

EM analysis also revealed structural abnormalities in PC secretory granules. Granules appeared well-formed, electron-dense, and enclosed by intact membranes in WT and PiM PCs. In contrast, PiZ PCs exhibited aberrant granule morphology, characterized by membrane-bound compartments that were only partially filled and displayed a dense core surrounded by an electron-lucent peripheral halo, suggesting abnormal Lyz1 secretion.

To further assess this hypothesis, we performed immunoblotting for Lyz1 in both isolated intestinal crypts and whole ileal tissue, which includes extracellular components. Interestingly, compared with control crypts, PiZ crypts presented reduced intracellular Lyz1 levels (Fig. 5c), whereas total Lyz1 levels were elevated in whole ileum samples from PiZ mice under basal conditions (Fig. 5d). Whole-mount staining of the ileum further revealed increased Lyz1 signals within the mucus of PiZ mice, which is consistent with secretory dysfunction (Fig. 5e). Furthermore, a secretory assay using carbachol stimulation in crypts isolated from WT and PiZ mice confirmed that Lyz1 secretion was enhanced in the PiZ model, as suggested by reduced intracellular Lyz1 (Supplementary Fig. 6c, d), further supporting the hypothesis of increased lysozyme release.

Lysozyme has been reported to shape the intestinal bacterial landscape under steady-state conditions.^30^ Notably, our metagenomic analysis of fecal samples from young and old WT and PiZ male mice revealed significant dysbiosis in PiZ mice (Fig. 5f and Supplementary Fig. 4i), likely driven by a reduction in the abundance of anti-inflammatory *Firmicutes* families and a reciprocal expansion of *Bacteroidetes* in PiZ mice but not in WT and PiM control mice (Fig. 5g, h; Supplementary Fig. 4j and Supplementary Table 5), which is consistent with the findings in Lyz1 knock-in (KI) transgenic mice.^30^

Interestingly, in vivo administration of exogenous lysozyme further exacerbated both intestinal and hepatic fibrosis in PiZ mice. Following DSS exposure in the drinking water for 7 days (Supplementary Fig. 7a), compared with the untreated control mice, the lysozyme-treated PiZ-treated mice displayed significantly worsened BW loss (Supplementary Fig. 7b). Despite the lack of significant differences in colon and intestinal length (Supplementary Fig. 7c–e), compared with the control treatment, PiZ-DSS treatment with exogenous lysozyme worsened the histopathological score of the colon (Supplementary Fig. 7f) and was accompanied by increased liver fibrosis, despite no changes in tissue damage or ATZ accumulation (Supplementary Fig. 7g).

These findings suggest that lysozyme, in addition to its antimicrobial role, may act as a pathogenic mediator linking intestinal secretory dysfunction to liver fibrosis in AATD.

### ER stress contributes to altered Lyz1 trafficking and secretion in PiZ crypts

A recent study demonstrated that under ER stress conditions, lysozyme, normally secreted via the canonical ER-Golgi pathway, can be rerouted through the secretory autophagy pathway.^31^ On the basis of the ER stress signatures and ultrastructural abnormalities observed in PiZ crypts, we investigated whether Lyz1 trafficking was altered in association with autophagy-related compartments.

**Fig. 6:**
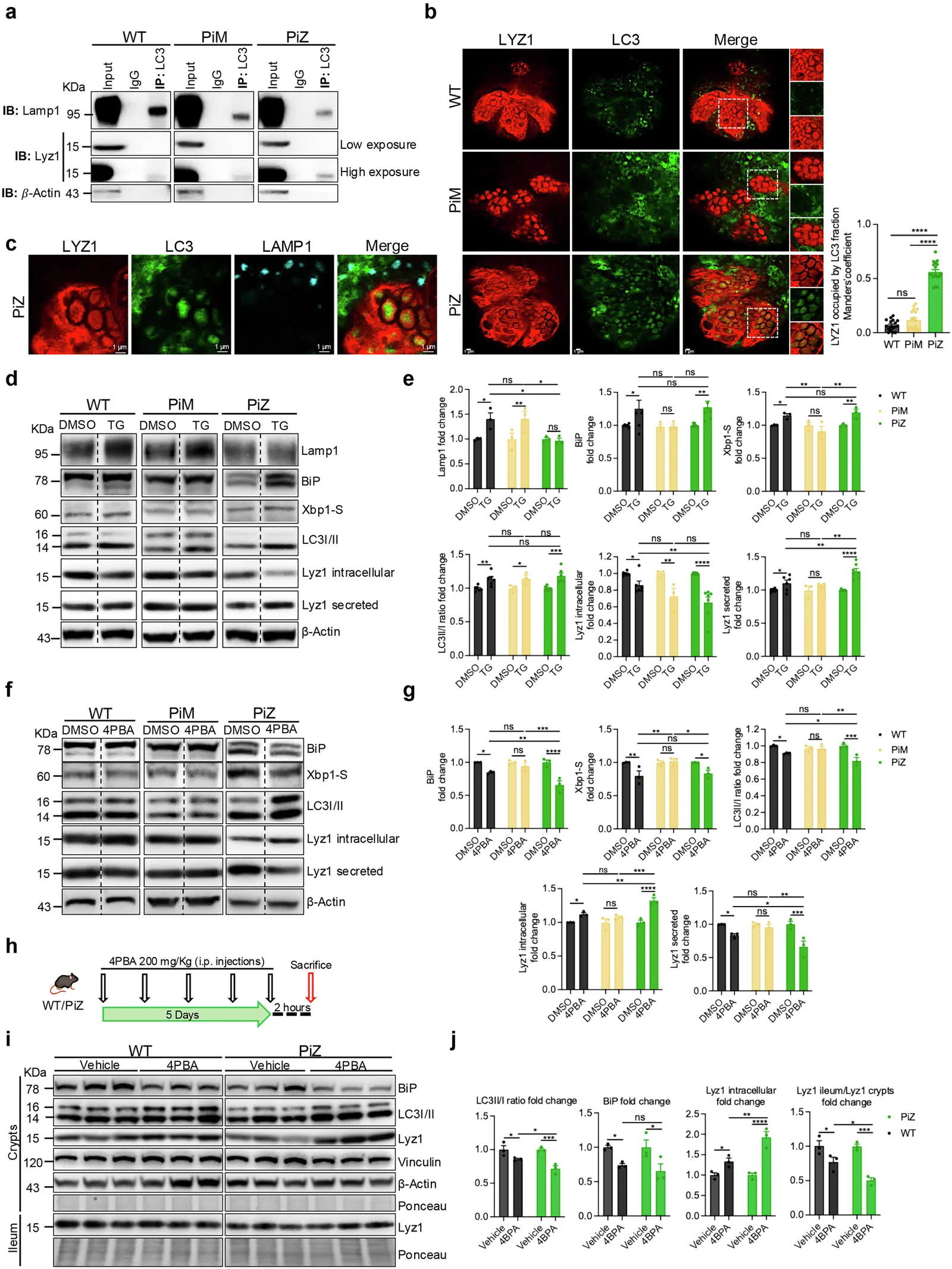
ER stress is associated with altered Lyz1 trafficking through LC3-containing compartments in PiZ crypts. **a** Coimmunoprecipitation of LC3 from intestinal crypt lysates of WT, PiM, and PiZ mice, followed by immunoblot analysis of Lyz1 and LAMP1. IgG immunoprecipitation was used as a negative control. **b** Representative coimmunofluorescence images of LC3 and Lyz1 in intestinal sections from WT, PiM, and PiZ mice and relative quantification. Each dot represents one Lyz-containing granule. PiZ crypts show increased spatial overlap between Lyz1-positive granules and LC3-positive structures compared with those of the WT and PiM controls. **c** Representative coimmunofluorescence images of Lyz1, LC3, and LAMP1 in intestinal sections from PiZ mice, showing limited spatial overlap of LAMP1-positive lysosomal compartments with LC3-positive structures or Lyz1-positive granules. **d**, **e** Immunoblot and quantification of the indicated antibodies on intracellular and secreted protein fractions of ex vivo WT, PiM, and PiZ small intestinal crypts. Crypts were treated in the absence or presence of thapsigargin (TG) (500 nM). Beta-Actin was used as a loading control. **f**, **g** Immunoblot and quantification of the indicated antibodies on intracellular and secreted protein fractions of ex vivo WT, PiM, and PiZ small intestinal crypts. Crypts were treated in the absence or presence of 4-phenylbutyric acid (4-PBA) (10 mM). Beta-Actin was used as a loading control. **h** Schematic representation of WT and PiZ treated with 4-PBA. Immunoblot analysis of the indicated markers (**i**) and corresponding quantifications (**j**) in intracellular protein fractions from small intestinal crypt and ileum tissues isolated from in vivo-treated WT and PiZ mice. Each dot represents an individual sample. All the data refer to male mice. Data are presented as the mean ± standard error. For each genotype, the densitometric values in the TG- or 4-PBA-treated condition were normalized to those in the corresponding untreated control, which was set to 1. Comparisons, therefore, reflect treatment-induced changes within each genotype rather than absolute differences between genotypes run on separate gels. Data normality was assessed using the Shapiro‒Wilk test. Statistical significance was determined using two-way ANOVA or one-way ANOVA (6B): ns not significant; *p < 0.05; **p < 0.01; ***p < 0.001; and ****p < 0.0001

To test this hypothesis, we performed a coimmunoprecipitation assay for LC3, which revealed a significant increase in Lyz1 associated with LC3 in PiZ crypts compared with that in WT and PiM controls (Fig. 6a), indicating preferential entrapment of Lyz1 in LC3^+^ autophagosomes in the PiZ model. These findings were further supported by coimmunofluorescence analysis of Lyz1 and LC3 in intestinal sections from WT, PiM, and PiZ mice. PiZ crypts showed marked colocalization of Lyz1 with LC3^+^ structures, whereas LC3^+^ vesicles adjacent to or colocalizing with Lyz1^+^ granules were rarely detected in WT and PiM samples (Fig. 6b). In addition, LC3 coimmunoprecipitated with higher levels of LAMP1 in WT and PiM crypts, which is consistent with efficient autophagosome‒lysosome fusion. In contrast, LC3-LAMP1 association was markedly reduced or undetectable in PiZ crypts (Fig. 6a, c). Together, these findings indicate altered partitioning of Lyz1 within LC3-associated compartments and reduced association of LC3-containing structures with LAMP1^+^ compartments in PiZ crypts, suggesting that LC3^+^ vesicles containing Lyz1 are less frequently degraded and rerouted to the plasma membrane for secretion. In support of this interpretation, autophagy flux experiments in crypts isolated from WT and PiZ mice treated with chloroquine (CHQ) confirmed reduced lysosomal degradation in the PiZ samples (Supplementary Fig. 8a). Consistent with these findings, immunoblot analysis of ileal lysates from WT, PiM, and PiZ mice revealed greater accumulation of SQSTM1/p62 and LC3 in PiZ mice than in WT and PiM control mice (Supplementary Fig. 8b).

To investigate whether ER stress drives Lyz1 secretion, we isolated PC-enriched crypts from WT, PiM, and PiZ mice and treated them with a pharmacological modulator of ER stress, thapsigargin (TG), an ER stress inducer, and 4-phenylbutyric acid (4-PBA), a chemical chaperone that alleviates ER stress.^32^ Compared with those in the WT and PiM counterparts, the induction of ER stress in the TG significantly increased Lyz1 secretion in the PiZ crypts. Consistent with the increase in secretory autophagy, TG treatment increased LC3 levels in the WT, PiM, and PiZ crypts, whereas LAMP1 expression did not increase accordingly in the PiZ, supporting the presence of an autophagy degradation defect (Fig. 6d, e). In line with the relative protection of PiM crypts from proteostatic stress, as previously reported in the liver,^33^ TG treatment failed to induce BiP and spliced XBP1 (XBP1-S) upregulation in PiM crypts but increased LC3II and LAMP1 levels, suggesting a more efficient adaptive response and recovery from chemically induced ER stress (Fig. 6d, e). In contrast, treatment with 4-PBA reduced Lyz1 secretion in the PiZ crypts, leading to increased intracellular Lyz1 retention (Fig. 6f, g), with only minimal changes in the WT crypts and no detectable effect in the PiM crypts. Since Lyz1 mRNA levels were unchanged (Supplementary Fig. 8c), these findings support a posttranscriptional mechanism and confirm the contribution of ER stress to the dysregulated lysozyme secretion phenotype. Compared with those from the control mice, the crypts from the PiZ mice treated in vivo with 4-PBA consistently increased intracellular Lyz1 levels (Fig. 6h–j), with a much weaker effect on the WT mice. These data further support the restoration of lysozyme retention upon ER stress attenuation.

**Fig. 7:**
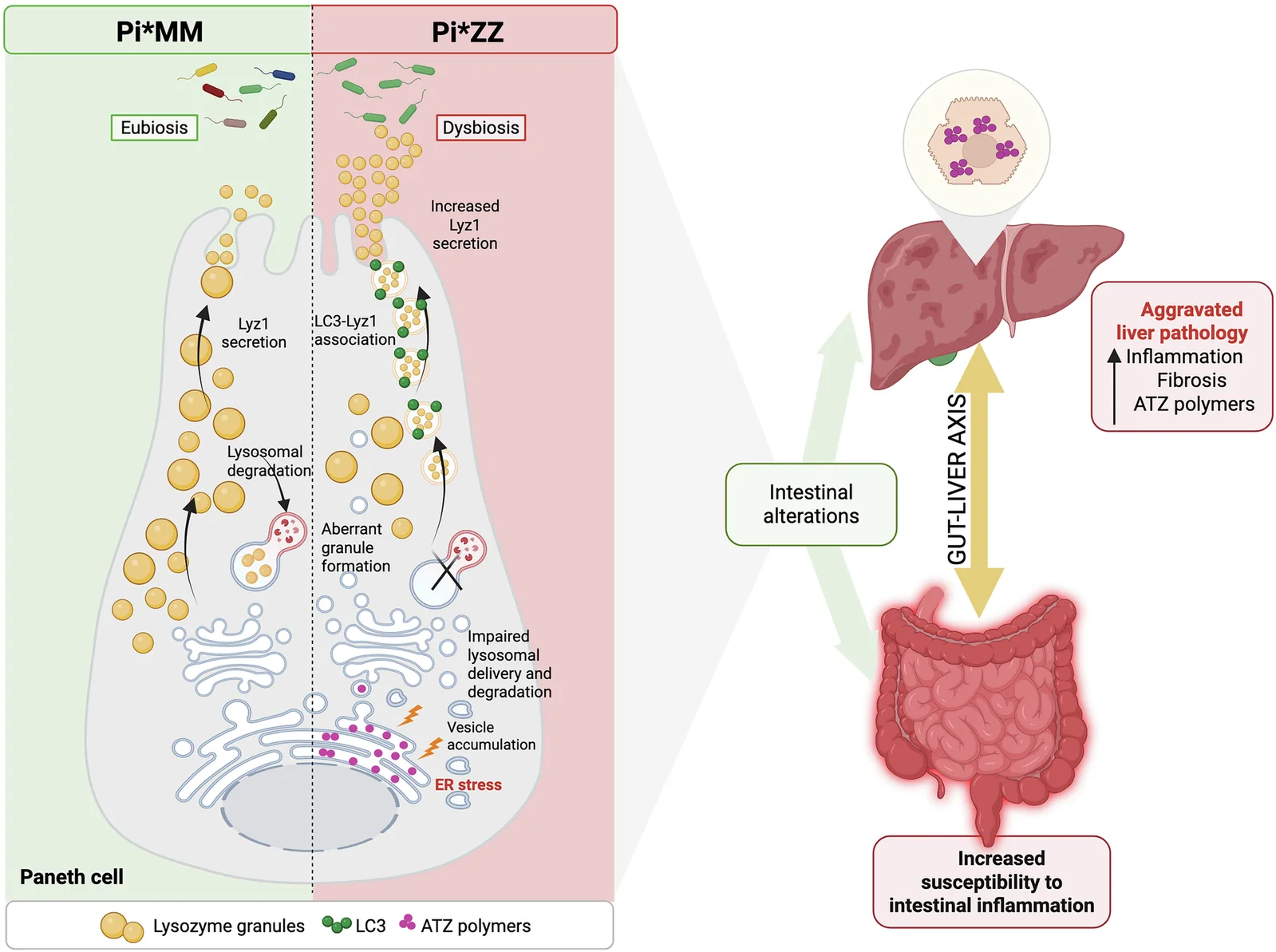
Proposed model of intestinal epithelial alterations associated with polymeric ATZ accumulation. In Pi*MM* Paneth cells, Lysozyme-1 (Lyz1) is packaged into secretory granules, delivered to lysosomal compartments for degradation when required, and released through the conventional secretory pathway, thereby contributing to intestinal microbial homeostasis. In Pi*ZZ* Paneth cells, intracellular accumulation of polymeric ATZ is associated with ER stress, vesicle accumulation, abnormal granule formation, impaired lysosomal delivery and degradation, and increased association of Lyz1 with LC3-containing compartments. These alterations are accompanied by increased Lyz1 release and changes in intestinal microbiota composition. At the organismal level, intestinal epithelial dysfunction in the PiZ model is associated with increased susceptibility to experimentally induced intestinal inflammation and with aggravated hepatic pathology, including inflammation, fibrosis, and increased ATZ polymer accumulation, supporting a potential contribution of the gut–liver axis to disease severity

Overall, our results support the contribution of ER stress to altered Lyz1 trafficking and secretion in PiZ PCs. The increased association of Lyz1 with LC3-containing compartments, together with impaired autophagy-related degradation, is compatible with the engagement of an unconventional autophagy-related trafficking route (Fig.7).

## Discussion

IBDs, including CD and UC, are characterized by symptoms such as diarrhea, abdominal pain, and bloody stools^34^ and are driven by a combination of genetic predisposition, environmental factors, and immune dysregulation.^35^

Alpha-1 antitrypsin (AAT), a serine protease inhibitor primarily produced by hepatocytes, is also expressed in various immune and intestinal cells.^36,37^ Its main role is to protect tissues from the proteolytic activity of neutrophil elastase.^38^ While AAT deficiency (AATD) is classically associated with emphysema and liver disease,^39–44^ AAT also has protective effects on extrapulmonary and extrahepatic sites, including the intestine.^12^ Previous studies have suggested that AAT supplementation can mitigate colitis severity and improve outcomes.^20^

Although IBD is not currently recognized as a classical clinical manifestation of AATD, several epidemiological and experimental observations have suggested a possible association between severe *SERPINA1* deficiency and gastrointestinal disease. Earlier studies reported an increased prevalence of Pi*Z-containing genotypes among subsets of patients with UC and described a more severe clinical course in patients affected by both AATD and colitis.^9–13^ More recent population-based analyses have also suggested an increased incidence of gastrointestinal disorders in individuals with severe AATD.^13^ Mechanistically, several lines of evidence further support a direct connection between AAT dysregulation and intestinal inflammation. Proteolytically cleaved AAT fragments, such as the C-36 peptide, can act as partial TLR4/CD14 agonists and trigger NF-κB-dependent cytokine release, demonstrating that altered AAT species can acquire proinflammatory functions.^14^ *SERPINA1* is also upregulated in active UC and decreases in patients who respond to anti-TNF or corticosteroids, indicating that it is a disease-activity-linked gene.^15^ In patient-derived intestinal organoids, bacterial stimulation induces epithelial *SERPINA1* expression, underscoring an epithelial-intrinsic AAT stress-response pathway.^45^ Finally, genomic studies have identified *SERPINA1* among variants contributing to IBD-associated pathways at the population level.^46^ Collectively, these studies support clinical plausibility and justify further investigation.

A previously proposed mechanism linking AATD to IBDs was analogous to that of AATD-related emphysema. It was hypothesized that the Z mutation in the *SERPINA1* gene reduces AAT circulating levels, which in turn diminishes the ability to inhibit serine protease-mediated intestinal damage. Consequently, UC patients with reduced AAT levels are less protected from neutrophil-mediated tissue injury, particularly during acute disease episodes. However, these studies were primarily descriptive and did not identify the underlying mechanism linking AATD and IBDs.

Our study extends previous observations by demonstrating a PC-specific defect driven by ATZ accumulation and ER stress and provides direct evidence that AATD contributes to IBD pathogenesis.

Our findings demonstrate that compared with control mice, PiZ mice, a well-established AATD mouse model, exhibit increased susceptibility to DSS-induced colitis characterized by significant weight loss, shortened colon length, and increased histopathological damage. The exacerbated colitis phenotype in PiZ mice suggests that AAT is critical for maintaining intestinal immune homeostasis. In support of this hypothesis, DSS-treated PiZ mice displayed increased inflammatory cytokine expression and hepatic fibrosis, indicating that the systemic inflammatory response was linked to AATD.

PCs, specialized secretory epithelial cells residing in small intestinal crypts, contribute to intestinal defense through the secretion of antimicrobial peptides, including α-defensins, lysozyme, and phospholipase A1. Dysfunctional PCs have been implicated in IBD pathogenesis and enhanced susceptibility to DSS-induced colitis.^17,28,29^ In our model, PiZ mice presented significant abnormalities in the number, organization, and morphology of PCs. We identified ATZ polymers within PCs that trigger ER stress and cytoplasmic vesicle accumulation, features reminiscent of the pathology observed in CD.

A central finding is that ATZ-expressing PCs exhibit altered granule morphology, increased lysozyme secretion, and impaired autophagy. Autophagy, which is essential for recycling cellular components^47^ and supporting secretory functions in PCs,^17,48^ is often dysregulated in IBDs.^49^ Key IBD-associated genes, such as nucleotide-binding oligomerization domain containing 2 (NOD2) and autophagy-related 16 like 1 (ATG16L1), govern autophagy pathways, and their mutations result in abnormal PCs^48,50,51^ and defective lysozyme secretion.^31^ Indeed, during intestinal infection, microbes invade PCs, inducing Golgi fragmentation and disrupting conventional cellular secretion. To bypass this blockade, PCs utilize secretory autophagy, packaging lysozyme into double-membrane-bound vesicles for release into the intestinal lumen. This process is regulated by the ER stress response pathway.

In our study, PiZ mice displayed enhanced secretory autophagy despite general autophagy defects, leading to elevated lysozyme secretion under both baseline and stress conditions.

Transcriptomic analysis confirmed the activation of ER stress, proteasomal degradation, and antigen processing in PiZ intestinal crypts, suggesting that ATZ accumulation disrupted PC function and increased inflammation. In this context, the PERK-eIF2α branch under basal conditions and the IRE1-XBP1 axis upon stress induction emerged as particularly relevant, which is consistent with their established role in intestinal secretory cell homeostasis, enteritis, and susceptibility to experimental colitis^23^ and in line with the ER stress response to external challenges, including lipopolysaccharide (LPS), in the livers of PiZ mice.^33^

In this study, we used 4-PBA to reduce ER stress, which supports our conclusions. However, despite being widely used as a chemical chaperone, it is a relatively blunt instrument and has limited translational appeal beyond proof-of-concept. In AATD patients, a short open-label study revealed that oral 4-PBA did not increase circulating AAT and was associated with side effects, underscoring its limitations as a therapy in this setting.^52^ In a clinical setting, several more sophisticated classes of ER stress modulators, such as tauroursodeoxycholic acid (TUDCA), a bile acid derivative with chaperone properties, have been used in clinical trials for ALS,^53^ primary biliary cholangitis,^54^ NASH,^55^ and mitochondrial disorders.^56^ In ATZ cellular models, TUDCA was reported to reduce ATZ accumulation in preclinical studies by reducing ER stress-linked apoptosis.^57^

Importantly, PC dysfunction is not unique to AATD but rather represents a broader ER stress-associated phenotype common to secretory cells in proteostasis disorders. Owing to their high secretory activity, PCs are particularly sensitive to the accumulation of misfolded proteins and maladaptive unfolded protein response activation. Similar abnormalities in PC granule morphology and antimicrobial peptide secretion have been reported in other conditions characterized by protein misfolding, such as cystic fibrosis,^58^ congenital ER quality control defects,^59^ and other proteinopathies, including Aβ^60^ and mutant SOD1,^61^ supporting the concept that intestinal epithelial dysfunction may be a sentinel feature of systemic proteostasis imbalance.

Excessive lysozyme secretion has been shown to promote inflammation via NOD signaling and to exacerbate colitis,^31,62,63^ but its absence is protective.^30^ Moreover, elevated lysozyme disrupts the microbial composition, contributing to dysbiosis,^30^ a hallmark of IBDs and a contributor to liver diseases, such as metabolic dysfunction-associated steatohepatitis (MASH), alcoholic liver disease, primary sclerosing cholangitis, and cirrhosis.^64,65^ In our model, lysozyme-treated PiZ mice displayed worsened intestinal inflammation and increased liver fibrosis, suggesting that lysozyme plays a pathological role in AATD. Indeed, aberrant lysozyme expression has been implicated in the progression of renal fibrosis, such as in diabetic nephropathy, through the promotion of the JAK/STAT3 signaling pathway.^66^

The gut‒liver axis has been increasingly associated with liver diseases.^16^ In the context of AATD, dysbiosis may serve as a key modifier of disease progression, although the contribution of the gut microbiota has remained largely unexplored. In PiZ mice, we observed intestinal architecture alterations, PC hyperactivity, and elevated lysozyme release, suggesting a potential role for gut dysbiosis in driving liver pathology. Further investigation is needed to define how microbial signals shape hepatic inflammation in AATD and whether targeting the microbiota could offer therapeutic benefit.

These findings also raise caution regarding the use of lysozyme supplementation, which is commonly explored as a therapeutic strategy in IBDs,^67^ in the context of AATD. Unlike the previous protective hypothesis, our data suggest that lysozyme supplementation may worsen outcomes in AATD-associated intestinal inflammation and liver injury. Monitoring lysozyme levels could serve as a biomarker for disease severity or progression under fibrotic conditions.

Finally, we validated our findings in hSIOs derived from Pi*ZZ iPSCs. These organoids exhibited ATZ polymer accumulation in secretory IECs and transcriptomic signatures consistent with inflammation and epithelial dysfunction. They represent a powerful model for studying AATD-related intestinal disease and testing novel therapies.

Our study has several important limitations. First, we relied on the PiZ mouse, which carries multiple copies of the mutant *SERPINA1* and may overestimate the polymer load and stress responses. The inclusion of PiM controls, which showed no intestinal phenotype, supports that the effects observed are Z-specific rather than due to overexpression.

Second, human validation was limited to a single patient biopsy, as intestinal sampling in AATD is rare and AATD genotyping is not routinely performed in IBD care. Larger retrospective studies are needed to determine the prevalence and significance of intestinal ATZ polymers.

Third, not all individuals with AATD develop IBDs or gut-related manifestations, suggesting the influence of genetic or environmental modifiers, including host genetic background, microbiota composition, diet, and coexisting inflammatory or metabolic conditions. Defining these modifiers through integrated microbiome and multiomics analyses will be necessary to determine the clinical relevance of this pathway. Overall, these limitations highlight the need for broader validation and support the view that AATD serves as a cofactor that increases susceptibility to colitis in the presence of additional environmental or inflammatory hits.

In conclusion, our study identifies a mechanistic link between AATD, PC dysfunction, and intestinal inflammation, highlighting a potential role for the gut‒liver axis in AATD pathology. These insights warrant greater clinical attention to gastrointestinal manifestations in AATD patients and the reconsideration of lysozyme-based interventions.

## Materials and methods

### Mouse experiments

Animal care and procedures were approved by the Italian Ministry of Health. PiM^18^ and PiZ transgenic mice^4^ were maintained on a C57BL/6J background. Six- to eight-week-old male mice were used for all experiments unless otherwise indicated. Blood samples were collected by retro-orbital bleeding, and serum samples were analyzed by ELISA for ATZ as previously described.^68^ C57BL/6J WT mice were used as controls. All the mice were housed in a pathogen-free facility under standard laboratory conditions (25 ± 1 °C, 12:12-h light/dark cycle). Standard food and water were provided *ad libitum*.

### Intestinal permeability assay

The mice were fasted for 4 h before the intestinal permeability test. During this period, they were kept in a new cage and single-housed to limit coprophagy, with free access to water. After 4 h of fasting, blood (pretest blood) was collected from the tail. The plasma was separated from heparinized whole blood by centrifugation at 5000 rcf for 5 min at 4 °C. The mice were then orally gavaged with fluorescein isothiocyanate (FITC)-dextran, which was diluted to 100 mg/ml in PBS at a dosage of 50 mg/kg BW. After 4 h, we repeated blood collection (posttest blood) and processed the blood samples to collect plasma. Pre- and posttest plasma samples were diluted 1:10 in 1X PBS and transferred to a 96-well plate with a black opaque bottom. Pre- and posttest plasma fluorescence was determined at 530 nm with excitation at 485 nm. Finally, relative fluorescence was determined by subtracting the pretest fluorescence from the posttest fluorescence. The reagents used are listed in Supplementary Table 6.

### DSS-induced acute colitis

Colitis was induced by oral administration of DSS. Briefly, 8-week-old WT and PiZ mice were treated with 3% (w/v) DSS in the drinking water for 7 or 8 days. The control animals received tap water. BW was recorded daily during DSS treatment. On day 7 or 8, the mice were sacrificed, and tissue samples were harvested. For exogenous lysozyme supplementation, recombinant human lysozyme was administered daily by oral gavage at a dosage of 20 mg/kg. The reagents used are listed in Supplementary Table 6.

### Sodium 4-phenylbutyrate (4-PBA) treatment

WT and PiZ mice were divided into four experimental groups: WT treated with vehicle, WT treated with 4-PBA, PiZ treated with vehicle, and PiZ treated with 4-PBA. To inhibit ER stress, 4-PBA (200 mg/kg/day) was administered intraperitoneally for 5 consecutive days. The control groups received an equivalent volume of vehicle solution (0.9% NaCl) via intraperitoneal injection. The reagents used are listed in Supplementary Table 6.

### Histological analysis

Intestine and liver samples were fixed in 4% PFA for 16 h, stored in 70% ethanol and embedded in paraffin blocks. Hematoxylin and eosin (H&E) staining was performed following the World IHC protocol. Bright-field sections were scanned with Zeiss Axioscan Z1. PAS-D staining was performed on 6-μm-thick liver sections. The sections were rehydrated and treated with 0.5% (α-amylase type VI-B) amylase solution for 20 min and then stained with PAS-D reagent according to the manufacturer’s instructions. Sirius red staining was performed on 6-μm liver sections that were rehydrated and stained for 1 h in picro-Sirius red solution (0.1% Sirius red in saturated aqueous solution of picric acid). After two changes of acidified water (5 mL glacial acetic acid in 1 L water), the sections were dehydrated in three changes of 100% ethanol, cleared in xylene, and mounted in a resinous medium. Alcian blue staining was performed on 6-μm-thick paraffin sections of the intestine. The sections were rehydrated and stained for 1 h at room temperature with Alcian blue solution (pH 2.5). Afterward, the slides were washed in running water for 1 min and counterstained with Nuclear Fast Red for 1 min. The slides were rinsed in running water for 3 min, after which the sections were dehydrated in three changes of 100% ethanol, cleared in xylene, and mounted in resinous medium. Bright-field intestinal sections were scanned with Zeiss Axioscan Z1 and analyzed using ImageJ software. Quantification was performed using QuPath v0.5.0 on the segmented images, and the percentage area positively stained was calculated. Bright-field liver sections were scanned with Zeiss Axioscan Z1.

For immunostaining, 6-μm sections were rehydrated, hydrated in 1X PBS (pH 7.4) and permeabilized for 20 min with 0.2% Triton in 1X PBS. Heat-induced epitope retrieval using the citrate buffer method (pH 6.0) was performed to retrieve the antigen sites. The sections were then incubated for 1 h at room temperature with blocking solution (3% BSA, 5% donkey serum, 0.1% Triton in 1X PBS) and overnight at 4 °C with primary antibodies (Supplementary Table 7). The secondary antibodies used in the donkey were AlexaFluor-488 (alternatively 594) anti-rabbit and AlexaFluor-488 (alternatively 594) anti-mouse. The nuclei were counterstained with DAPI. Stained sections were mounted, covered with a coverslip, and examined with a Zeiss Apotome microscope. The reagents used are listed in Supplementary Table 6.

Immunofluorescence analysis for Olfm4 and coimmunofluorescence analysis for LC3, LYZ, and LAMP1 were performed in 6-µm paraffin sections with a VENTANA BenchMark Ultra automated staining instrument (Ventana Medical Systems, Roche) using VENTANA reagents, except as noted, according to the manufacturer’s instructions, with the support of the Advanced Histopathology facility at TIGEM.

Briefly, the slides were deparaffinized using Discovery Wash solution (cat # 950-510) for 16 min at 72 °C. Epitope retrieval was accomplished with CC1 solution (cat # 950–500) at a high temperature (95 °C) for a period that was suitable for a specific tissue type. Antibodies were titrated with a blocking solution into user-fillable dispensers for use on the automated stainer and then developed using the Discovery FAM kit (cat #760-243) according to the manufacturer’s instructions. Nuclei were stained with DAPI (Thermo Fisher Scientific, D1306). Stained liver sections were examined under a Zeiss Axiocam MR microscope or Leica Stellaris confocal microscope.

### Whole-mount ileum lysozyme immunofluorescence

Briefly, 1 cm of distal ileum tissue was dissected, gently flushed with ice-cold PBS-1X to remove luminal contents, and opened longitudinally. Ileum tissues were fixed in 4% paraformaldehyde for 1 h at room temperature (RT), followed by three washes in ice-cold 1X PBS for 5 min each. To reduce nonspecific binding, ileum tissues were blocked in 10% fetal bovine serum (FBS) in 1X PBS for 1 h at RT and then stained with lysozyme primary antibody (1:100) for 2 h at RT (Supplementary Table 7**)**. After primary antibody incubation, the ileum tissues were washed three times for 5 min each with 1X ice-cold PBS (Supplementary Table 7) and incubated with a donkey anti-rabbit Alexa Fluor 594 secondary antibody for 2 h at RT. Finally, the ileum tissues were washed three additional times with 1X ice-cold PBS for 5 min each, mounted with coverslips, and imaged using a Leica DM5500 microscope. The reagents used in this study are listed in Supplementary Table 6.

### Hydroxyproline assay

The hepatic hydroxyproline concentration was quantified as previously described.^69^ Briefly, liver tissue was homogenized and hydrolyzed in 6 N HCl at 110 °C for 16 h. The resulting hydrolysates were filtered and neutralized in citrate-acetate buffer and then incubated with chloramine-T solution (Sigma‒Aldrich) for 20 min at room temperature. Afterward, Ehrich’s reagent (Sigma‒Aldrich) was added, and the samples were incubated at 65 °C for 20 min. The absorbance was recorded at 550 nm, and the hydroxyproline concentration was calculated using a standard curve generated from known concentrations of hydroxyproline.

### Crypt isolation and treatments

After the mice were euthanized, the small intestine was dissected and flushed with 1X ice-cold PBS. The intestine was cut longitudinally, and on a glass microscope slide, the villi were scraped off. Next, the intestine was cut into small pieces of 2–3 cm and transferred to a 50 ml plastic Falcon tube containing 30 ml of ice-cold 1X PBS. After shaking, the pieces were transferred to clean 50 ml plastic Falcon tubes containing 30 ml of 1X ice-cold PBS. After shaking, the tissue was transferred to a clean 50 ml plastic Falcon tube containing 30 ml of 1 × 5 mM EDTA/PBS and placed at 4 °C for 45 min on a rotation shaker for digestion. The supernatant fraction enriched in crypts was collected, passed through a 70 μm cell strainer and centrifuged for 5 min at 450 rcf at 4 °C. The crypts were then pelleted and resuspended in 1 ml of 1X ice-cold PBS. When indicated, intestinal crypts were incubated with 500 nM thapsigargin (TG), 10 mM 4-phenylbutyric acid (4-PBA, and 100 μM chloroquine (CHQ) dissolved in DMEM for 45 min at 37 °C (for carbachol (CCh, 10 mM), the crypts were incubated for 10 min at RT). After incubation, the supernatant was collected by centrifugation at 16,000 rcf for 15 min at 4 °C. Crypt pellets were subsequently washed twice with 1X ice-cold PBS, after which the intracellular proteins were isolated. The reagents used are listed in Supplementary Table 6.

### Immunofluorescence staining of organoid cryosections

Organoids were fixed in 4% paraformaldehyde for 30 min at RT, rinsed three times in 1X ice-cold PBS, and transferred to 10% sucrose for 1 h at RT. After sucrose treatment, the organoids were embedded in optimal cutting temperature (OCT) compound, sectioned at 10 μm, mounted on glass slides and stained as indicated. After equilibrating the sections at room temperature for 30 min, the sections were washed three times for 5 min in 1X ice-cold PBS, permeabilized for 20 min with 0.2% Triton in 1X PBS and blocked with 5% donkey serum, 3% BSA and 0.1% Triton in 1X PBS for 1 h at RT in a humid chamber. The sections were then incubated overnight at 4 °C with primary antibodies (Supplementary Table 7). The secondary antibodies used in the donkey were AlexaFluor-488 (alternatively 594) anti-rabbit and AlexaFluor-488 (alternatively 594) anti-mouse. The nuclei were counterstained with DAPI. Stained sections were mounted, covered with a coverslip, and examined with a Zeiss Apotome microscope. The reagents used are listed in Supplementary Table 6.

### Electron microscopy

Intestinal tissue was fixed for 24 h at 4 °C in a mixture of 2% paraformaldehyde and 1% glutaraldehyde prepared in 0.2 M HEPES buffer (pH 7.4). After dehydration, the samples were embedded in epoxy resin and polymerized at 60 °C for 72 h. Thin 60 nm sections were cut on a Leica EM UC7 microtome. EM images were acquired using an FEI Tecnai-12 electron microscope equipped with a VELETTA CCD digital camera.

### Generation of hISOs

To generate hSIOs from iPSCs, we first directed the iPSCs to differentiate into definitive endoderm (DE) using Activin A for 3 days. DE cells were then exposed to FGF4 for 4 days to induce mid- and hindgut spheroid formation. These spheroids were subsequently embedded in geltrex and cultured with 5% R-spondin-1-conditioned media, Noggin, and EGF to facilitate their maturation into hSIOs.^26^ The reagents used are listed in Supplementary Table 6.

### Real-time PCR

Total RNA was extracted from liver tissue and small intestinal crypts with TRIzol reagent using an RNeasy kit. RNA was reverse transcribed using a first-strand complementary deoxyribonucleic acid kit with random primers according to the manufacturer’s protocol. RT‒PCR was performed on a Roche Light Cycler 480 system. PCR was performed using SYBR Green Master Mix. The PCR conditions for all the tested genes were as follows: preheating, 5 min at 95 °C; cycling, 45 cycles of 15 s at 95 °C, 15 s at 60°C and 25 s at 72 °C. The results are expressed in terms of the cycle threshold (*C*_*t*_). The *C*_*t*_ values were averaged for each duplicate. *S16* was used as an endogenous control gene (reference marker). The data were analyzed using LightCycler 480 software, version 1.5 (Roche Applied Science). The reagents used are listed in Supplementary Table 6. The primers used are listed in Supplementary Table 8.

### Coimmunoprecipitation assay

Intestinal crypt lysates were prepared in cold RIPA lysis buffer (50 mM Tris-HCl, 150 mM NaCl, 1% Triton X-100, 1 mM EDTA, 0.1% SDS, pH 7.4) in the presence of protease and phosphatase inhibitors, incubated for 30 min on ice, and centrifuged at 16,000 rcf for 15 min. Supernatants were collected, and the protein concentration was determined with a Pierce BCA protein assay kit. Next, 50 µL of Dynabeads™ Protein G magnetic beads was incubated for 15 min at RT under gentle rotation with 2 µg of anti-LC3B. As negative controls, 50 µL of Dynabeads™ Protein G magnetic beads was incubated with 2 µg of anti-rabbit IgG isotope control. Next, the Dynabead-antibody complex was incubated with intestinal crypt lysates (250 µg) for 15 min at RT under gentle rotation. Using a DynaMag™ magnet, the beads were then washed three times with 1 mL of 1X PBS for 5 min under rotation, and the immunoprecipitated Lyz1 and LAMP1 were eluted in Laemmli buffer (62.5 mM Tris-HCl, 2% SDS, 10% glycerol, 20 mM DTT; pH 6.8) at 95 °C for 10 min and separated by SDS‒PAGE. The reagents used are listed in Supplementary Table 6.

### Library preparation and bioinformatics workflow

For gene expression analyses, total RNA was extracted according to the manufacturer’s instructions (RNeasy Mini Kit). Total RNA was quantified using a Qubit 4.0 fluorometric assay (Thermo Fisher Scientific). The extracted RNA was precisely quantified and mixed at 10 ng/µL. Libraries were prepared from 125 ng of total RNA using the NEGEDIA Digital mRNA-seq research grade sequencing service (Next Generation Diagnostic, NEGEDIA srl), which included library preparation, quality assessment and sequencing on a NovaSeq 6000 sequencing system using a single-end, 100-cycle strategy (Illumina Inc.). Raw data were analyzed by the Next-Generation Diagnostics SRL proprietary NEGEDIA Digital mRNA-seq pipeline (v2.0), which involves a step of quality filtering and trimming, alignment to the reference genome and counting by gene (BBMap-Bushnell B.sourceforge.net/projects/bbmap/). Trimming and cleaning with bbduk. Illumina NovaSeq 6000 base call (BCL) files were converted into fastq files through bcl2fastq. Alignments were performed with STAR 2.6.0a.^70^ The expression levels of genes were determined with 0.9.1 HTseq counts. The constructed genomes are Hg38 and mm10.

### Transcriptomics of the PiZ intestine

Two unbiased RNA-seq analyses were performed: the first was on the intestine of PiZ animals compared to that of control mice, and the second was on human organoids compared to that of controls. A total of 193 DEGs were found in the mouse dataset (GSE291017). The threshold for the statistical significance of gene expression was an FDR < 0.1. A total of 4206 DEGs were found in the Organoids dataset (GSE291018). The threshold for the statistical significance of gene expression was an FDR < 0.05. Gene Ontology enrichment analysis (GOEA) was performed on the inhibited and induced genes separately by using the DAVID bioinformatics tool, and the output was restricted to the “Kyoto Encyclopedia of Genes and Genomes” (KEGG Pathway) analyses (refer to Supplementary Tables 1 and 3). The thresholds for determining the statistical significance of the GOEA results were an FDR < 0.1 and an enrichment score ³1.5, whereas those for the KEGG pathway analyses were an FDR < 0.1. For the GOEA of the DEGs of the Organoids, a log_2_FC > 1.5 and a log_2_FC < −1.5 were used as the thresholds for the selection of genes with inhibited and induced expression.

### LC‒MS/MS analysis

Intestinal crypt lysates were prepared in cold RIPA lysis buffer (50 mM Tris-HCl, 150 mM NaCl, 1% Triton X-100, 1 mM EDTA, 0.1% SDS, pH 7.4) in the presence of protease and phosphatase inhibitors, incubated for 30 min on ice, and centrifuged at 16,000 × *g* for 15 min. Supernatants were collected, and the protein concentration was determined by a Pierce BCA protein Assay Kit. The proteins were then precipitated in acetone and reduced and alkylated in a solution of 6 M guanidine-HCl, 5 mM TCEP, and 20 mM chloroacetamide. Peptides were obtained by digesting proteins with LysC (WAKO) for 3 h at 37 °C and with sequencing-grade endopeptidase trypsin (Promega) overnight at 37 °C. The collected peptide mixtures were concentrated and desalted using the Stop and Go Extraction (STAGE) technique. Instruments for LC‒MS/MS analysis consisted of a NanoLC 1200 coupled via a nanoelectrospray ionization source to a quadrupole-based Q Exactive HF benchtop mass spectrometer. For chromatographic separation, a binary buffer system consisting of 0.1% formic acid (solution A) and 80% acetonitrile (solution B) and 0.1% formic acid was used. The peptides were separated according to their hydrophobicity on an analytical column (75 μm) packed in-house with C18-AQ 1.9 μm C18 resin with a gradient of 7–32% solvent B for 45 min, 32–45% B for 5 min, 45–95% B for 3 min, and 95–5% B for 5 min at a flow rate of 300 nl/min. MS data acquisition was performed in DIA (Data Independent Acquisition) mode using 32 variable windows covering a mass range of 300–1650 *m*/*z*. The resolution was set to 60,000 for MS1 and 30,000 for MS2. The AGC was 3e6 in both MS1 and MS2, with a maximum injection time of 60 ms in MS1 and 54 ms in MS2. The NCEs were set to 25%, 27.5%, and 30%. With respect to the transcriptome, GOEA was also performed on induced proteins that restricted the output to KEGG pathways (refer to Supplementary Table 2).

### Single-cell RNA-seq data processing

Publicly available single-cell RNA-sequencing data from the murine intestine^21^ were processed in R (v3.4.1) using the Seurat R package (v3.0.2). The expression matrix and corresponding metadata were loaded and filtered to retain only cells with matching annotations. A Seurat object was created after cells with at least 200 detected genes were filtered out. To assess the regional expression patterns of selected genes in young mice, we performed a compartment-specific analysis using filtered epithelial single-cell RNA-seq data (as described above). Cells from animals labeled “Young” were considered subsets, and further analyses excluded the cecum to ensure comparability across small and large intestinal compartments. The expression levels of *Serpina1* gene family members (*Serpina1a*–*Serpina1f*) were visualized in each intestinal compartment using violin plots stratified by cell type. To quantify and compare expression levels, we calculated the average expression of each gene per compartment and sample using Seurat’s average expression function. Statistical comparisons were conducted on average expression values using one-way ANOVA across compartments. Plots were created with the ggplot2 and ggpubr R packages, showing boxplots with overlaid jittered sample points and dashed lines connecting paired samples.

### Single-cell RNA-seq analysis of *SERPINA1* expression in the human intestinal epithelium

Preprocessed Seurat objects representing epithelial cells from the human duodenum, jejunum, ileum, and colon were analyzed using R (v4.1.3) and the Seurat package (v4.3.0.1). Regional datasets were imported, annotated with their corresponding intestinal region, and merged using the “merge” function. Cell-type annotations from individual regions were harmonized into seven major epithelial categories: stem, transit-amplifying (TA), enteroendocrine, tuft, goblet, Paneth, and enterocyte. Cells labeled “Unknown” or “Specialized Secretory” were excluded from further analysis. *SERPINA1* expression was quantified at the single-cell level and summarized per donor and intestinal region. Regional expression differences were evaluated using one-way ANOVA as implemented in the ggpubr package (v0.6.0). Visualization of *SERPINA1* expression was performed using violin plots (“VlnPlot”) and boxplots displaying donor-level averages and variability across intestinal regions and epithelial cell types.

### Metagenomic analysis

Fecal samples were collected by placing each mouse into an empty, autoclaved, metabolic cage for 24 h. The pellets were collected into sterile tubes and fresh-frozen.

Fecal DNA was extracted using a Stool DNA Isolation Kit following the manufacturer’s protocol. Fecal DNA was quantified using a Qubit 4 fluorometric assay. Libraries were prepared from 100 ng of total DNA using the Negedia Whole Genome Sequencing service (Next Generation Diagnostics s.r.l.), which included library preparation with terminal end ligation, quality assessment and sequencing on a NovaSeq 6000 sequencing system using a paired-end, 2 × 150 cycle strategy (Illumina Inc.). The reagents used are listed in Supplementary Table 6.

### Microbiome taxonomic profiling and differential abundance analysis

The microbial community composition was assessed using Kraken2, and the taxonomic profiles were processed using KrakenTools.^71^ Kraken2 report files were converted into MetaPhlAn-compatible format using kreport2mpa.py with the -percentages option enabled to retain relative abundances. All MPA-formatted outputs were subsequently merged using the combine_mpa.py script to generate a single abundance matrix. This merged file served as the input for downstream differential abundance testing. To identify *taxa* significantly enriched between experimental groups, linear discriminant analysis effect size (LEfSe) was employed using a BioBakery Docker image (version 1.0.0_dev_9adc3a62460e). The MPA-formatted abundance table was first reformatted using format_input.py with the following parameters: “-c 1 -s 2 -u 3 -o 1000000,” and LEfSe analysis was then conducted via run_lefse.py. Visualization of the results included the generation of both bar plots and cladograms using plot_res.py and plot_cladogram.py. These outputs facilitated the interpretation of the taxa most differentially abundant between conditions.

### qPCR analysis of the *Firmicutes*-to-*Bacteroidetes* ratio in fecal samples

Fecal DNA was extracted as described above. qPCR was performed using SYBR Green Master Mix with the following conditions: preheating, 5 min at 95 °C; cycling, 45 cycles of 15 s at 95 °C, 15 s at 60 °C and 25 s at 72 °C. The relative abundance of *Firmicutes* and *Bacteroidetes* was evaluated using cycle threshold (*C*_*t*_) values. *C*_*t*_ values from duplicate samples were averaged and normalized to those of the universal bacterial 16S rRNA gene. The *Firmicutes*-to-*Bacteroidetes* ratio was calculated as the relative abundance of *Firmicutes* divided by that of *Bacteroidetes*. The data were analyzed using LightCycler 480 software version 1.5 (Roche Applied Science). The reagents and primers used are listed in Supplementary Tables 6 and 9, respectively.

### Human samples

Intestinal tissues were collected after written informed consent was obtained and in accordance with protocols approved by the Ethics Committee (Prot. N. 00032327). Intestinal biopsy was obtained from a patient with a homozygous PiZ/Z genotype who presented with obstructive respiratory dysfunction and radiological evidence of emphysema with interstitial involvement. The patient had a history of severe anemia treated during hospitalization with blood transfusion and iron supplementation and a previous ileal resection for intestinal infarction. Endoscopic evaluation at the time of sampling revealed gastroduodenopathy, ileal ulcerations, and nonspecific colopathy.

Colon biopsy samples from healthy donors and ileum samples from a CD patient were also used in the study. Biopsies (0.5 cm) were fixed in formalin for 2 h at RT, rinsed three times in 1X ice-cold PBS, and transferred to 20% sucrose overnight at 4 °C. After sucrose treatment, the ileal biopsy was embedded in OCT compound, sectioned at 10 μm, mounted on glass slides and stained as indicated in the staining procedures. The reagents used are listed in Supplementary Table 6.

### Statistical analysis

The obtained data were processed in Excel (Microsoft Corporation), and statistical analysis was performed using GraphPad Prism software 7.0c. The data are expressed as the mean values ± standard errors (SEM). One-way ANOVA, two-way ANOVA, and unpaired two-tailed t tests with Welch’s correction (heteroskedasticity distribution) were used to calculate the significance: a *p* value < 0.05 was considered to indicate statistical significance. The Shapiro‒Wilk test was used to calculate the normality distribution of the data before the test was performed. Following a significant two-way ANOVA, Fisher’s LSD test was used for post hoc pairwise comparisons between group means. In all figures, *p < 0.05, **p < 0.01, ***p < 0.001, and ***p < 0.0001.

### Data visualization

Heatmaps and dot plots were generated using the gplots (ver. 3.2.0) and DOSE (ver. 4.0.0) R packages.

## Supplementary information

Supplemental Material

Authorship form

## Data Availability

The transcriptomics data have been deposited in the NCBI Gene Expression Omnibus (GEO)^72^ and are accessible through GEO Series accession numbers GSE291017 with the title of the dataset “Accumulation of mutant Z Alpha1-Antitrypsin impairs intestinal function in mice,” and GSE291018 with the title “Transcriptomic analysis of human small intestinal organoids (hSIOs) from PiMM and PiZZ patients.” The mass spectrometry proteomic data have been deposited to the ProteomeXchange Consortium via the PRIDE partner repository with the dataset identifier PXD065749. The metagenomics data generated in this study have been deposited in the SRA database under BioProject ID PRJNA1290398.
