# Supplementary material for: Polymeric alpha-1 antitrypsin perturbs Paneth cell proteostasis and exacerbates intestinal inflammation
Source: Signal Transduct Target Ther. 2026 Sep 29;11:414. doi: 10.1038/s41392-026-02925-9 (PMC13620129; doi:10.1038/s41392-026-02925-9)
Supplement: Supplementary file 1 [file 41392_2026_2925_MOESM2_ESM.pdf]

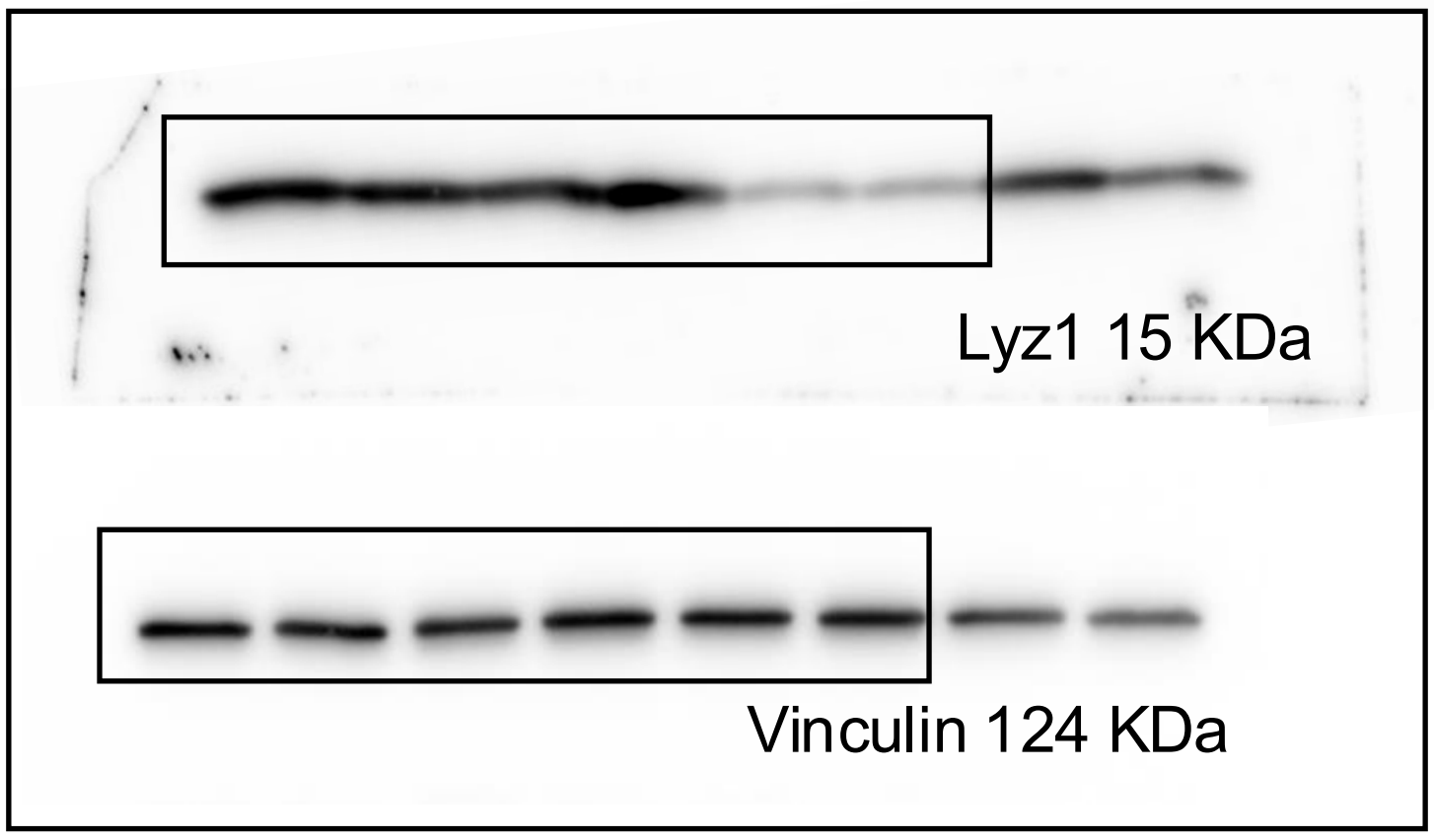
Lyz1 15 KDa
Vinculin 124 KDa

Figure 5C

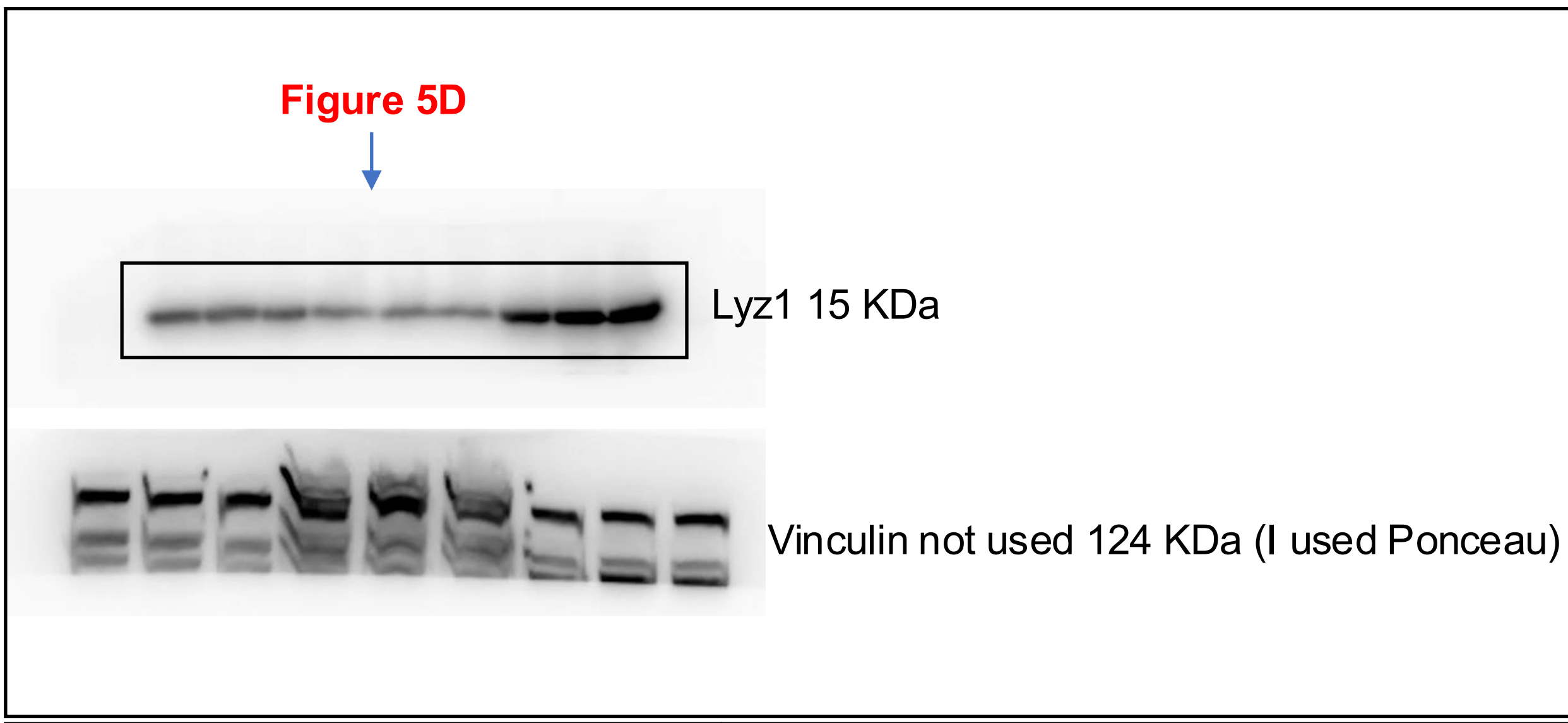
Figure 5D
Lyz1 15 KDa
Vinculin not used 124 KDa (I used Ponceau)

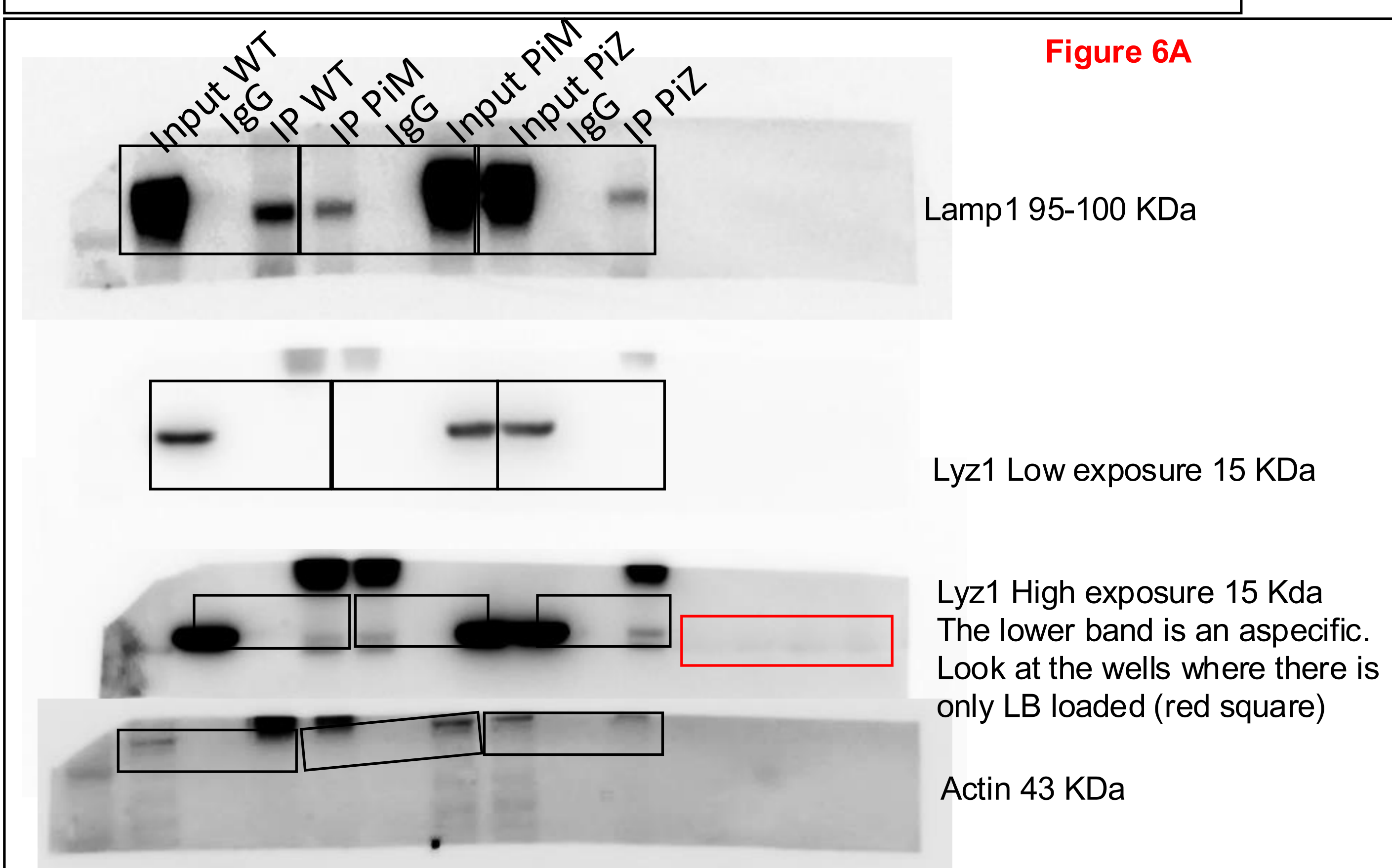
Figure 6A
Input WT
IgG
IP WT
IP PiM
IgG
Input PiM
Input PiZ
IgG
IP PiZ
Lamp1 95-100 KDa
Lyz1 Low exposure 15 KDa
Lyz1 High exposure 15 Kda
The lower band is an aspecific.
Look at the wells where there is
only LB loaded (red square)
Actin 43 KDa

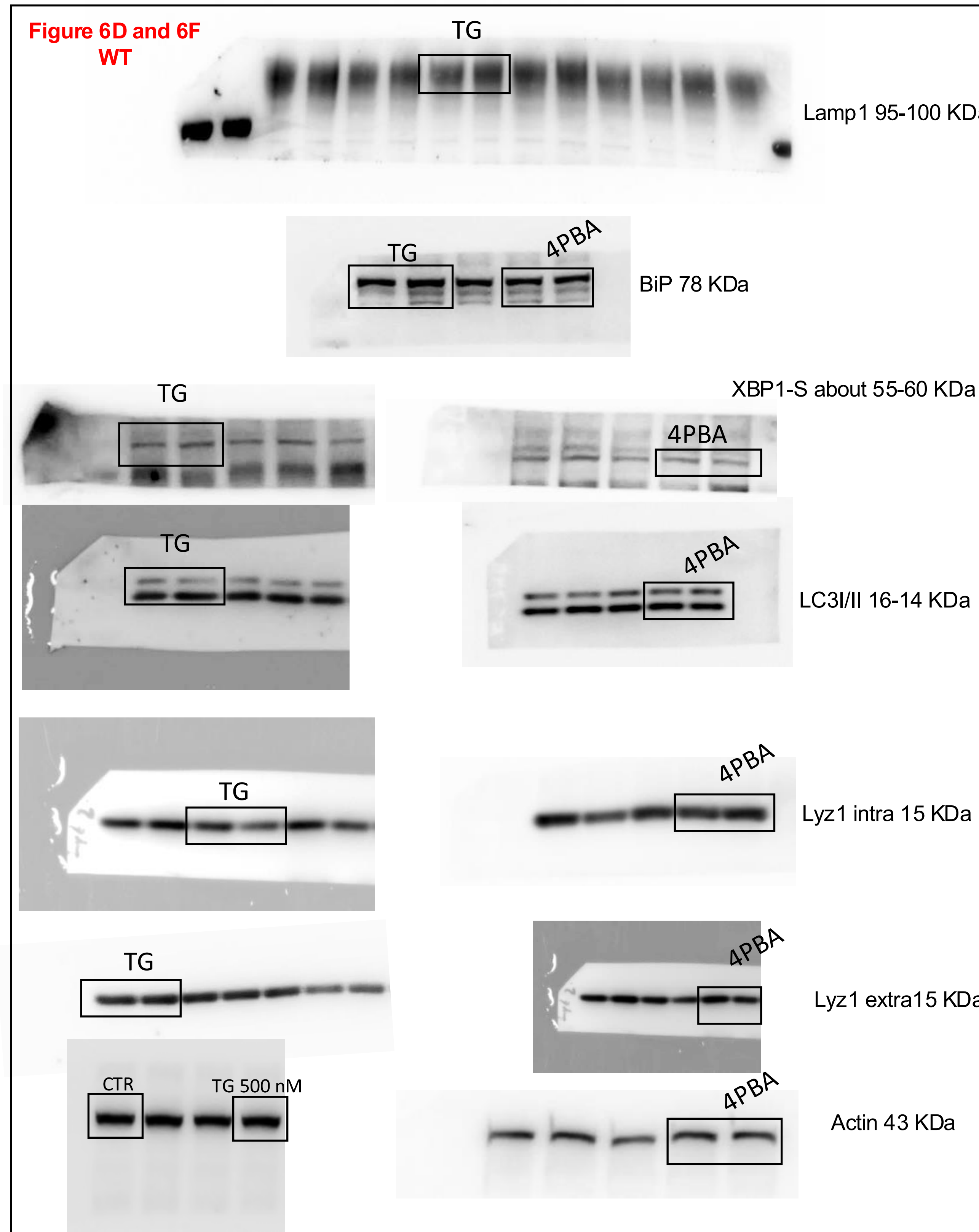
Figure 6D and 6F
WT
TG
Lamp1 95-100 KDa
TG
4PBA
BiP 78 KDa
TG
XBP1-S about 55-60 KDa
4PBA
TG
4PBA
LC3I/II 16-14 KDa
TG
4PBA
Lyz1 intra 15 KDa
TG
4PBA
Lyz1 extra15 KDa
CTR
TG 500 nM
4PBA
Actin 43 KDa

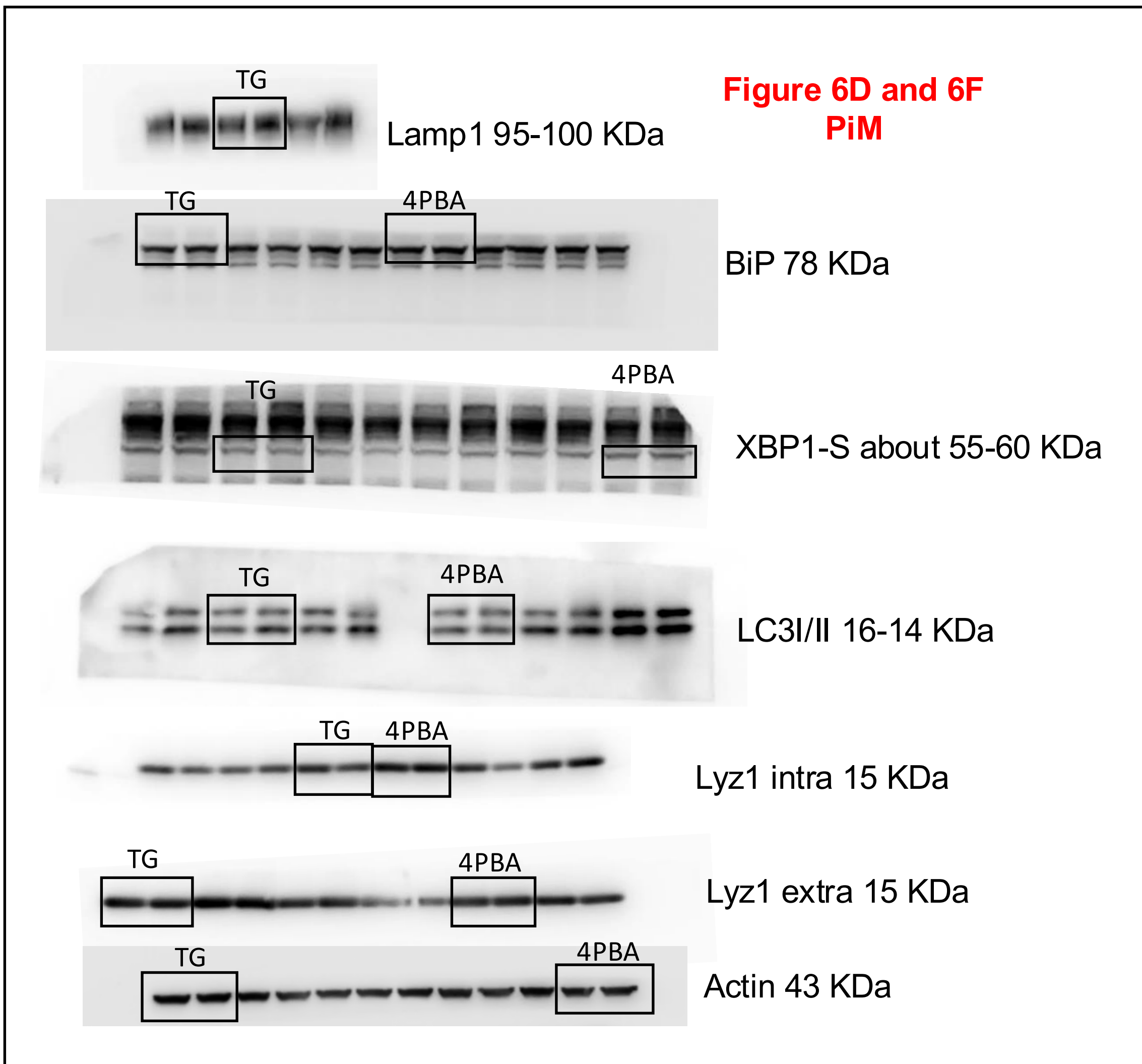
Figure 6D and 6F
PiM
TG
Lamp1 95-100 KDa
TG
4PBA
BiP 78 KDa
TG
4PBA
XBP1-S about 55-60 KDa
TG
4PBA
LC3I/II 16-14 KDa
TG
4PBA
Lyz1 intra 15 KDa
TG
4PBA
Lyz1 extra 15 KDa
TG
4PBA
Actin 43 KDa

Figure 6D and 6F
PiZ

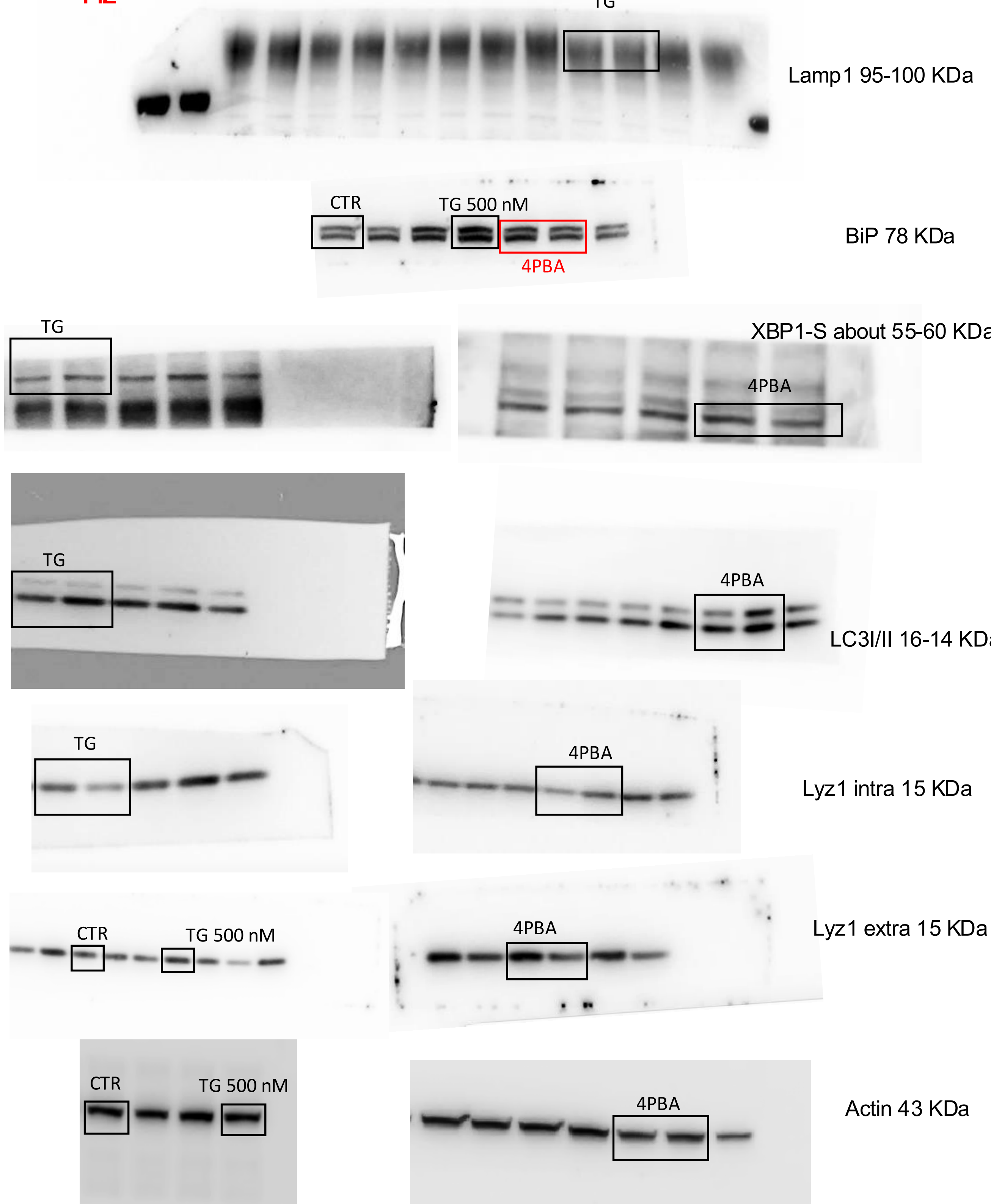

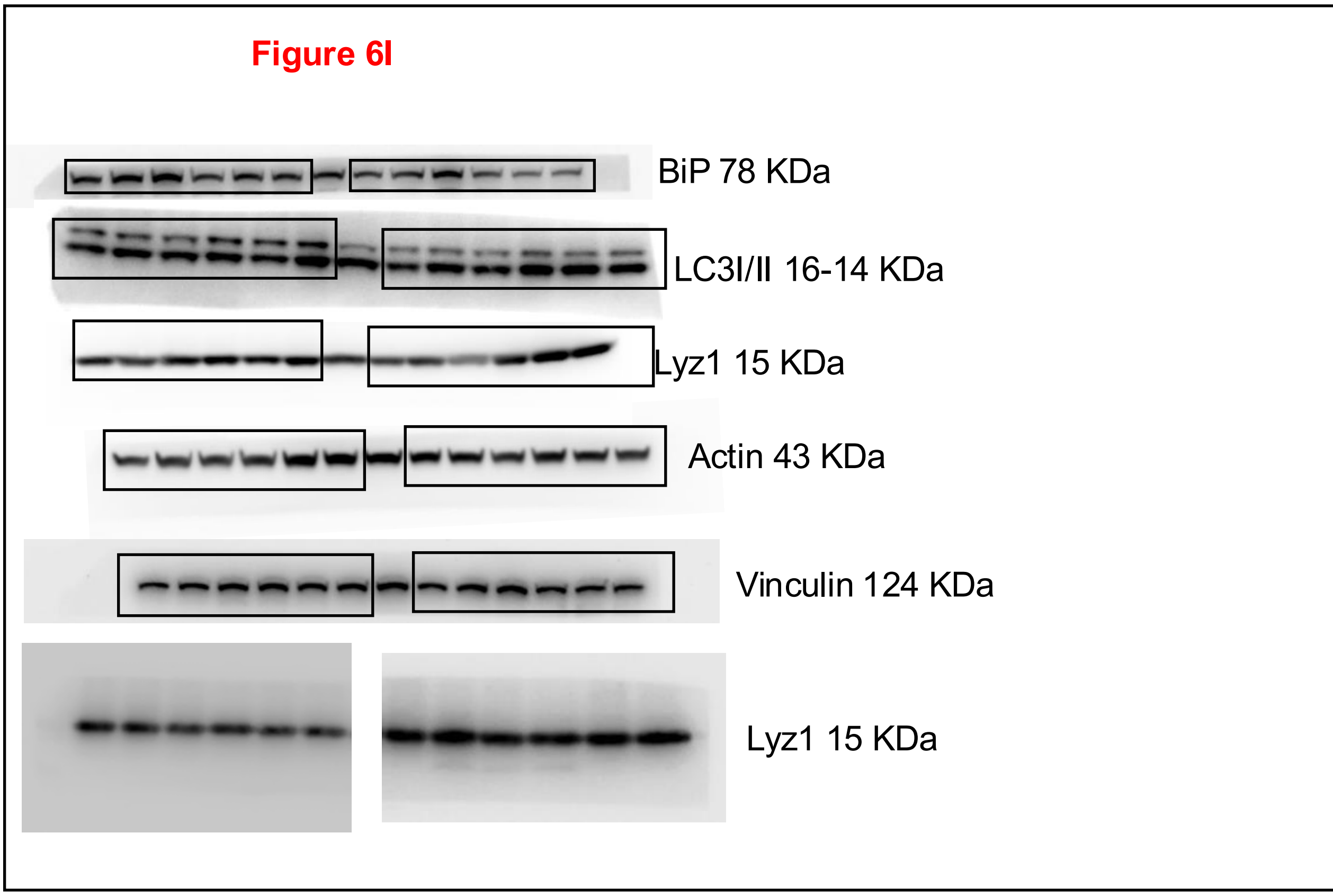
Figure 6I
BiP 78 KDa
LC3I/II 16-14 KDa
Lyz1 15 KDa
Actin 43 KDa
Vinculin 124 KDa
Lyz1 15 KDa

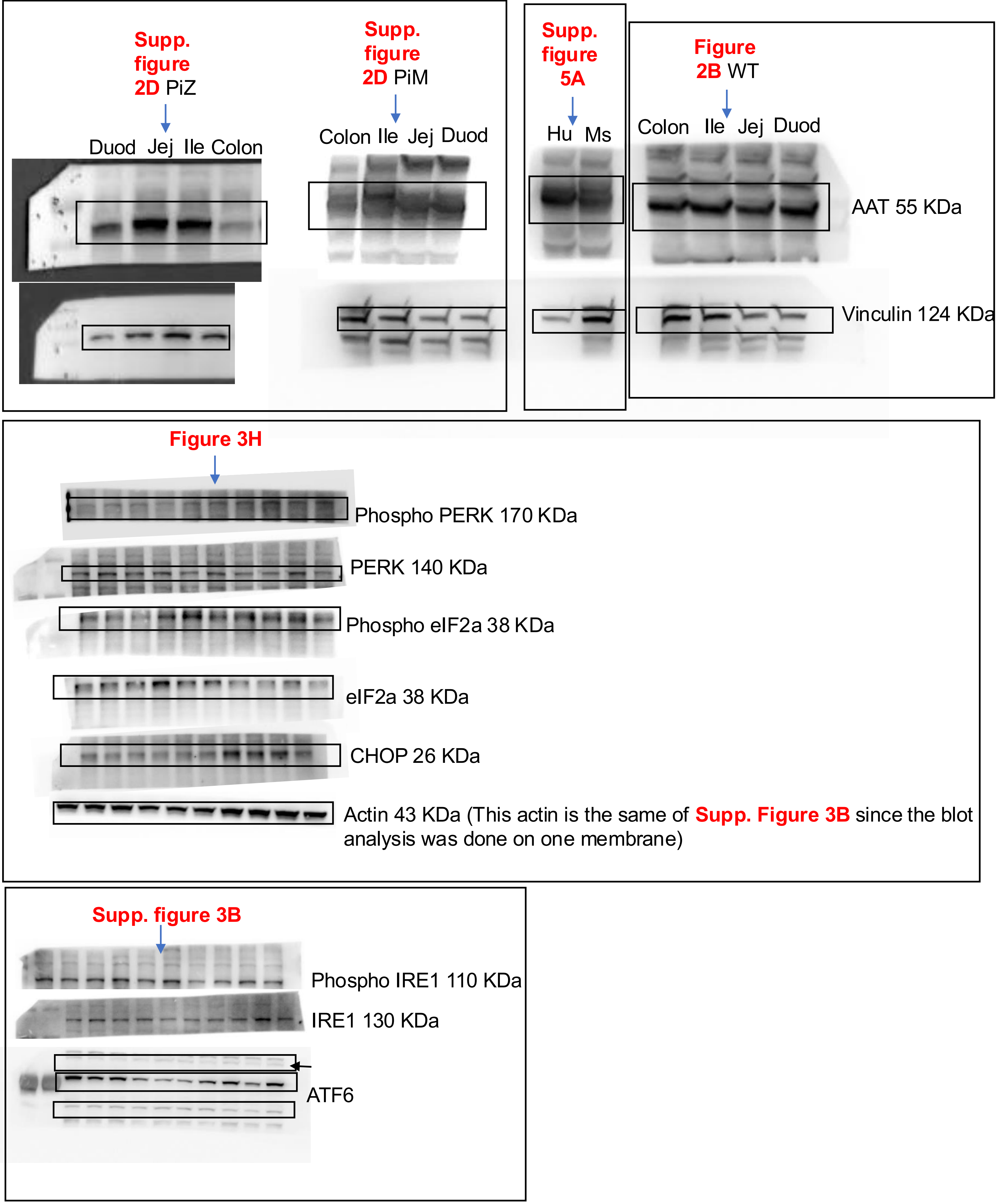
Supp. figure 2D PiZ
Duod Jej Ile Colon
Supp. figure 2D PiM
Colon Ile Jej Duod
Supp. figure 5A
Hu Ms
Figure 2B WT
Colon Ile Jej Duod
AAT 55 KDa
Vinculin 124 KDa
Figure 3H
Phospho PERK 170 KDa
PERK 140 KDa
Phospho eIF2a 38 KDa
eIF2a 38 KDa
CHOP 26 KDa
Actin 43 KDa (This actin is the same of Supp. Figure 3B since the blot analysis was done on one membrane)
Supp. figure 3B
Phospho IRE1 110 KDa
IRE1 130 KDa
ATF6

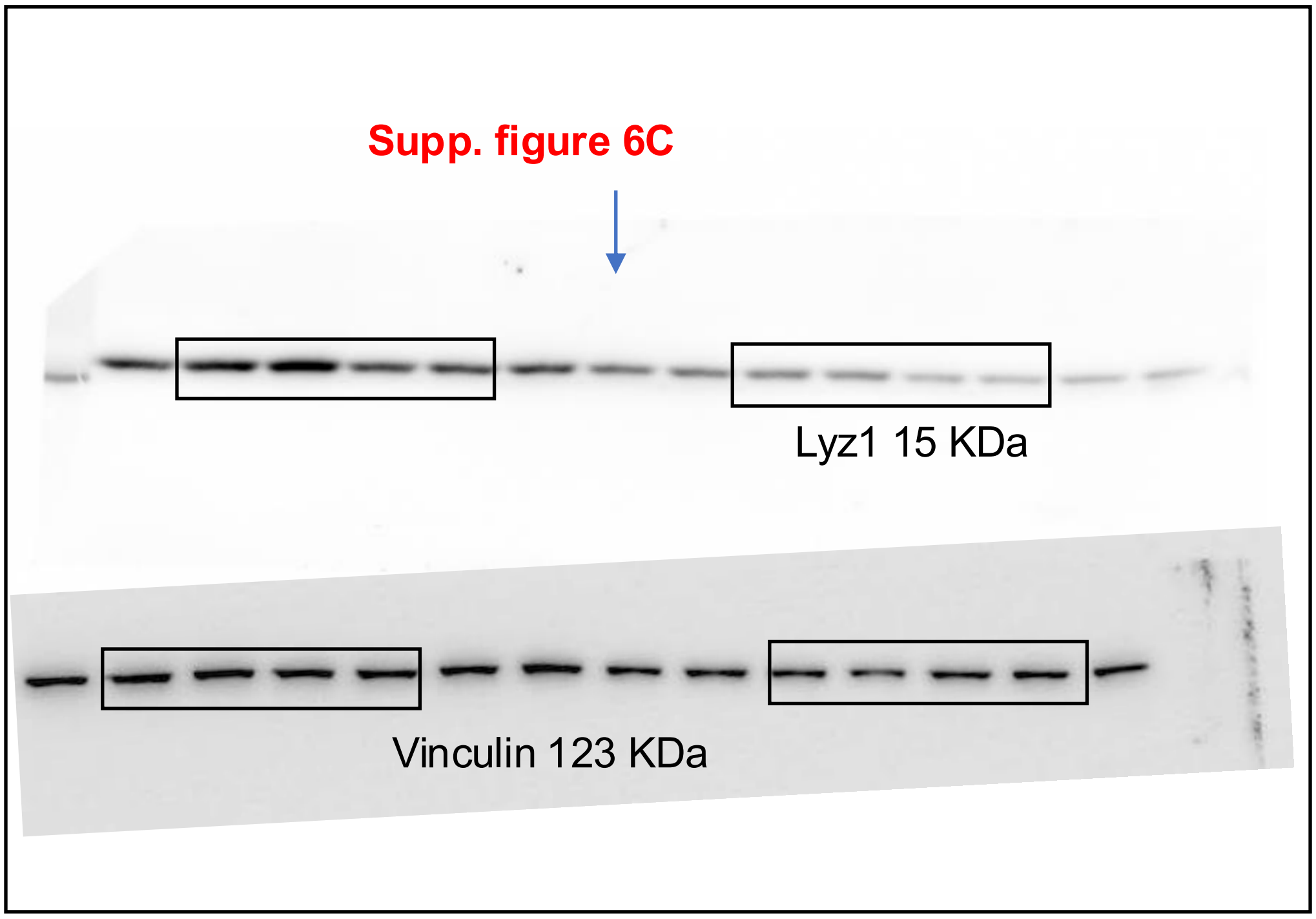
Supp. figure 6C
Lyz1 15 KDa
Vinculin 123 KDa

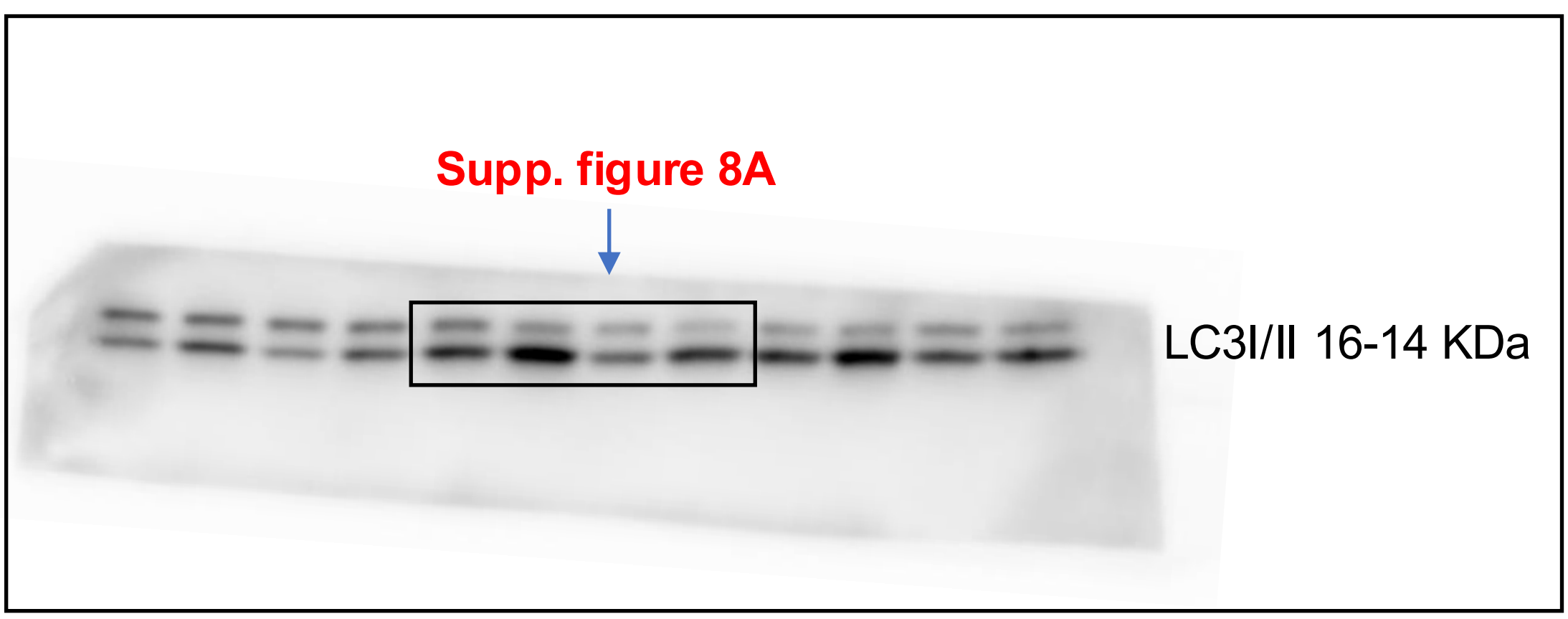
Supp. figure 8A
LC3I/II 16-14 KDa

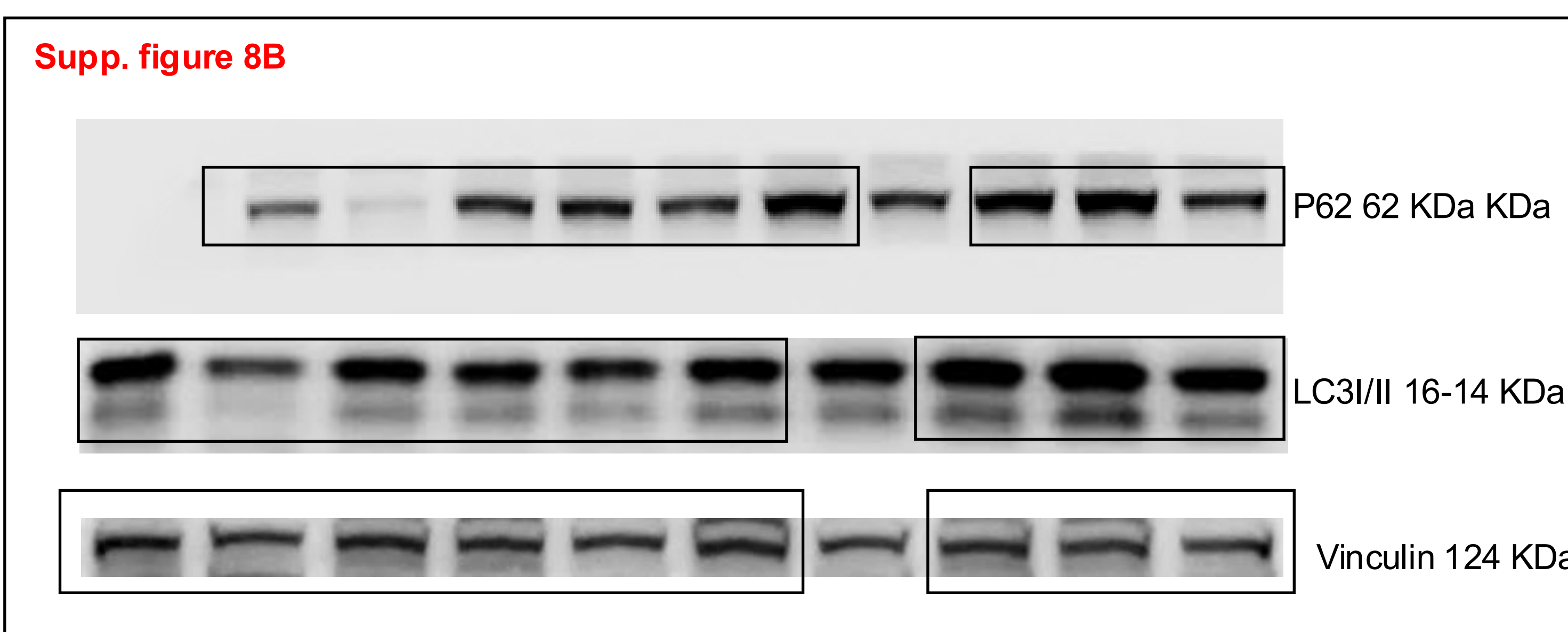
Supp. figure 8B
P62 62 KDa KDa
LC3I/II 16-14 KDa
Vinculin 124 KDa
