# Supplementary material for: Polymeric alpha-1 antitrypsin perturbs Paneth cell proteostasis and exacerbates intestinal inflammation
Source: Signal Transduct Target Ther. 2026 Sep 29;11:414. doi: 10.1038/s41392-026-02925-9 (PMC13620129; doi:10.1038/s41392-026-02925-9)
Supplement: Supplementary file 2 [file 41392_2026_2925_MOESM1_ESM.docx]

Supplementary Materials for

**Polymeric alpha-1 antitrypsin perturbs Paneth cell proteostasis and exacerbates intestinal inflammation**

Francesco Annunziata*, Felipe Dos Santos Matos, Irene Relvini, Jing Lu, Giovanna Federico, Claudia D’Agostino, Valentina Schiano, Veronica Maffia, Bruno Maria Custode, Gaetano Raiola, Barbara De Marino, Rossella De Cegli, Eugenio Del Prete, Elena Polishchuk, Carmen Ambrosio, Debora Compare, Gerardo Nardone, Francesco Neri, and Nunzia Pastore*

**Correspondence to:** [pastore@tigem.it](mailto:pastore@tigem.it)

**Joint corresponding author:** [f.annunziata@tigem.it](mailto:f.annunziata@tigem.it)

**This PDF file includes:**

Supplementary Figures S1-S8

Supplementary Tables S1-S9

**Figure. S1.**

**Supplementary Figure 1:** **Characterization of DSS-induced intestinal and hepatic changes in WT, PiM, and PiZ mice**

**a.** Small intestinal length quantification of the indicated genotypes. **b.** Representative immunofluorescence images of Lyz1 staining in ileum section of mice of the indicated genotypes. White asterisks indicate macrophages. White arrows indicate ectopic Lyz1 positive cells in the villi. **c.** *Tnf*α *and Il1*β mRNA levels normalized to *S16* transcript in livers from treated and untreated WT, PiM, and PiZ mice. **d.** Hydroxyproline levels in liver samples from mice of the indicated genotypes treated with DSS or vehicle. **e.** ALT and AST levels of WT, PiM, and PiZ control or DSS-treated. Each dot represents an individual mouse. All data refer to male mice. Data are presented as mean ± standard error. Data normality was assessed using the Shapiro-Wilk test. Statistical significance was calculated using two-way ANOVA: ns not significant **p*<0.05, ***p*<0.01, ****p*<0.001, and *****p*<0.0001.

**Figure. S2.**

**Supplementary Figure 2: Regional expression of *Serpina1* gene family members along the intestinal tract of young mice**

**a.** Average expression levels of *Serpina1a–Serpina1f* were calculated from epithelial single-cell RNA-seq data across five intestinal compartments: Duodenum (Duo), Jejunum (Jej), Ileum (Ile), Proximal Colon (Prox Col), and Distal Colon (Dist Col). Each boxplot shows the distribution of average expression across samples within each compartment. **b,c.** Cell-type-specific expression of *Serpina1b* across intestinal regions. Violin plots show the distribution of *Serpina1b* expression levels across distinct epithelial cell types within the duodenum (**b**) and jejunum (**c**) based on single-cell RNA sequencing. **d.** Representative immunoblot analysis for hAAT in PiM and PiZ Duodenum, Jejunum, Ileum, and Colon.

Each dot represents an individual mouse. Statistical significance was assessed using one-way ANOVA: **p*<0.05, ***p*<0.01, ****p*<0.001, and *****p*<0.0001.

**Figure. S3.**

**Supplementary Figure 3: ER Stress Pathway Activation in PiZ Intestinal Crypts**

**a.** KEGG pathway enrichment analysis of proteins differentially represented in the proteome of PiZ mice compared with WT controls (FDR < 0.05). **b.** Immunoblot analysis on IRE1α and ATF6 α UPR branches, in WT, PiM, and PiZ crypts. The actin blot is the same as that shown in Figure 3H because the proteins were analyzed on the same immunoblot. Each dot represents an individual mouse. Statistical significance was assessed using one-way ANOVA: ns not significant, **p*<0.05, ***p*<0.01, ****p*<0.001, and *****p*<0.0001.

**Figure. S4.**

**Supplementary Figure 4: Altered Intestinal Architecture in old PiZ mice**

**a-d.** Representative H&E images of duodenal sections from WT and PiZ mice at 80 weeks of age and quantification of average crypt length and villus length. **e-h.** Representative images of duodenal sections stained for Alcian blue (Goblet cells) (E-F), and Lyz1 (PCs) (G-H) in WT, and PiZ mice plus relative quantification.

**i.** Cladogram showing relative bacteria abundance in WT and PiZ mice. **j.** Relative *phyla* abundance in WT and PiZ mice.

Each dot represents an individual mouse. All data refer to male mice. Data are presented as mean ± standard error. Data normality was assessed using the Shapiro-Wilk test. Statistical significance was calculated using unpaired two-tail *t*-test with Welch’s corrections. *ns* not significant, **p*<0.05, ***p*<0.01, ****p*<0.001, and *****p*<0.0001.

**Figure. S5.**

**Supplementary Figure 5: AAT is expressed in human tissues and Small Intestinal Organoids (hSIOs).**

**a,b.** Immunoblot and immunofluorescence analysis for human AAT in colonic tissue from a control patient. **c.** Schematic representation of the differentiation protocol used to generate small intestinal organoids (SIO) from human iPSCs, through definitive endoderm and hindgut/midgut intermediates. **d.** Representative brightfield images of Pi*MM and Pi*ZZ hSIOs.

**Figure. S6.**

**Supplementary Figure 6: Aberrant Lysozyme secretion in PiZ Intestinal Crypts**

**a.** Transmission electron microscopy of ileal crypts showing clear differences in goblet cell (G) morphology between WT and PiZ mice. L=lumen. **b.** Intestinal permeability was assessed by measuring FITC-dextran in the serum after 4 hours post-oral gavage of FITC-dextran.

**c-d.** Immunoblot for Lyz1 on intracellular protein fraction of *ex-vivo* WT and PiZ small intestinal crypts incubated in the absence or presence of carbachol (10 μM) for 10 minutes at RT plus relative quantification. Vinculin was used as loading control.

Each dot represents an individual mouse. All data refer to male mice. Data are presented as mean ± standard error. Data normality was assessed using the Shapiro-Wilk test. Statistical significance was calculated using unpaired two-tail *t*-test with Welch’s corrections (Figure 1B), two-way ANOVA (Figure 3D), or one-way ANOVA (Figure 3E and 3F). ns not significant, **p*<0.05, ***p*<0.01, ****p*<0.001, and *****p*<0.0001.

**Figure. S7.**

**Supplementary Figure 7: Oral Lysozyme Supplementation exacerbates DSS-Induced Intestinal and Liver Damage in PiZ Mice**

**a.** Schematic representation of the experimental design for DSS and lysozyme experiment in 8-week-old PiZ mice. Mice received 3% DSS in drinking water for 7 days. DSS-treated-lysozyme supplemented PiZ were given daily oral administration of recombinant human lysozyme (20 mg/Kg). **b.** Body weight changes in DSS-treated PiZ and DSS-treated-lysozyme supplemented PiZ mice over the course of the experiment. **c-e**. Gross morphology of colons of DSS-treated PiZ and DSS-treated-lysozyme supplemented PiZ and quantification of colon and small intestinal length. **f.** Representative images of H&E-stained colon sections from mice of the indicated genotypes and relative histopathological scores. **g.** Representative images of H&E-, Sirius Red-, and PAS-D- stained liver sections from DSS-treated PiZ and DSS-treated-lysozyme supplemented PiZ and relative quantification.

Each dot represents an individual mouse. All data refer to male mice. Data are presented as mean ± standard error. Data normality was assessed using the Shapiro-Wilk test. Statistical significance was calculated using unpaired two-tail *t*-test with Welch’s corrections: *ns* not significant, **p*<0.05, ***p*<0.01, ****p*<0.001, and *****p*<0.0001.

**Figure. S8.**

**Supplementary Figure 8: a.** Immunoblot for LC3 on intracellular protein fraction of ex-vivo WT and PiZ small intestinal crypts incubated in the absence or presence of chloroquine (100 μM) for 45 minutes and relative quantification. Vinculin was used as loading control. **b.** Immunoblot analysis for autophagy markers SQSTM1/p62 and LC3 on ileum lysates from WT, PiM, and PiZ mice. **c.** qPCR analysis for Lysozyme expression in crypts from WT, PiM, and PiZ mice treated with DMSO or 4-PBA showing no effect on Lyz1 mRNA levels. Data are presented as mean ± standard error. Data normality was assessed using the Shapiro-Wilk test. Statistical significance was calculated using two-way ANOVA: *ns* not significant, **p*<0.05, ***p*<0.01, ****p*<0.001.

**Table S1.** *GOEA performed on 107 upregulated genes (FDR 10%) in PiZ small intestinal crypts compared to WT, restricting the output to KEGG pathway terms.*

| **Category** | **Term** | **Count** | **Genes** | **FDR** |
| --- | --- | --- | --- | --- |
| KEGG_PATHWAY | mmu04141:Protein processing in endoplasmic reticulum | 15 | PDIA3, SEC13, HSPA5, EDEM2, RPN1, MOGS, PDIA6, CKAP4, DDOST, SEC61A1, GANAB, DNAJB11, SSR1, UGGT1, SEC24C | 3.22E-09 |
| KEGG_PATHWAY | mmu03050:Proteasome | 6 | PSMB2, PSMA2, PSMD3, PSME2, PSMB8, PSMB9 | 0.0017691 |
| KEGG_PATHWAY | mmu04612:Antigen processing and presentation | 6 | PDIA3, HSPA5, H2-DMB1, H2-Q7, PSME2, TAP1 | 0.02275986 |
| KEGG_PATHWAY | mmu05012:Parkinson disease | 9 | TUBA1B, HSPA5, PSMB2, PSMA2, PSMD3, VDAC2, SLC25A5, COX5A, TUBA4A | 0.02275986 |
| KEGG_PATHWAY | mmu05020:Prion disease | 9 | TUBA1B, HSPA5, PSMB2, PSMA2, PSMD3, VDAC2, SLC25A5, COX5A, TUBA4A | 0.02275986 |
| KEGG_PATHWAY | mmu05014:Amyotrophic lateral sclerosis | 10 | GPX2, SEC13, TUBA1B, HSPA5, PSMB2, FUS, PSMA2, PSMD3, COX5A, TUBA4A | 0.03520063 |
| KEGG_PATHWAY | mmu05016:Huntington disease | 9 | GPX2, TUBA1B, PSMB2, PSMA2, PSMD3, VDAC2, SLC25A5, COX5A, TUBA4A | 0.03520063 |
| KEGG_PATHWAY | mmu05022:Pathways of neurodegeneration - multiple diseases | 11 | GPX2, TUBA1B, HSPA5, PSMB2, FUS, PSMA2, PSMD3, VDAC2, SLC25A5, COX5A, TUBA4A | 0.03723768 |
| KEGG_PATHWAY | mmu04918:Thyroid hormone synthesis | 5 | GPX2, HSPA5, DUOXA2, ATP1A1, DUOX2 | 0.03723768 |

**Table S2.** *GOEA restricted to KEGG pathway analysis on 524 induced proteins enriched in PiZ crypts compared to WT (FDR 5%).*

| **Category** | **Term** | **Count** | **Genes** | **FDR** |
| --- | --- | --- | --- | --- |
| KEGG_PATHWAY | mmu03010:Ribosome | 30 | RPL3, RPL32, MRPL18, RPL10A, RPL6, MRPL11, RPS4X, RPS14, MRPL20, RPS15A, RPS16, RPS18, RPL13, RPL35, RPL38, RPL18, RPS9, RPS7, RPS8, RPS6, RPL13A, MRPS21, RPS26, RPS25, RPL27A, RPL37A, RPL27, RPL26, RPL28, RPS23 | 1.31E-06 |
| KEGG_PATHWAY | mmu00970:Aminoacyl-tRNA biosynthesis | 12 | GARS1, HARS1, QARS1, LARS1, MARS1, GATC, TARS2, CARS1, FARSA, YARS2, DARS1, VARS1 | 6.67E-04 |
| KEGG_PATHWAY | mmu05171:Coronavirus disease - COVID-19 | 28 | RPL3, RPL32, RPL10A, RPL6, RPS4X, RPS14, RPS15A, RPS16, RPS18, RPL13, RPL35, RPL38, RPL18, RPS9, RPS7, RPS8, RPS6, RPL13A, RPS26, RPS25, TRAF6, RPL27A, RPL37A, RPL27, RPL26, RPL28, RPS23, RIGI | 6.67E-04 |
| KEGG_PATHWAY | mmu03040:Spliceosome | 22 | PRPF38B, RBM25, PRPF38A, SF3B3, DDX46, HSPA1L, CRNKL1, EFTUD2, SNRNP40, PHF5A, SNRPD1, PRPF3, PCBP1, TRA2A, SRSF2, SRSF3, SRSF4, PRPF31, SNRNP200, SRSF6, SRSF7, SRSF10 | 0.00723378 |
| KEGG_PATHWAY | mmu04141:Protein processing in endoplasmic reticulum | 17 | HSP90AA1, SEC24A, HSP90AB1, HSPA1L, PRKCSH, HSPA4L, RPN1, MOGS, MAGT1, EIF2S1, HSP90B1, DNAJC3, ERLEC1, HSPH1, BAX, UBXN4, SEC62 | 0.00772462 |
| KEGG_PATHWAY | mmu03013:Nucleocytoplasmic transport | 13 | NDC1, UPF1, AAAS, SRRM1, EEF1A1, NUP93, DDX19A, SUMO1, NUP88, KPNA2, TNPO1, KPNB1, KPNA1 | 0.01688793 |

**Table S3.** *GOEA performed on 577 induced and 75 inhibited genes (FDR 5%) in Pi*ZZ compared to Pi*MM hSIO, separately restricting the output to KEGG pathways for induced (A) and inhibited (B) genes.*

**A) INDUCED**

| **Category** | **Term** | **Count** | **Genes** | **FDR** |
| --- | --- | --- | --- | --- |
| KEGG_PATHWAY | hsa01100:Metabolic pathways | 352 | CMAS, PANK3, XYLT1, ENO1, ENO2, GLS, NSDHL, PNP, LIPC, SCP2, TDO2, ANPEP, LIPG, NAMPT, CKMT1B, PLCE1, PIP4K2C, ATP6V1E1, XYLB, ARSA, GSTK1, ST6GAL1, ATP6V0E1, ACSL1, ENTPD5, ENTPD6, TALDO1, ENTPD8, ACSL5, CMBL, ACSL3, ASRGL1, CYP27A1, MTHFD2, ETHE1, UQCRC1, SUCLG2, SUCLG1, UQCRC2, ATP6V0D1, GNE, PTGES, CDS1, MGST3, RPN1, MGST1, MGST2, GNS, ABO, UGCG, UGT8, SGPL1, LDHA, CYP2B6, INPP5A, PPCS, RDH10, SI, AFMID, HMOX1, PIP5K1B, ASL, RDH13, PCK1, B4GALNT2, ATP6V1C1, HMOX2, ATP6V0A1, PCK2, GSTM4, ALOX15B, PAFAH2, DERA, EXT1, CYP2C9, SQLE, BST1, GLUD1, CYP2C8, GLUD2, VNN1, AKR1B10, ADI1, CYP2S1, OGDH, ALDH18A1, GPI, COX7B, ACY1, MOGAT3, MOGAT2, HEXB, HEXA, PYGL, AACS, SMPD3, GYS2, GYS1, SMPD2, IMPA2, GUK1, RFK, SMPD1, IDS, DLAT, ACADS, COX8A, ELOVL1, ATP6V0B, PCYT1A, TPI1, GSTO1, ITPK1, AKR1A1, NME3, SIRT6, SIRT7, UGDH, ITPKC, HADHA, PLCB3, BDH1, B3GNT5, ITPKA, DPYD, B3GNT3, RGN, ALDOC, ALDOB, ALDOA, MGLL, GUCY2C, ASAH1, ASAH2, OAT, GUCY2D, INPP1, GSTP1, AKR1D1, GCNT1, DOT1L, GDPD3, NPL, SGMS2, COX5B, COX5A, PAPSS2, TYMP, HSD17B2, GCNT2, GCNT3, SC5D, GPAT3, CKB, XDH, CA12, NQO1, AOC1, GK, MDH2, GLYCTK, AKR1C1, CKMT1A, AKR1C3, AKR1C2, NMNAT2, COX6C, FMO5, DHRS3, AGMAT, GATM, CYP2W1, GPAM, DHRS9, ETNK1, GSTA2, GSTA1, PNPLA3, PLCD3, OTC, ALDH1L1, ACAA2, GDA, DGKA, NDUFA10, MPI, MSMO1, PLOD2, SAT2, HNMT, COMT, COX6A1, SAT1, GALNT10, CNDP2, ACAA1, GGT1, HADH, ENPP7, DGAT2, PCYT2, HGD, DGAT1, IPMK, UGT2B17, SDHD, PGD, SDHA, SDHB, COX6B1, ALDH3A2, PLA2G10, CMPK1, CMPK2, PFKFB2, IDI1, PFKFB4, PFKFB3, ALOX15, AK2, AK4, COX7A2, CYP2C19, PLD1, FUT2, CYP2C18, AK8, FUT4, MTM1, EBP, FUT8, ALDH3B1, AMDHD2, UAP1L1, PGK1, HYI, CYC1, NDUFV1, ATP6V1F, MPST, FDPS, GOT1, EPHX2, IDH1, GFPT1, SRD5A3, DHCR24, B3GALT5, SYNJ2, CRLS1, CPS1, P4HA1, NAT2, P4HA2, PI4KA, CRYL1, CYCS, ACO1, ALPI, ACO2, DHCR7, GALK1, ST6GALNAC1, ACADVL, B4GALT1, ALAS1, COX4I1, AMD1, TCIRG1, HK1, ALDH2, CA2, KYNU, ENOSF1, NAPRT, CA4, ME1, ACP5, UQCRFS1, MAN1A1, ME3, CA9, HMGCS2, PGLS, GUSB, COLGALT1, IDH3A, GPX2, PTGIS, PLA2G12B, ATP6AP1, HMGCS1, GPX4, GPX3, PDE4D, URAD, LSS, NT5C3A, PKM, NDUFS7, TST, ACOX1, HKDC1, ALDH1A1, NDUFS2, BLVRB, MVD, HAGH, AGXT, LCT, GAPDH, ST6GALNAC4, B4GALT5, CERS2, DDC, PDXK, ACSS2, CANT1, MVK, HMGCR, CYP3A4, CYP3A5, AGPAT2, AGPAT3, HMGCL, UGP2, GMDS, ACSS1, NDUFA9, NDUFA8, GALNT5, FAHD1, GALNT3, HSD3B2, SMOX, HSD3B1, ASS1, GALNS, GALE, PFKL, MGAM2, GNPDA1, PC, IMPDH1, SCD, MGAT4A, MGAT4B, SARDH, GALM, CHPT1, PFKP, LPIN3 | 1.07E-17 |
| KEGG_PATHWAY | hsa04142:Lysosome | 51 | SCARB2, HEXB, HEXA, CLTC, CTSZ, CLTB, CLTA, TCIRG1, CTSV, NAGPA, LIPA, LITAF, CTSS, LAPTM4A, GM2A, LAMP1, CTSL, SMPD1, AP1S1, ACP5, IDS, CTSH, GUSB, CTSE, CTSD, CTSC, CTSB, ARSA, ATP6V0B, ATP6AP1, AP1B1, NPC1, NPC2, ATP6V0D1, CD63, ASAH1, GNS, CLN3, PSAP, SLC17A5, ATP6V0A1, CTSA, CD164, PSAPL1, FUCA1, FUCA2, CTNS, GALNS, TPP1, CD68, LGMN | 7.58E-09 |
| KEGG_PATHWAY | hsa05130:Pathogenic Escherichia coli infection | 66 | ARF1, WIPF1, TRADD, ARPC1B, CLDN1, ACTB, ACTG1, PYCARD, TUBA1C, TUBB6, CASP7, BAIAP2L1, CASP8, TUBB3, CASP3, CASP4, CLDN23, CASP1, NCK2, RAC1, ACTR3, ACTR2, F2R, IL18, FOS, IRAK4, F2, RHOA, TNFRSF1A, CLDN4, TUBB2A, CTTN, CLDN7, CLDN18, TMBIM6, EZR, GAPDH, TLR4, ARF6, RAB1A, RELA, PAK1, MYO6, MYH14, WASF2, CYTH1, MAPK3, JUN, SEC24A, TNFRSF10A, ARPC4, ARPC5, TUBB4B, MAPK13, MYO1D, NFKBIA, MYO1E, MYO1C, ARPC2, MYO1A, ARPC3, CYCS, TAB3, SEC24D, SEC24C, MYD88 | 1.06E-08 |
| KEGG_PATHWAY | hsa03320:PPAR signaling pathway | 34 | ILK, DBI, RXRA, SCP2, UBC, ME1, ME3, HMGCS2, ACAA1, PCK1, PLTP, PCK2, CPT1A, HMGCS1, GK, ACSL1, MMP1, PDPK1, NR1H3, APOA1, APOC3, ACSL5, ACSL3, FABP1, CYP27A1, FABP2, FABP3, SCD, ACOX1, FABP6, PLIN2, PPARA, SLC27A2, PPARD | 8.03E-08 |
| KEGG_PATHWAY | hsa05132:Salmonella infection | 74 | ARF1, TRADD, ARPC1B, ACTB, ACTG1, PYCARD, TUBA1C, TUBB6, CASP7, CASP8, TUBB3, CASP3, KIF5B, CASP4, CASP1, RAC1, MAP2K3, ACTR3, ACTR2, MAP2K2, RIPK3, ANXA2, RHOG, IL18, FOS, IRAK4, RHOA, TNFRSF1A, DNM2, TUBB2A, GAPDH, TLR4, ARL8A, ARL8B, RAB7A, ARF6, RALA, RAB5C, DCTN2, AHNAK, SNX33, MYL12A, RELA, HSP90B1, MYL12B, KLC4, PAK1, RRAS, PODXL, MYO6, FLNB, SNX9, RILP, CYTH1, S100A10, MAPK3, GSDMD, DYNC1H1, JUN, MLKL, AHNAK2, TNFRSF10A, ARPC4, ARPC5, TUBB4B, MAPK13, NFKBIA, ARPC2, ARPC3, PLEKHM1, CYCS, TAB3, PKN1, MYD88 | 1.47E-07 |
| KEGG_PATHWAY | hsa01200:Carbon metabolism | 43 | GPI, ACSS2, ENO1, ENO2, HK1, ME1, PGK1, ME3, PGLS, DLAT, ACSS1, ACADS, IDH3A, TPI1, GOT1, MDH2, IDH1, GLYCTK, TALDO1, SDHD, PGD, SDHA, SDHB, GLUD1, PFKL, GLUD2, PKM, PC, CPS1, ACOX1, HKDC1, RGN, OGDH, SUCLG2, SUCLG1, ALDOC, ACO1, ALDOB, ACO2, ALDOA, AGXT, GAPDH, PFKP | 3.12E-07 |
| KEGG_PATHWAY | hsa04144:Endocytosis | 73 | VPS29, ARF4, ARF1, TFRC, WIPF1, ARPC1B, CLTC, CLTB, CLTA, CBLC, ARRB1, CBLB, ARRB2, RAB22A, EEA1, CAPZB, KIF5B, VPS35, LDLRAP1, SH3GL1, SH3GLB1, ACTR3, SH3GLB2, ACTR2, PDCD6IP, VPS37C, ARAP3, HLA-C, HLA-A, VPS37B, ARFGAP3, RHOA, HLA-E, DNM2, EPN3, RAB31, BIN1, HGS, CHMP3, CHMP4B, VPS28, CHMP5, RAB7A, ARF6, RAB5C, VPS4B, NEDD4L, CXCR4, PLD1, IL2RG, PRKCZ, EGFR, SNX3, PARD6B, RAB11FIP1, PIP5K1B, LDLR, CYTH1, RAB11FIP4, SNX6, RAB11FIP5, ARFGEF1, SMAD3, ARPC4, ARPC5, RAB10, EHD1, EHD4, ARPC2, ARPC3, CAPZA2, CHMP2B, HSPA1A | 3.12E-07 |
| KEGG_PATHWAY | hsa04216:Ferroptosis | 23 | STEAP3, TFRC, GPX4, ACSL1, ALOX15, NCOA4, SLC40A1, ACSL5, SAT2, SLC3A2, ACSL3, SLC7A11, CP, SLC39A14, SAT1, TF, MAP1LC3B, PCBP1, LPCAT3, FTH1, VDAC2, HMOX1, FTL | 4.48E-07 |
| KEGG_PATHWAY | hsa04979:Cholesterol metabolism | 25 | SCARB1, LRP1, PCSK9, LIPA, LIPC, LIPG, TSPO, APOE, LDLRAP1, APOB, LDLR, PLTP, OSBPL5, CIDEB, APOA1, APOC3, APOA4, CYP27A1, NCEH1, NPC1, VAPA, NPC2, SOAT2, VDAC2, VDAC1 | 1.32E-06 |
| KEGG_PATHWAY | hsa05208:Chemical carcinogenesis - reactive oxygen species | 63 | COX7B, COX4I1, NDUFA10, AHR, COX6A1, CYP1B1, UQCRFS1, RAC1, MAP3K5, COX8A, MAP2K2, PDPK1, GSTO1, PRKCD, AKR1A1, FOS, SDHD, SDHA, SDHB, COX6B1, NDUFS7, UQCRC1, PPIF, NDUFS2, VDAC2, VDAC1, UQCRC2, SLC25A5, SLC25A6, MGST3, MGST1, MGST2, COX7A2, COX5B, PLD1, COX5A, EGFR, RELA, NRAS, HMOX1, CYC1, NDUFV1, MAPK3, GSTM4, NDUFA9, NQO1, NDUFA8, JUN, EPHX2, AKR1C1, AKR1C3, EPHX4, EPHX3, AKR1C2, COX6C, SOD2, MAPK13, VEGFA, NFKBIA, GSTA2, GSTA1, NOX1, SLC26A6 | 9.53E-06 |
| KEGG_PATHWAY | hsa05417:Lipid and atherosclerosis | 61 | ERO1A, CALML4, PYCARD, PPP3CA, CASP7, CASP8, CASP3, CASP1, LBP, RAC1, MAP3K5, VAV3, MAP2K3, PDPK1, MMP1, IL18, APOA1, APOA4, FOS, IRAK4, TICAM1, RHOA, TNFRSF1A, PLCB3, DDIT3, IRF7, TLR4, ATF4, CAMK2D, TANK, RELA, HSP90B1, RAP1B, NRAS, PPP3R1, RAP1A, CYP2B6, RXRA, APOB, BID, CAMK2G, LDLR, MAPK3, LYN, JUN, XBP1, HSPA5, STAT3, TNFRSF10A, SOD2, MAPK13, NFKBIA, CYP2C9, CYP2C8, CYCS, CALM3, CALM1, MYD88, NOX1, BCL2L1, HSPA1A | 1.16E-05 |
| KEGG_PATHWAY | hsa04978:Mineral absorption | 26 | SLC46A1, SLC40A1, ATP1A1, SLC5A1, MT2A, ATP7B, HEPH, FTH1, HMOX1, ATP7A, TRPM6, SLC39A4, HMOX2, SLC34A2, S100G, SLC31A1, VDR, ATP2B4, ATP1B3, SLC30A1, TF, MT1F, MT1B, SLC26A3, SLC26A6, FTL | 1.42E-05 |
| KEGG_PATHWAY | hsa04145:Phagosome | 46 | SCARB1, STX12, RAB5C, ITGB5, TFRC, C1R, TCIRG1, CTSS, ACTB, ACTG1, C3, EEA1, SEC61A1, TUBA1C, TUBB6, LAMP1, TUBB3, CTSL, RAC1, ATP6V1E1, RILP, ATP6V1C1, ATP6V1F, ATP6V0A1, MSR1, DYNC1H1, ATP6V0B, ATP6V0E1, ATP6AP1, ITGA2, STX7, TAP2, HLA-C, TAP1, HLA-A, TUBB4B, HLA-E, TUBB2A, HGS, CANX, CALR, ATP6V0D1, TLR4, HLA-DRB1, RAB7A, VAMP3 | 4.68E-05 |
| KEGG_PATHWAY | hsa05131:Shigellosis | 65 | ARF1, CALCOCO2, TRADD, ARPC1B, ILK, ACTB, HK1, ACTG1, PYCARD, MAP1LC3B, CAPNS1, CASP4, CAPN2, CASP1, PLCE1, CAPN1, RAC1, CAST, ACTR3, ACTR2, PRKCD, IL18, WIPI1, ACTN4, RHOA, TNFRSF1A, PLCB3, CTTN, RRAGC, HKDC1, VDAC1, TLN2, RBCK1, SQSTM1, TLR4, CD44, VCL, ARF6, PXN, ITPR3, BCL10, MYL12A, EGFR, RELA, MYL12B, C3, UBC, WASF2, CYTH1, MAPK3, JUN, BNIP3, ARPC4, ARPC5, MAPK13, NFKBIA, DIAPH1, TNIP1, ARPC2, ARPC3, CYCS, TAB3, PLCD3, MYD88, BCL2L1 | 6.37E-05 |
| KEGG_PATHWAY | hsa04066:HIF-1 signaling pathway | 36 | CDKN1A, CAMK2D, CDKN1B, PFKFB3, TFRC, SLC2A1, ENO1, ENO2, RELA, EGFR, HK1, LDHA, PGK1, HMOX1, TIMP1, CAMK2G, IL6R, MAPK3, EGLN1, EGLN3, EDN1, MAP2K2, IFNGR1, INSR, STAT3, VEGFA, PFKL, TF, HKDC1, ALDOC, LTBR, ALDOB, ALDOA, GAPDH, TLR4, PFKP | 6.79E-05 |
| KEGG_PATHWAY | hsa04932:Non-alcoholic fatty liver disease | 46 | CEBPA, COX7B, IRS1, COX4I1, NDUFA10, COX7A2, COX5B, COX6A1, COX5A, ADIPOR2, RELA, CASP7, CASP8, RXRA, CASP3, UQCRFS1, CYC1, RAC1, BID, IL6R, NDUFV1, MAP3K5, COX8A, NDUFA9, SREBF1, NDUFA8, XBP1, JUN, INSR, NR1H3, FOS, SDHD, COX6C, SDHA, SDHB, TNFRSF1A, MAPK13, COX6B1, NDUFS7, DDIT3, UQCRC1, NDUFS2, CYCS, UQCRC2, PPARA, ATF4 | 6.79E-05 |
| KEGG_PATHWAY | hsa05135:Yersinia infection | 42 | WIPF1, ARPC1B, PXN, RELA, ACTB, ACTG1, PYCARD, RPS6KA3, RPS6KA1, CASP1, RAC2, PTK2B, PIP5K1B, RAC1, WASF2, MAPK3, VAV3, MAP2K3, ACTR3, ACTR2, JUN, MAP2K2, RHOG, IL18, FN1, ARPC4, ARPC5, FOS, IRAK4, TICAM1, RHOA, MAPK13, NFKBIA, CD8B, ARPC2, ARPC3, GNAQ, PKN1, TLR4, MYD88, SKAP2, ARF6 | 7.40E-05 |
| KEGG_PATHWAY | hsa05415:Diabetic cardiomyopathy | 55 | COX7B, IRS1, COX4I1, NDUFA10, ATP2A3, SLC2A1, ATP2A2, COX6A1, GYS2, GYS1, MPC1, MPC2, RAC2, PDK3, UQCRFS1, RAC1, CTSD, COX8A, PRKCD, SDHD, SDHA, SDHB, COX6B1, PLCB3, NDUFS7, UQCRC1, PPIF, NDUFS2, VDAC2, VDAC1, UQCRC2, SLC25A5, PPARA, GAPDH, SLC25A6, CAMK2D, COX7A2, COX5B, COX5A, PRKCZ, RELA, CYC1, CAMK2G, NDUFV1, NDUFA9, NDUFA8, SMAD3, INSR, GFPT1, COX6C, MAPK13, COL1A1, COL3A1, COL1A2, SP1 | 1.26E-04 |
| KEGG_PATHWAY | hsa04148:Efferocytosis | 45 | CAMK2D, RAB5C, LRP1, ITGB5, PROS1, ALOX15, ATP2A3, SLC2A1, ATP2A2, LIPA, DUSP16, CASP7, RXRA, CASP3, P2RY2, BSG, CASP1, UQCRFS1, RAC1, HAVCR1, CAMK2G, MAPK3, DUSP4, CPT1A, MAP2K2, SLC16A1, ANO9, NR1H3, ANO6, ATP11A, DUSP8, MAPK13, GULP1, XKR9, RAB14, MAPKAPK2, TYRO3, PECAM1, ADAM9, RAB17, CALR, MFGE8, GAS6, RAB7A, PPARD | 1.65E-04 |
| KEGG_PATHWAY | hsa00010:Glycolysis / Gluconeogenesis | 25 | GPI, ACSS2, ENO1, ENO2, HK1, LDHA, ALDH2, ALDH3B1, PGK1, DLAT, PCK1, ACSS1, PCK2, TPI1, AKR1A1, ALDH3A2, PFKL, PKM, HKDC1, GALM, ALDOC, ALDOB, ALDOA, GAPDH, PFKP | 2.26E-04 |
| KEGG_PATHWAY | hsa05100:Bacterial invasion of epithelial cells | 27 | ARPC1B, PXN, CLTC, CLTB, ILK, CLTA, ACTB, ACTG1, CDH1, CTNNA1, RAC1, WASF2, ACTR3, ACTR2, RHOG, FN1, GAB1, ARPC4, ARPC5, RHOA, CD2AP, DNM2, CTTN, ARPC2, ARPC3, ELMO3, VCL | 3.25E-04 |
| KEGG_PATHWAY | hsa00020:Citrate cycle (TCA cycle) | 15 | MDH2, IDH1, SDHD, SDHA, SDHB, PC, OGDH, SUCLG2, SUCLG1, ACO1, DLAT, ACO2, PCK1, PCK2, IDH3A | 4.00E-04 |
| KEGG_PATHWAY | hsa05418:Fluid shear stress and atherosclerosis | 40 | GSTP1, MGST3, MGST1, MGST2, PDGFA, CALML4, PRKCZ, RELA, ACTB, ACTG1, HSP90B1, CTSL, SUMO3, RAC2, HMOX1, RAC1, MAP3K5, GSTM4, NQO1, JUN, MEF2C, EDN1, GSTO1, IL1R2, FOS, RHOA, ASS1, KLF2, TNFRSF1A, MAPK13, VEGFA, BMP4, GSTA2, GSTA1, PECAM1, SDC1, CALM3, CALM1, SQSTM1, NOX1 | 4.76E-04 |
| KEGG_PATHWAY | hsa05110:Vibrio cholerae infection | 20 | ERO1A, ATP6V0B, ARF1, ATP6V0E1, ATP6AP1, KDELR1, TCIRG1, ACTB, PDIA4, ACTG1, SEC61A1, KDELR2, KDELR3, ATP6V1E1, ATP6V0D1, ATP6V1C1, CFTR, TJP2, ATP6V1F, ATP6V0A1 | 5.48E-04 |
| KEGG_PATHWAY | hsa04210:Apoptosis | 39 | TRADD, CTSZ, ITPR3, CTSV, RELA, CTSS, ACTB, ACTG1, TUBA1C, CASP7, NRAS, CASP8, CTSL, CASP3, LMNA, CAPN2, CTSH, CAPN1, BID, CTSD, CTSC, MCL1, MAPK3, CTSB, MAP3K5, JUN, MAP2K2, PDPK1, TNFRSF10A, CFLAR, FOS, NGF, TNFRSF1A, GADD45G, NFKBIA, DDIT3, CYCS, ATF4, BCL2L1 | 6.03E-04 |
| KEGG_PATHWAY | hsa05010:Alzheimer disease | 86 | CHRM3, COX7B, IRS1, COX4I1, NDUFA10, ATP2A3, ATP2A2, FZD10, CALML4, COX6A1, PSMD8, PPP3CA, TUBA1C, TUBB6, CASP7, CASP8, TUBB3, CASP3, KIF5B, PSMD3, CAPN2, UQCRFS1, SLC39A5, CAPN1, SLC39A4, MAP3K5, COX8A, MAP2K2, WNT5B, MME, WNT5A, SLC39A11, WIPI1, SDHD, SDHA, SLC39A14, SDHB, COX6B1, TNFRSF1A, PLCB3, TUBB2A, NDUFS7, DDIT3, UQCRC1, PPIF, NDUFS2, VDAC2, VDAC1, UQCRC2, SLC25A5, GAPDH, SLC25A6, ATF4, LRP1, COX7A2, ITPR3, PSEN1, COX5B, COX5A, RELA, APH1A, WNT6, KLC4, NRAS, PPP3R1, APH1B, CYC1, APOE, BID, NDUFV1, MAPK3, NDUFA9, NDUFA8, XBP1, FZD7, INSR, TUBB4B, COX6C, PSMC4, GNAQ, ATG2A, CYCS, CALM3, CALM1, NOX1, MCU | 7.13E-04 |
| KEGG_PATHWAY | hsa04530:Tight junction | 45 | ARPC1B, NEDD4L, F11R, PRKCZ, CLDN1, MYL12A, ACTB, ACTG1, MYL12B, PPP2CB, TUBA1C, PARD6B, RAP1A, MARVELD3, CLDN23, MYH14, RAC1, SYNPO, RAB8B, MAP3K5, ACTR3, VASP, ACTR2, JUN, RDX, ARPC4, ACTN4, ARPC5, CGN, RHOA, RUNX1, CLDN4, MYL6, CTTN, ARPC2, ARPC3, CLDN7, PPP2R2D, CLDN18, EZR, CFTR, TJP3, TJP2, CRB3, LLGL2 | 0.00111361 |
| KEGG_PATHWAY | hsa05133:Pertussis | 25 | C1S, C1R, CALML4, RELA, CXCL5, C2, C3, PYCARD, CASP7, CASP3, CFL1, CASP1, MAPK3, JUN, FOS, IRAK4, TICAM1, RHOA, MAPK13, IRF1, IRF8, CALM3, CALM1, TLR4, MYD88 | 0.00161897 |
| KEGG_PATHWAY | hsa00051:Fructose and mannose metabolism | 15 | PFKFB2, PFKFB4, PFKFB3, TPI1, MPI, HK1, PFKL, AKR1B10, GMDS, ENOSF1, HKDC1, ALDOC, ALDOB, ALDOA, PFKP | 0.00161897 |
| KEGG_PATHWAY | hsa05205:Proteoglycans in cancer | 51 | CDKN1A, ITGB5, IHH, FZD10, ACTB, ACTG1, SHH, CTSL, CASP3, TIMP3, PLCE1, RAC1, VAV3, MAP2K2, WNT5B, PDPK1, ITGA2, WNT5A, PLAUR, GAB1, RRAS2, RHOA, CTTN, EZR, TLR4, CD44, CD63, CAMK2D, PXN, ITPR3, EGFR, SLC9A1, VTN, WNT6, NRAS, PAK1, RRAS, GPC3, FLNB, CAMK2G, MAPK3, LUM, FZD7, RDX, STAT3, FN1, MAPK13, VEGFA, COL1A1, COL1A2, SDC1 | 0.0017653 |
| KEGG_PATHWAY | hsa04141:Protein processing in endoplasmic reticulum | 44 | PPP1R15A, ERO1A, TRAM1, SEC23A, SAR1B, RPN1, RRBP1, UBE2J1, HSP90B1, HERPUD1, DNAJB1, SEC61A1, LMAN2, CAPN2, MAN1A1, FBXO6, CAPN1, UBXN6, SEC31A, MAP3K5, EDEM3, BCAP31, PDIA3, XBP1, SEC13, SEC24A, HSPA5, FBXO2, EDEM2, PDIA6, RAD23B, CKAP4, PDIA4, DNAJC3, DNAJC1, DDIT3, CANX, HYOU1, P4HB, CALR, SEC24D, SEC24C, ATF4, HSPA1A | 0.00200237 |

**B) INHIBITED**

| **Category** | **Term** | **Count** | **Genes** | **FDR** |
| --- | --- | --- | --- | --- |
| KEGG_PATHWAY | hsa03030:DNA replication | 18 | RNASEH2B, FEN1, RNASEH2A, PCNA, MCM7, RFC1, PRIM1, RPA2, POLD3, POLA1, RPA3, POLD1, MCM3, MCM4, MCM5, SSBP1, POLE, MCM2 | 2.62E-07 |
| KEGG_PATHWAY | hsa04110:Cell cycle | 38 | CDKN1C, YWHAE, PCNA, MCM7, PRKDC, CDCA5, PDS5B, SMC3, AURKB, CCNB1, ORC6, CDC23, CCND2, PTTG1, CCND1, CHEK1, E2F5, BUB1, CDT1, ANAPC7, ATRX, CDC6, SMC1A, CDC25A, CDC25B, CDK6, TFDP2, CDK4, MCM3, MCM4, MCM5, ATM, ANAPC5, TRIP13, TP53, MCM2, ATR, MAD2L1 | 8.18E-07 |
| KEGG_PATHWAY | hsa03440:Homologous recombination | 14 | XRCC2, RPA2, BRCA2, POLD3, BRIP1, RAD50, RAD51C, RPA3, POLD1, RBBP8, RAD54L, ATM, TOPBP1, SSBP1 | 0.00192506 |
| KEGG_PATHWAY | hsa03010:Ribosome | 31 | RPL4, RPL5, RPL3, RPL32, RPL31, RPL34, RPL11, RPS4Y1, RPL6, RPS4X, RPL7A, RPL36, RPL14, RPL35, RPL38, RPL37, RPL17, RPL21, RPS7, RPL23, RPL35A, RPL13A, MRPS21, RPS3A, RPS26, RPS25, RPS20, RPL22L1, RPL26, RPS24, RPS23 | 0.00573428 |
| KEGG_PATHWAY | hsa03082:ATP-dependent chromatin remodeling | 24 | SMARCE1, SS18, SMARCC1, PBRM1, MEAF6, PHF10, SMARCA5, CHD4, RSF1, BAZ1A, BAZ2B, ARID1A, SMARCA1, BAZ1B, SMARCA2, ARID1B, SMARCA4, MTA1, RBBP4, RUVBL2, DPF1, BRD8, ARID2, BPTF | 0.00590687 |
| KEGG_PATHWAY | hsa05171:Coronavirus disease - COVID-19 | 38 | RPL4, RPL5, NRP1, RPL3, RPL32, RPL31, RPL34, RPL11, PIK3R3, RPS4Y1, RPL6, RPS4X, RPL7A, C5, C7, RPL36, RPL14, RPL35, RPL38, PLCG1, RPL37, RPL17, RPL21, RPS7, STAT1, RPL23, RPL35A, RPL13A, RPS3A, MAPK12, RPS26, RPS25, AGTR1, RPS20, RPL22L1, RPL26, RPS24, RPS23 | 0.00598991 |
| KEGG_PATHWAY | hsa03013:Nucleocytoplasmic transport | 22 | IPO11, NUP107, NUP133, NUP210, CSE1L, THOC1, UPF3B, XPO7, AAAS, IPO4, IPO5, PNN, DDX19A, IPO9, XPO1, SUMO1, XPOT, TPR, KPNA6, KPNA5, TNPO2, KPNB1 | 0.00986792 |
| KEGG_PATHWAY | hsa03430:Mismatch repair | 9 | POLD3, MSH6, PCNA, MSH2, RFC1, POLD1, RPA3, RPA2, SSBP1 | 0.01448018 |
| KEGG_PATHWAY | hsa03460:Fanconi anemia pathway | 14 | FANCI, RMI2, FANCL, RPA2, BRCA2, FANCG, BRIP1, RAD51C, UBE2T, RPA3, USP1, REV3L, POLI, ATR | 0.01448018 |
| KEGG_PATHWAY | hsa04310:Wnt signaling pathway | 29 | CTBP2, CHD8, CTNND2, PRICKLE1, NLK, CXXC4, NKD1, LRP6, CCND2, CCND1, FRZB, BTRC, PRKACB, TCF7L1, TLE2, FZD2, SERPINF1, FZD6, FZD9, DKK1, TLE6, PLCB4, SFRP5, CTNNB1, ROR1, ROR2, PLCB1, LGR5, TP53 | 0.01915337 |
| KEGG_PATHWAY | hsa03410:Base excision repair | 12 | POLD3, FEN1, PCNA, NEIL2, RFC1, PARP1, PARP2, POLD1, TDP1, XRCC1, POLE, UNG | 0.02318925 |
| KEGG_PATHWAY | hsa04218:Cellular senescence | 26 | PIK3R3, FOXM1, ZFP36L1, CCNB1, CCND2, CCND1, RBBP4, CHEK1, EIF4EBP1, E2F5, BTRC, MAP2K6, IGFBP3, TGFBR1, MAPK12, CDC25A, HIPK2, TGFBR2, RAD50, MRAS, CDK6, CDK4, TRPV4, ATM, TP53, ATR | 0.03260548 |
| KEGG_PATHWAY | hsa05202:Transcriptional misregulation in cancer | 30 | DDX5, KMT2A, JMJD1C, BMI1, HHEX, CCND2, PROM1, SS18, ARNT2, TAF15, FUS, IGFBP3, PBX3, HMGA2, ETV1, MITF, ETV4, MLF1, ETV5, PBX1, DUSP6, ETV6, TGFBR2, MEIS1, ZEB1, FEV, ATM, TCF3, CDK14, TP53 | 0.03628742 |
| KEGG_PATHWAY | hsa05225:Hepatocellular carcinoma | 27 | PIK3R3, TGFA, LRP6, CCND1, DPF1, PLCG1, ARID2, SMARCE1, GSTM3, SMARCC1, PBRM1, TCF7L1, FZD2, PHF10, FZD6, FZD9, IGF2, ARID1A, SMARCA2, ARID1B, TGFBR1, SMARCA4, TGFBR2, CDK6, CDK4, CTNNB1, TP53 | 0.03784859 |
| KEGG_PATHWAY | hsa04512:ECM-receptor interaction | 17 | LAMA1, LAMB1, HMMR, HSPG2, RELN, FRAS1, SV2B, SV2A, COL4A6, SPP1, COL9A1, COL4A5, COL9A3, COL9A2, ITGA5, FREM1, FREM2 | 0.05882686 |
| KEGG_PATHWAY | hsa04950:Maturity onset diabetes of the young | 8 | NEUROD1, HHEX, ONECUT1, PDX1, PAX4, NKX6-1, MNX1, FOXA2 | 0.0911656 |
| KEGG_PATHWAY | hsa04974:Protein digestion and absorption | 18 | COL18A1, CPA2, CELA3A, COL26A1, CPA1, CPB2, SLC6A19, COL27A1, COL11A2, COL19A1, SLC8A3, FXYD2, COL4A6, COL9A1, COL4A5, COL9A3, COL9A2, PRSS2 | 0.09732535 |

**Table S4.** *Lists of* *induced transcripts shown in the Heatmaps reported in Figure 3G belonging to antigen processing and presentation process (A), Pathogenic Escherichia coli infection process (B), PPAR signaling pathway (C), Salmonella infection process (D), and Protein processing in endoplasmic reticulum process (E).*

**A) 33 induced transcripts shown in the Heatmap, belonging to the antigen processing and presentation process.**

| **Gene_name** | **PiMM1** | **PiMM2** | **PiMM3** | **PiMM4** | **PiZZ1** | **PiZZ2** | **PiZZ3** | **PiZZ4** | **PiZZ5** | **log2FoldChange** | **padj** |
| --- | --- | --- | --- | --- | --- | --- | --- | --- | --- | --- | --- |
| CD68 | 1.6325 | 6.5584 | 4.9531 | 4.0268 | 53.0451 | 84.3284 | 34.9681 | 46.4249 | 44.8892 | 3.536499 | 2.90E-15 |
| CTSS | 13.8763 | 10.931 | 10.8968 | 15.101 | 49.4489 | 56.5747 | 51.3594 | 67.5271 | 53.3059 | 2.024959 | 4.66E-13 |
| PSMB8 | 31.0176 | 28.42 | 45.5686 | 23.154 | 125.87 | 78.9911 | 113.646 | 117.117 | 86.9728 | 1.631581 | 2.19E-13 |
| CTSH | 501.179 | 464.55 | 501.254 | 421.81 | 1470.88 | 1263.86 | 1268.69 | 1719.83 | 1499.11 | 1.598847 | 1.60E-64 |
| RAB27A | 29.3851 | 41.537 | 28.728 | 26.174 | 99.7968 | 66.1818 | 76.4927 | 81.2436 | 85.1024 | 1.313163 | 8.22E-10 |
| TAP2 | 53.8727 | 71.05 | 73.3059 | 61.409 | 139.356 | 136.633 | 169.377 | 165.653 | 182.362 | 1.254228 | 1.90E-15 |
| HLA-C | 110.194 | 119.14 | 97.0808 | 104.7 | 258.033 | 306.357 | 226.2 | 278.549 | 240.344 | 1.253372 | 5.19E-23 |
| CTSD | 1288.05 | 1213.3 | 1270.97 | 1224.2 | 2909.39 | 2970.71 | 2610.59 | 3033.45 | 2702.7 | 1.180958 | 1.10E-91 |
| CTSE | 216.307 | 260.15 | 235.768 | 228.52 | 623.955 | 539.061 | 458.956 | 543.383 | 442.346 | 1.130581 | 1.92E-24 |
| HLA-E | 99.5828 | 107.12 | 105.996 | 83.557 | 193.3 | 246.58 | 225.107 | 190.975 | 189.844 | 1.042368 | 1.70E-14 |
| LGMN | 225.286 | 220.8 | 207.04 | 236.58 | 490.892 | 476.082 | 380.278 | 488.517 | 464.79 | 1.031648 | 1.38E-25 |
| TAPBPL | 0.81625 | 1.0931 | 1.98124 | 5.0335 | 11.6879 | 3.20234 | 8.74202 | 11.6062 | 9.35192 | 1.003534 | 0.01882 |
| PYCARD | 49.7914 | 57.933 | 59.4373 | 48.322 | 103.393 | 112.082 | 85.2347 | 113.952 | 94.4543 | 0.8533707 | 6.66E-07 |
| HLA-DRB1 | 0.81625 | 0 | 0 | 0 | 2.69721 | 5.33724 | 1.09275 | 1.05511 | 5.61115 | 0.8285452 | 0.04706 |
| UNC93B1 | 40.8126 | 36.071 | 33.6811 | 46.309 | 70.1275 | 64.0469 | 86.3274 | 67.5271 | 85.1024 | 0.825699 | 2.77E-05 |
| RFTN1 | 13.8763 | 8.7446 | 16.8406 | 8.0537 | 30.5684 | 24.5513 | 13.113 | 27.4329 | 28.0557 | 0.6768312 | 0.01744 |
| DNM2 | 311.808 | 347.6 | 335.82 | 293.96 | 548.433 | 531.589 | 450.214 | 475.855 | 498.457 | 0.6118889 | 5.20E-12 |
| SAR1B | 93.869 | 74.329 | 78.259 | 91.611 | 125.87 | 139.836 | 125.667 | 140.33 | 142.149 | 0.5968047 | 1.85E-05 |
| RAB6A | 142.844 | 182.54 | 143.64 | 180.2 | 248.143 | 264.727 | 269.91 | 286.99 | 201.066 | 0.5902594 | 3.60E-06 |
| TAP1 | 17.1413 | 17.489 | 13.8687 | 21.141 | 37.7609 | 45.9002 | 31.6898 | 28.488 | 16.8334 | 0.5587112 | 0.02672 |
| ARL8B | 171.413 | 191.29 | 199.115 | 160.07 | 262.528 | 247.648 | 251.333 | 288.045 | 276.817 | 0.5130299 | 1.27E-06 |
| ERAP1 | 84.074 | 63.398 | 59.4373 | 74.496 | 107.888 | 128.094 | 104.904 | 106.566 | 89.7784 | 0.4953942 | 0.00174 |
| RAB32 | 79.9927 | 100.56 | 84.2028 | 70.47 | 107.888 | 150.51 | 123.481 | 139.275 | 110.353 | 0.494163 | 0.00118 |
| CTSV | 36.7314 | 44.816 | 81.2309 | 52.349 | 85.4117 | 76.8562 | 83.0492 | 85.464 | 85.1024 | 0.4777279 | 0.01033 |
| EXT1 | 218.756 | 175.98 | 191.19 | 216.44 | 255.336 | 307.425 | 292.858 | 279.605 | 312.354 | 0.4773995 | 6.61E-06 |
| B2M | 1612.91 | 1703 | 1718.73 | 1596.6 | 2322.3 | 2790.31 | 1915.6 | 2763.34 | 2065.84 | 0.4762309 | 1.15E-06 |
| RAB8B | 53.0564 | 27.327 | 43.5873 | 36.242 | 72.8247 | 54.4398 | 60.1014 | 59.0863 | 61.7226 | 0.4370685 | 0.0213 |
| PSME1 | 503.628 | 478.76 | 522.057 | 454.03 | 659.018 | 699.178 | 568.231 | 658.39 | 589.171 | 0.3459884 | 3.18E-05 |
| PDIA3 | 1171.32 | 1227.5 | 1122.37 | 1169.8 | 1503.25 | 1389.82 | 1379.05 | 1624.87 | 1645.94 | 0.3448267 | 6.22E-07 |
| CTSL | 108.562 | 100.56 | 123.828 | 107.72 | 123.173 | 135.566 | 159.542 | 182.534 | 129.056 | 0.3179455 | 0.02601 |
| CALR | 2654.45 | 2840.9 | 2616.23 | 2702 | 3465.02 | 3337.91 | 3226.9 | 3481.87 | 3281.59 | 0.3049401 | 1.64E-10 |
| HLA-A | 519.136 | 491.88 | 484.414 | 401.68 | 551.13 | 618.052 | 603.199 | 538.107 | 523.707 | 0.2282272 | 0.01641 |
| RAB10 | 302.83 | 267.8 | 320.961 | 317.11 | 352.435 | 358.662 | 317.991 | 392.502 | 366.595 | 0.2137352 | 0.03349 |

**B) 66 induced transcripts shown in the Heatmap, belonging to the Pathogenic Escherichia coli infection process.**

| **Gene_name** | **PiMM1** | **PiMM2** | **PiMM3** | **PiMM4** | **PiZZ1** | **PiZZ2** | **PiZZ3** | **PiZZ4** | **PiZZ5** | **log2FoldChange** | **padj** |
| --- | --- | --- | --- | --- | --- | --- | --- | --- | --- | --- | --- |
| MYO1A | 1.6325 | 2.1861 | 6.9343 | 10.0671 | 85.4117 | 78.9911 | 75.3999 | 126.6134 | 85.1024 | 4.057408 | 2.41E-26 |
| CASP1 | 0.81625 | 0 | 0.9906 | 0 | 2.69721 | 10.6745 | 5.46376 | 20.04712 | 5.61115 | 2.52941 | 0.00237 |
| IL18 | 12.2438 | 7.6515 | 10.897 | 4.02684 | 35.0637 | 35.2258 | 50.2666 | 68.58226 | 54.2411 | 2.323104 | 6.05E-11 |
| TLR4 | 2.44876 | 3.2792 | 3.9625 | 2.01342 | 24.2749 | 16.0117 | 10.9275 | 10.55112 | 14.9631 | 1.968487 | 0.00019 |
| F2 | 146.925 | 172.71 | 156.52 | 150 | 520.562 | 503.835 | 484.089 | 820.8769 | 477.883 | 1.813707 | 5.91E-34 |
| MAPK3 | 165.699 | 160.68 | 181.28 | 154.026 | 512.47 | 477.149 | 429.452 | 503.2883 | 494.716 | 1.533339 | 1.32E-57 |
| TUBB2A | 100.399 | 89.632 | 109.96 | 93.6239 | 260.73 | 288.211 | 213.087 | 345.0215 | 254.372 | 1.432276 | 3.48E-23 |
| TUBB6 | 18.7738 | 25.141 | 23.775 | 24.161 | 50.3479 | 85.3958 | 56.8231 | 55.92092 | 55.1763 | 1.301204 | 1.59E-07 |
| CLDN7 | 380.374 | 373.83 | 425.97 | 388.59 | 852.318 | 898.791 | 870.924 | 1105.757 | 881.886 | 1.218827 | 3.24E-43 |
| MAPK13 | 216.307 | 237.2 | 220.91 | 213.422 | 395.591 | 461.137 | 448.029 | 501.1781 | 454.503 | 1.009956 | 9.08E-27 |
| FOS | 44.8939 | 44.816 | 37.644 | 34.2281 | 120.475 | 136.633 | 51.3594 | 70.69248 | 70.1394 | 1.001241 | 0.0001 |
| TUBA1C | 1439.05 | 1533.6 | 1564.2 | 1446.64 | 2683.72 | 2486.09 | 2802.91 | 3433.333 | 2799.03 | 0.9127744 | 1.25E-29 |
| MYH14 | 262.017 | 182.54 | 240.72 | 199.328 | 402.783 | 468.61 | 422.895 | 420.9896 | 402.132 | 0.9060616 | 6.86E-17 |
| CLDN4 | 620.352 | 529.05 | 575.55 | 470.133 | 908.96 | 1019.41 | 1055.6 | 1080.434 | 1031.52 | 0.8754154 | 2.26E-25 |
| WIPF1 | 22.8551 | 18.582 | 18.822 | 23.1543 | 35.0637 | 46.9677 | 43.7101 | 41.14936 | 46.7596 | 0.8630457 | 0.00041 |
| CLDN23 | 31.0176 | 39.351 | 27.737 | 33.2214 | 58.4396 | 84.3284 | 57.9159 | 51.70047 | 70.1394 | 0.8565006 | 8.68E-05 |
| PYCARD | 49.7914 | 57.933 | 59.437 | 48.322 | 103.393 | 112.082 | 85.2347 | 113.9521 | 94.4543 | 0.8533707 | 6.66E-07 |
| TRADD | 32.6501 | 20.768 | 15.85 | 24.161 | 44.0544 | 52.3049 | 37.1536 | 48.53514 | 52.3707 | 0.8113942 | 0.00088 |
| TNFRSF1A | 136.314 | 139.91 | 177.32 | 190.268 | 259.831 | 369.337 | 267.724 | 327.0846 | 237.539 | 0.8086085 | 3.61E-08 |
| IRAK4 | 29.3851 | 25.141 | 13.869 | 31.208 | 37.7609 | 53.3724 | 56.8231 | 40.09424 | 57.0467 | 0.7752413 | 0.00158 |
| TNFRSF10A | 24.4876 | 37.164 | 25.756 | 19.1275 | 53.9442 | 54.4398 | 49.1739 | 58.03114 | 38.3429 | 0.7675448 | 0.00099 |
| MYO1E | 70.1977 | 68.863 | 56.465 | 59.3958 | 91.7051 | 114.217 | 118.017 | 129.7787 | 115.964 | 0.7608018 | 2.95E-06 |
| CTTN | 571.377 | 524.67 | 484.41 | 505.368 | 895.474 | 861.43 | 847.976 | 925.333 | 925.84 | 0.7594554 | 2.04E-27 |
| CASP3 | 126.519 | 150.84 | 141.66 | 151.006 | 230.162 | 233.771 | 219.643 | 258.5024 | 262.789 | 0.727938 | 1.94E-10 |
| EZR | 903.591 | 831.83 | 881.65 | 872.817 | 1475.37 | 1495.49 | 1423.86 | 1473.991 | 1229.78 | 0.6873601 | 1.16E-23 |
| CLDN1 | 207.328 | 222.99 | 187.23 | 216.442 | 328.161 | 353.325 | 325.64 | 329.1949 | 330.123 | 0.6497274 | 4.54E-12 |
| JUN | 215.491 | 240.48 | 248.65 | 206.375 | 282.308 | 404.563 | 450.214 | 307.0375 | 376.882 | 0.6176125 | 2.21E-06 |
| CLDN18 | 1024.4 | 1107.3 | 1110.5 | 990.602 | 2841.96 | 1731.4 | 1473.03 | 1688.179 | 1280.28 | 0.5870104 | 0.00524 |
| SEC24A | 66.9327 | 62.305 | 83.212 | 78.5233 | 100.696 | 121.689 | 122.388 | 120.2827 | 113.158 | 0.5816553 | 0.00011 |
| BAIAP2L1 | 230.183 | 208.78 | 218.93 | 169.127 | 362.325 | 294.616 | 276.466 | 359.7931 | 304.872 | 0.5759418 | 9.68E-07 |
| ARPC1B | 277.526 | 286.38 | 286.29 | 277.852 | 390.196 | 426.979 | 407.597 | 439.9816 | 438.605 | 0.550132 | 3.99E-11 |
| ACTR3 | 262.017 | 309.34 | 284.31 | 253.691 | 393.793 | 385.349 | 402.133 | 438.9265 | 448.892 | 0.5475322 | 2.84E-09 |
| CASP4 | 24.4876 | 25.141 | 27.737 | 21.1409 | 56.6414 | 35.2258 | 40.4318 | 32.70846 | 43.0188 | 0.5383102 | 0.01478 |
| TUBB3 | 44.8939 | 45.909 | 49.531 | 44.2952 | 52.1461 | 66.1818 | 76.4927 | 86.51916 | 86.9728 | 0.5256391 | 0.0046 |
| CASP8 | 77.544 | 74.329 | 62.409 | 59.3958 | 105.191 | 106.745 | 110.368 | 101.2907 | 99.1303 | 0.5140509 | 0.00063 |
| RAB1A | 469.345 | 443.79 | 477.48 | 439.932 | 673.403 | 616.985 | 627.24 | 685.8226 | 705.134 | 0.5099416 | 3.10E-12 |
| CASP7 | 62.8514 | 38.257 | 53.494 | 69.4629 | 80.9163 | 88.5982 | 88.5129 | 84.40894 | 75.7505 | 0.4373516 | 0.00996 |
| MYD88 | 63.6677 | 44.816 | 51.512 | 56.3757 | 73.7237 | 75.7888 | 95.0695 | 74.91293 | 79.4913 | 0.4242586 | 0.01081 |
| MYO1C | 230.999 | 239.38 | 183.26 | 174.161 | 318.271 | 299.953 | 281.93 | 270.1086 | 260.918 | 0.4110234 | 0.00037 |
| CYTH1 | 84.8902 | 92.911 | 93.118 | 83.5569 | 151.943 | 122.756 | 115.832 | 115.0072 | 115.964 | 0.4065683 | 0.00409 |
| SEC24D | 76.7277 | 60.119 | 66.372 | 77.5166 | 109.687 | 113.149 | 85.2347 | 87.57427 | 105.677 | 0.4058586 | 0.00876 |
| ARPC2 | 980.319 | 1020.9 | 1001.5 | 996.642 | 1345.01 | 1407.96 | 1224.98 | 1387.472 | 1303.66 | 0.4013661 | 8.99E-12 |
| ARPC3 | 414.656 | 433.95 | 455.69 | 393.623 | 617.661 | 595.636 | 490.646 | 610.9097 | 534.93 | 0.3936158 | 7.95E-06 |
| WASF2 | 518.32 | 529.05 | 488.38 | 485.234 | 662.615 | 695.976 | 638.167 | 709.0351 | 639.671 | 0.3832421 | 6.86E-08 |
| PAK1 | 398.331 | 389.13 | 356.62 | 385.57 | 473.81 | 494.228 | 512.501 | 551.8234 | 509.679 | 0.3824778 | 1.49E-06 |
| F2R | 39.9964 | 47.002 | 52.503 | 50.3355 | 56.6414 | 59.7771 | 54.6376 | 86.51916 | 86.9728 | 0.379625 | 0.03922 |
| TAB3 | 65.3002 | 53.56 | 47.55 | 86.577 | 90.8061 | 92.8679 | 81.9564 | 84.40894 | 99.1303 | 0.3763942 | 0.02484 |
| MYO1D | 129.784 | 123.52 | 119.87 | 108.725 | 121.374 | 163.319 | 192.324 | 160.377 | 195.455 | 0.3757857 | 0.00846 |
| TUBB4B | 1252.13 | 1325.9 | 1191.7 | 1221.14 | 1667.77 | 1438.92 | 1668.63 | 1743.045 | 1640.33 | 0.3697761 | 1.45E-08 |
| CYCS | 415.472 | 383.67 | 456.68 | 391.61 | 549.332 | 532.656 | 467.698 | 637.2875 | 530.254 | 0.3625825 | 0.00013 |
| ACTG1 | 11700.2 | 10714 | 11849 | 11390.9 | 14645.9 | 14456.4 | 15214.4 | 15857.27 | 13739.8 | 0.3614942 | 3.80E-12 |
| NCK2 | 206.512 | 203.31 | 180.29 | 200.335 | 231.061 | 257.255 | 246.962 | 332.3602 | 249.696 | 0.3553528 | 0.00265 |
| SEC24C | 144.477 | 160.68 | 156.52 | 150 | 195.098 | 182.534 | 209.808 | 190.9752 | 220.705 | 0.3381415 | 0.00293 |
| NFKBIA | 302.83 | 312.62 | 287.28 | 294.966 | 363.224 | 352.258 | 395.576 | 403.0527 | 367.53 | 0.3007382 | 0.00065 |
| MYO6 | 704.426 | 658.03 | 693.43 | 689.596 | 878.391 | 920.14 | 842.512 | 826.1525 | 808.941 | 0.2996449 | 6.59E-06 |
| RELA | 150.19 | 173.8 | 159.49 | 166.107 | 191.502 | 180.399 | 255.704 | 195.1957 | 213.224 | 0.2950202 | 0.01785 |
| ARF1 | 1212.95 | 1313.9 | 1271 | 1371.14 | 1492.46 | 1609.71 | 1547.34 | 1714.557 | 1569.25 | 0.2828982 | 5.18E-06 |
| TMBIM6 | 2234.08 | 2297.6 | 2273.5 | 2196.64 | 2716.09 | 2657.94 | 2578.9 | 2899.447 | 2770.97 | 0.2674479 | 9.95E-08 |
| ARPC4 | 808.09 | 854.78 | 835.09 | 836.575 | 1048.32 | 894.521 | 1046.86 | 1022.403 | 989.433 | 0.2495442 | 0.00025 |
| RHOA | 1371.3 | 1288.7 | 1303.7 | 1335.9 | 1598.55 | 1539.26 | 1466.47 | 1642.809 | 1676.8 | 0.2471612 | 2.97E-05 |
| ARPC5 | 331.398 | 313.71 | 327.9 | 279.865 | 369.518 | 377.876 | 377 | 404.1078 | 359.114 | 0.2407132 | 0.0092 |
| RAC1 | 1360.69 | 1309.5 | 1414.6 | 1319.8 | 1560.79 | 1658.81 | 1553.89 | 1625.927 | 1569.25 | 0.22865 | 2.51E-05 |
| GAPDH | 6890.8 | 7902.9 | 7474.2 | 6966.43 | 9056.33 | 9339.1 | 7664.57 | 9541.375 | 7681.66 | 0.2259188 | 0.00499 |
| ACTB | 2179.39 | 2409.1 | 2452.8 | 2516.77 | 2841.96 | 2924.81 | 2963.54 | 2709.527 | 2608.25 | 0.2227876 | 0.00041 |
| ARF6 | 243.243 | 254.69 | 252.61 | 261.744 | 321.867 | 318.099 | 290.672 | 291.2108 | 268.4 | 0.2082851 | 0.04226 |
| ACTR2 | 710.956 | 687.54 | 718.2 | 720.804 | 854.117 | 764.292 | 720.124 | 834.5934 | 915.552 | 0.1889529 | 0.02444 |

**C) 34 induced transcripts shown in the Heatmap, belonging to the PPAR signaling pathway.**

| **Gene_name** | **PiMM1** | **PiMM2** | **PiMM3** | **PiMM4** | **PiZZ1** | **PiZZ2** | **PiZZ3** | **PiZZ4** | **PiZZ5** | **log2FoldChange** | **padj** |
| --- | --- | --- | --- | --- | --- | --- | --- | --- | --- | --- | --- |
| FABP2 | 0 | 0 | 3.96248 | 1.00671 | 106.09 | 150.51 | 136.594 | 202.581 | 146.825 | 6.553693 | 2.56E-23 |
| APOC3 | 14.6925 | 7.6515 | 17.8312 | 10.0671 | 673.403 | 896.656 | 643.631 | 1125.8 | 783.69 | 5.951796 | 2.27E-88 |
| FABP6 | 0 | 2.1861 | 0 | 0 | 24.2749 | 37.3607 | 40.4318 | 66.472 | 20.5742 | 5.448375 | 4.92E-08 |
| ACSL5 | 11.4275 | 9.8376 | 14.8593 | 12.0805 | 237.354 | 380.011 | 240.406 | 433.651 | 360.984 | 4.729455 | 2.61E-70 |
| APOA1 | 441.593 | 679.89 | 640.932 | 578.858 | 5807.09 | 5244.37 | 5864.8 | 8929.41 | 5790.71 | 3.407093 | 1.07E-60 |
| FABP1 | 382.006 | 358.53 | 404.173 | 379.529 | 3174.62 | 4131.02 | 3128.55 | 4649.88 | 3232.02 | 3.256158 | 4.72E-194 |
| MMP1 | 62.0352 | 38.257 | 59.4373 | 32.2147 | 375.811 | 554.005 | 392.298 | 481.131 | 348.826 | 3.126549 | 5.19E-67 |
| PCK1 | 2.44876 | 4.3723 | 3.96248 | 3.02013 | 27.8712 | 36.2932 | 18.5768 | 32.7085 | 19.639 | 2.773719 | 1.98E-08 |
| HMGCS2 | 208.961 | 169.43 | 186.237 | 183.221 | 715.66 | 655.413 | 780.225 | 1122.64 | 814.552 | 2.104119 | 5.96E-55 |
| GK | 29.3851 | 12.024 | 22.7843 | 12.0805 | 53.0451 | 91.8005 | 86.3274 | 107.621 | 80.4265 | 2.007893 | 8.79E-13 |
| PCK2 | 32.6501 | 37.164 | 70.3341 | 46.3086 | 169.924 | 140.903 | 198.881 | 227.904 | 172.075 | 1.91649 | 2.69E-21 |
| FABP3 | 28.5688 | 31.699 | 30.7092 | 28.1879 | 95.3014 | 148.375 | 68.8434 | 69.6374 | 61.7226 | 1.463175 | 2.88E-08 |
| PPARA | 88.1552 | 87.446 | 83.2122 | 91.6105 | 169.924 | 219.894 | 126.759 | 223.684 | 173.946 | 1.007927 | 1.20E-09 |
| NR1H3 | 36.7314 | 49.188 | 29.7186 | 44.2952 | 80.0172 | 104.61 | 71.0289 | 101.291 | 60.7874 | 0.9612815 | 1.07E-05 |
| ME3 | 12.2438 | 4.3723 | 9.90621 | 5.03355 | 23.3758 | 18.1466 | 21.855 | 17.9369 | 17.7686 | 0.8924862 | 0.00692689 |
| SLC27A2 | 47.3426 | 38.257 | 26.7468 | 38.2549 | 55.7423 | 81.126 | 67.7507 | 80.1885 | 88.8432 | 0.8646645 | 5.19E-05 |
| PLIN2 | 715.853 | 629.61 | 725.134 | 669.462 | 1284.77 | 1335.38 | 1059.97 | 1486.65 | 1029.65 | 0.8316466 | 2.43E-16 |
| ME1 | 26.9363 | 16.396 | 26.7468 | 30.2013 | 61.1368 | 40.563 | 29.5043 | 49.5903 | 44.8892 | 0.6146976 | 0.00872908 |
| SCP2 | 229.367 | 206.59 | 200.105 | 194.295 | 326.362 | 311.695 | 295.043 | 386.171 | 307.678 | 0.6056952 | 1.22E-08 |
| CYP27A1 | 52.2401 | 59.026 | 40.6155 | 42.2818 | 65.6321 | 64.0469 | 84.1419 | 103.401 | 87.908 | 0.5984219 | 0.00166346 |
| PPARD | 21.2226 | 24.048 | 27.7374 | 31.208 | 49.4489 | 35.2258 | 31.6898 | 51.7005 | 47.6948 | 0.5159704 | 0.0174518 |
| HMGCS1 | 804.009 | 761.87 | 795.469 | 848.656 | 1095.97 | 1108.01 | 1118.98 | 1344.21 | 988.497 | 0.4654198 | 1.50E-08 |
| ACSL1 | 23.6713 | 22.954 | 36.653 | 16.1073 | 50.3479 | 40.563 | 32.7826 | 39.0391 | 39.278 | 0.458039 | 0.03603532 |
| SCD | 1250.5 | 1359.8 | 1335.36 | 1306.71 | 1816.12 | 1982.25 | 1526.58 | 1879.15 | 1593.57 | 0.3970424 | 3.15E-07 |
| PLTP | 73.4627 | 89.632 | 93.1184 | 72.4831 | 123.173 | 102.475 | 138.78 | 97.0703 | 108.482 | 0.3756064 | 0.01316926 |
| CPT1A | 191.003 | 191.29 | 198.124 | 192.281 | 202.291 | 283.941 | 263.353 | 279.605 | 259.048 | 0.3592122 | 0.00165814 |
| ACSL3 | 398.331 | 415.37 | 400.211 | 434.898 | 564.616 | 518.78 | 478.626 | 590.863 | 492.846 | 0.3328703 | 0.00010489 |
| DBI | 1693.72 | 1659.3 | 1679.1 | 1794.96 | 2203.62 | 2179.73 | 2051.1 | 2438.36 | 1983.54 | 0.3300388 | 4.65E-07 |
| RXRA | 217.123 | 197.85 | 225.862 | 173.154 | 243.648 | 247.648 | 260.075 | 262.723 | 303.002 | 0.321737 | 0.00429614 |
| ILK | 181.208 | 208.78 | 191.19 | 204.362 | 219.373 | 293.548 | 226.2 | 254.282 | 245.955 | 0.2894024 | 0.01169174 |
| PDPK1 | 192.636 | 162.87 | 175.34 | 186.241 | 187.906 | 211.355 | 250.24 | 250.061 | 231.927 | 0.279297 | 0.01971759 |
| UBC | 1348.45 | 1325.9 | 1254.13 | 1244.29 | 1630.91 | 1590.5 | 1424.95 | 1535.19 | 1622.56 | 0.2592358 | 2.38E-05 |
| ACOX1 | 293.035 | 276.55 | 265.486 | 268.791 | 327.262 | 305.29 | 332.197 | 320.754 | 352.567 | 0.2205183 | 0.02146482 |
| ACAA1 | 324.868 | 286.38 | 296.196 | 299.999 | 353.335 | 353.325 | 370.443 | 355.573 | 346.956 | 0.2110419 | 0.02125782 |

**D) 74 induced transcripts shown in the Heatmap, belonging to the Salmonella infection process.**

| **Gene_name** | **PiMM1** | **PiMM2** | **PiMM3** | **PiMM4** | **PiZZ1** | **PiZZ2** | **PiZZ3** | **PiZZ4** | **PiZZ5** | **log2FoldChange** | **padj** |
| --- | --- | --- | --- | --- | --- | --- | --- | --- | --- | --- | --- |
| CASP1 | 0.81625 | 0 | 0.99062 | 0 | 2.69721 | 10.6745 | 5.46376 | 20.0471 | 5.61115 | 2.52941 | 0.002367 |
| IL18 | 12.2438 | 7.65149 | 10.8968 | 4.0268 | 35.0637 | 35.2258 | 50.2666 | 68.5823 | 54.2411 | 2.323104 | 6.05E-11 |
| TLR4 | 2.44876 | 3.27921 | 3.96248 | 2.0134 | 24.2749 | 16.0117 | 10.9275 | 10.5511 | 14.9631 | 1.968487 | 0.000188 |
| S100A10 | 648.104 | 522.487 | 610.222 | 527.52 | 1919.51 | 2140.23 | 2242.33 | 2518.55 | 2114.47 | 1.909535 | 9.80E-110 |
| AHNAK | 97.9503 | 83.0733 | 101.043 | 78.523 | 331.757 | 387.483 | 322.362 | 309.148 | 286.169 | 1.832335 | 3.77E-46 |
| RILP | 17.1413 | 14.2099 | 12.8781 | 20.134 | 53.0451 | 68.3166 | 56.8231 | 70.6925 | 44.8892 | 1.757694 | 6.13E-11 |
| MLKL | 0 | 1.09307 | 0.99062 | 6.0403 | 16.1833 | 11.7419 | 13.113 | 6.33067 | 8.41672 | 1.657636 | 0.002967 |
| MAPK3 | 165.699 | 160.681 | 181.284 | 154.03 | 512.47 | 477.149 | 429.452 | 503.288 | 494.716 | 1.533339 | 1.32E-57 |
| ANXA2 | 759.931 | 685.355 | 762.778 | 690.6 | 1856.58 | 2094.33 | 1963.68 | 2083.85 | 1946.13 | 1.448132 | 1.62E-121 |
| TUBB2A | 100.399 | 89.6317 | 109.959 | 93.624 | 260.73 | 288.211 | 213.087 | 345.022 | 254.372 | 1.432276 | 3.48E-23 |
| PLEKHM1 | 32.6501 | 38.2574 | 37.6436 | 21.141 | 67.4303 | 80.0586 | 98.3477 | 94.9601 | 86.9728 | 1.32106 | 6.86E-10 |
| TUBB6 | 18.7738 | 25.1406 | 23.7749 | 24.161 | 50.3479 | 85.3958 | 56.8231 | 55.9209 | 55.1763 | 1.301204 | 1.59E-07 |
| FLNB | 223.653 | 238.289 | 214.965 | 201.34 | 488.195 | 520.914 | 463.327 | 511.729 | 415.225 | 1.110817 | 1.21E-30 |
| AHNAK2 | 7.34627 | 8.74456 | 8.91559 | 9.0604 | 29.6693 | 18.1466 | 31.6898 | 15.8267 | 16.8334 | 1.055343 | 0.002316 |
| MAPK13 | 216.307 | 237.196 | 220.908 | 213.42 | 395.591 | 461.137 | 448.029 | 501.178 | 454.503 | 1.009956 | 9.08E-27 |
| FOS | 44.8939 | 44.8159 | 37.6436 | 34.228 | 120.475 | 136.633 | 51.3594 | 70.6925 | 70.1394 | 1.001241 | 0.0001 |
| RRAS | 44.8939 | 52.4674 | 51.5123 | 55.369 | 98.8977 | 117.419 | 130.038 | 97.0703 | 72.0097 | 0.9318719 | 1.49E-06 |
| TUBA1C | 1439.05 | 1533.58 | 1564.19 | 1446.6 | 2683.72 | 2486.09 | 2802.91 | 3433.33 | 2799.03 | 0.9127744 | 1.25E-29 |
| KLC4 | 73.4627 | 66.6773 | 84.2028 | 60.403 | 100.696 | 164.387 | 135.501 | 154.046 | 141.214 | 0.8976098 | 1.52E-07 |
| PYCARD | 49.7914 | 57.9327 | 59.4373 | 48.322 | 103.393 | 112.082 | 85.2347 | 113.952 | 94.4543 | 0.8533707 | 6.66E-07 |
| TRADD | 32.6501 | 20.7683 | 15.8499 | 24.161 | 44.0544 | 52.3049 | 37.1536 | 48.5351 | 52.3707 | 0.8113942 | 0.000878 |
| TNFRSF1A | 136.314 | 139.913 | 177.321 | 190.27 | 259.831 | 369.337 | 267.724 | 327.085 | 237.539 | 0.8086085 | 3.61E-08 |
| IRAK4 | 29.3851 | 25.1406 | 13.8687 | 31.208 | 37.7609 | 53.3724 | 56.8231 | 40.0942 | 57.0467 | 0.7752413 | 0.001585 |
| TNFRSF10A | 24.4876 | 37.1644 | 25.7561 | 19.127 | 53.9442 | 54.4398 | 49.1739 | 58.0311 | 38.3429 | 0.7675448 | 0.000985 |
| MAP2K2 | 233.448 | 263.43 | 295.205 | 359.4 | 568.212 | 520.914 | 533.263 | 511.729 | 381.558 | 0.7637713 | 4.36E-09 |
| PODXL | 36.7314 | 20.7683 | 27.7374 | 35.235 | 58.4396 | 55.5073 | 59.0086 | 54.8658 | 56.1115 | 0.763576 | 0.000359 |
| CASP3 | 126.519 | 150.844 | 141.659 | 151.01 | 230.162 | 233.771 | 219.643 | 258.502 | 262.789 | 0.727938 | 1.94E-10 |
| SNX33 | 67.7489 | 60.1188 | 60.4279 | 71.476 | 113.283 | 96.0703 | 113.646 | 105.511 | 125.316 | 0.6952314 | 8.66E-06 |
| MYL12B | 969.708 | 912.713 | 960.902 | 859.73 | 1408.84 | 1643.87 | 1416.21 | 1604.82 | 1416.82 | 0.6786324 | 1.61E-22 |
| JUN | 215.491 | 240.475 | 248.646 | 206.38 | 282.308 | 404.563 | 450.214 | 307.038 | 376.882 | 0.6176125 | 2.21E-06 |
| DNM2 | 311.808 | 347.596 | 335.82 | 293.96 | 548.433 | 531.589 | 450.214 | 475.855 | 498.457 | 0.6118889 | 5.20E-12 |
| RHOG | 107.745 | 106.028 | 111.94 | 128.86 | 171.722 | 180.399 | 171.562 | 165.653 | 192.649 | 0.5803435 | 2.39E-06 |
| ARPC1B | 277.526 | 286.384 | 286.289 | 277.85 | 390.196 | 426.979 | 407.597 | 439.982 | 438.605 | 0.550132 | 3.99E-11 |
| ACTR3 | 262.017 | 309.339 | 284.308 | 253.69 | 393.793 | 385.349 | 402.133 | 438.926 | 448.892 | 0.5475322 | 2.84E-09 |
| CASP4 | 24.4876 | 25.1406 | 27.7374 | 21.141 | 56.6414 | 35.2258 | 40.4318 | 32.7085 | 43.0188 | 0.5383102 | 0.014781 |
| TUBB3 | 44.8939 | 45.9089 | 49.531 | 44.295 | 52.1461 | 66.1818 | 76.4927 | 86.5192 | 86.9728 | 0.5256391 | 0.0046 |
| CASP8 | 77.544 | 74.3287 | 62.4091 | 59.396 | 105.191 | 106.745 | 110.368 | 101.291 | 99.1303 | 0.5140509 | 0.000634 |
| ARL8B | 171.413 | 191.287 | 199.115 | 160.07 | 262.528 | 247.648 | 251.333 | 288.045 | 276.817 | 0.5130299 | 1.27E-06 |
| RIPK3 | 17.9576 | 6.55842 | 11.8875 | 15.101 | 26.9721 | 24.5513 | 19.6695 | 25.3227 | 22.4446 | 0.5071194 | 0.044583 |
| RAB5C | 578.723 | 614.305 | 620.129 | 541.61 | 783.09 | 876.374 | 781.318 | 859.916 | 789.302 | 0.4527105 | 2.06E-10 |
| PKN1 | 61.2189 | 99.4694 | 107.978 | 121.81 | 165.429 | 179.331 | 156.264 | 161.432 | 81.3617 | 0.4394913 | 0.020509 |
| CASP7 | 62.8514 | 38.2574 | 53.4935 | 69.463 | 80.9163 | 88.5982 | 88.5129 | 84.4089 | 75.7505 | 0.4373516 | 0.009965 |
| SNX9 | 126.519 | 151.937 | 157.509 | 123.83 | 199.594 | 241.243 | 179.211 | 195.196 | 174.881 | 0.4319535 | 0.000816 |
| MYD88 | 63.6677 | 44.8159 | 51.5123 | 56.376 | 73.7237 | 75.7888 | 95.0695 | 74.9129 | 79.4913 | 0.4242586 | 0.010812 |
| CYTH1 | 84.8902 | 92.9109 | 93.1184 | 83.557 | 151.943 | 122.756 | 115.832 | 115.007 | 115.964 | 0.4065683 | 0.004088 |
| ARPC2 | 980.319 | 1020.93 | 1001.52 | 996.64 | 1345.01 | 1407.96 | 1224.98 | 1387.47 | 1303.66 | 0.4013661 | 8.99E-12 |
| RALA | 257.936 | 298.408 | 272.421 | 331.21 | 414.471 | 430.181 | 338.753 | 398.832 | 378.753 | 0.3961679 | 8.77E-05 |
| ARPC3 | 414.656 | 433.949 | 455.686 | 393.62 | 617.661 | 595.636 | 490.646 | 610.91 | 534.93 | 0.3936158 | 7.95E-06 |
| PAK1 | 398.331 | 389.133 | 356.624 | 385.57 | 473.81 | 494.228 | 512.501 | 551.823 | 509.679 | 0.3824778 | 1.49E-06 |
| TAB3 | 65.3002 | 53.5604 | 47.5498 | 86.577 | 90.8061 | 92.8679 | 81.9564 | 84.4089 | 99.1303 | 0.3763942 | 0.024843 |
| TUBB4B | 1252.13 | 1325.89 | 1191.72 | 1221.1 | 1667.77 | 1438.92 | 1668.63 | 1743.04 | 1640.33 | 0.3697761 | 1.45E-08 |
| CYCS | 415.472 | 383.668 | 456.676 | 391.61 | 549.332 | 532.656 | 467.698 | 637.287 | 530.254 | 0.3625825 | 0.000133 |
| ACTG1 | 11700.2 | 10714.3 | 11848.8 | 11391 | 14645.9 | 14456.4 | 15214.4 | 15857.3 | 13739.8 | 0.3614942 | 3.80E-12 |
| GSDMD | 63.6677 | 79.7941 | 79.2497 | 59.396 | 87.2098 | 99.2726 | 99.4405 | 102.346 | 82.2969 | 0.3157131 | 0.042151 |
| NFKBIA | 302.83 | 312.618 | 287.28 | 294.97 | 363.224 | 352.258 | 395.576 | 403.053 | 367.53 | 0.3007382 | 0.000652 |
| MYO6 | 704.426 | 658.028 | 693.435 | 689.6 | 878.391 | 920.14 | 842.512 | 826.152 | 808.941 | 0.2996449 | 6.59E-06 |
| RELA | 150.19 | 173.798 | 159.49 | 166.11 | 191.502 | 180.399 | 255.704 | 195.196 | 213.224 | 0.2950202 | 0.017847 |
| DCTN2 | 322.42 | 342.131 | 424.976 | 335.23 | 458.526 | 452.598 | 467.698 | 454.753 | 401.197 | 0.2935305 | 0.002786 |
| ARF1 | 1212.95 | 1313.87 | 1270.97 | 1371.1 | 1492.46 | 1609.71 | 1547.34 | 1714.56 | 1569.25 | 0.2828982 | 5.18E-06 |
| MAP2K3 | 252.222 | 274.361 | 299.167 | 316.11 | 345.243 | 333.044 | 347.495 | 398.832 | 335.734 | 0.2696921 | 0.007824 |
| ARL8A | 357.519 | 300.594 | 323.933 | 324.16 | 313.775 | 391.753 | 401.04 | 449.478 | 456.373 | 0.2551847 | 0.025447 |
| ARPC4 | 808.09 | 854.781 | 835.093 | 836.58 | 1048.32 | 894.521 | 1046.86 | 1022.4 | 989.433 | 0.2495442 | 0.000247 |
| RHOA | 1371.3 | 1288.73 | 1303.66 | 1335.9 | 1598.55 | 1539.26 | 1466.47 | 1642.81 | 1676.8 | 0.2471612 | 2.97E-05 |
| MYL12A | 1149.28 | 1140.07 | 1127.33 | 1037.9 | 1311.74 | 1388.75 | 1176.89 | 1370.59 | 1414.01 | 0.2437257 | 0.000507 |
| ARPC5 | 331.398 | 313.711 | 327.895 | 279.87 | 369.518 | 377.876 | 377 | 404.108 | 359.114 | 0.2407132 | 0.009198 |
| KIF5B | 1017.05 | 975.018 | 967.837 | 1041.9 | 1203.85 | 1199.81 | 1103.68 | 1237.65 | 1200.79 | 0.2378255 | 9.56E-05 |
| RAC1 | 1360.69 | 1309.5 | 1414.61 | 1319.8 | 1560.79 | 1658.81 | 1553.89 | 1625.93 | 1569.25 | 0.22865 | 2.51E-05 |
| GAPDH | 6890.8 | 7902.89 | 7474.23 | 6966.4 | 9056.33 | 9339.1 | 7664.57 | 9541.38 | 7681.66 | 0.2259188 | 0.004993 |
| ACTB | 2179.39 | 2409.13 | 2452.78 | 2516.8 | 2841.96 | 2924.81 | 2963.54 | 2709.53 | 2608.25 | 0.2227876 | 0.000413 |
| RAB7A | 524.85 | 596.816 | 572.579 | 570.8 | 650.927 | 688.504 | 601.014 | 695.319 | 679.884 | 0.2134566 | 0.006764 |
| ARF6 | 243.243 | 254.685 | 252.608 | 261.74 | 321.867 | 318.099 | 290.672 | 291.211 | 268.4 | 0.2082851 | 0.04226 |
| HSP90B1 | 3169.51 | 3261.72 | 3241.31 | 3374.5 | 3949.61 | 3822.53 | 3539.43 | 3937.68 | 3626.67 | 0.2038975 | 0.000109 |
| ACTR2 | 710.956 | 687.541 | 718.2 | 720.8 | 854.117 | 764.292 | 720.124 | 834.593 | 915.552 | 0.1889529 | 0.024436 |
| DYNC1H1 | 797.479 | 804.499 | 826.178 | 730.87 | 901.767 | 841.149 | 937.582 | 934.829 | 852.895 | 0.1657181 | 0.024686 |

**E) 34 induced transcripts shown in the Heatmap, belonging to the Protein processing in endoplasmic reticulum process.**

| **Gene_name** | **PiMM1** | **PiMM2** | **PiMM3** | **PiMM4** | **PiZZ1** | **PiZZ2** | **PiZZ3** | **PiZZ4** | **PiZZ5** | **log2FoldChange** | **padj** |
| --- | --- | --- | --- | --- | --- | --- | --- | --- | --- | --- | --- |
| HSPA1A | 25.3038 | 24.0475 | 24.7655 | 15.1006 | 124.0717 | 96.0703 | 103.8115 | 110.7867 | 94.4543 | 2.189323 | 3.63E-24 |
| FBXO2 | 85.7065 | 69.9565 | 70.3341 | 61.4093 | 250.8405 | 276.469 | 243.6838 | 314.4233 | 249.696 | 1.858797 | 3.26E-41 |
| MAP3K5 | 4.08126 | 7.65149 | 2.97186 | 3.02013 | 17.9814 | 25.6187 | 8.74202 | 16.88179 | 14.0279 | 1.467375 | 0.001061 |
| HERPUD1 | 159.169 | 163.96 | 183.265 | 142.953 | 426.1592 | 388.551 | 356.2373 | 423.0998 | 334.799 | 1.227879 | 2.42E-28 |
| FBXO6 | 8.16252 | 7.65149 | 5.94373 | 8.05367 | 11.68791 | 29.8885 | 18.57679 | 17.9369 | 20.5742 | 0.9861847 | 0.00473 |
| CAPN2 | 91.4203 | 72.1426 | 105.996 | 80.5367 | 167.227 | 196.41 | 179.2114 | 155.1014 | 176.751 | 0.9536083 | 2.22E-11 |
| DDIT3 | 50.6076 | 31.699 | 49.531 | 45.3019 | 102.494 | 112.082 | 83.04919 | 90.73961 | 68.269 | 0.94508 | 2.99E-06 |
| ERO1A | 208.144 | 212.056 | 269.449 | 207.382 | 363.2243 | 528.387 | 275.3736 | 377.73 | 319.835 | 0.6571793 | 1.65E-05 |
| SEC23A | 182.024 | 169.426 | 175.34 | 190.268 | 248.1433 | 256.187 | 275.3736 | 378.7851 | 267.465 | 0.6097822 | 1.96E-06 |
| SAR1B | 93.869 | 74.3287 | 78.259 | 91.6105 | 125.8698 | 139.836 | 125.6665 | 140.3299 | 142.149 | 0.5968047 | 1.85E-05 |
| SEC24A | 66.9327 | 62.305 | 83.2122 | 78.5233 | 100.6958 | 121.689 | 122.3883 | 120.2827 | 113.158 | 0.5816553 | 0.00011 |
| P4HB | 1300.29 | 1229.7 | 1341.3 | 1274.49 | 1789.149 | 1941.69 | 1891.555 | 2124.995 | 1846.07 | 0.5613422 | 3.05E-20 |
| HSPA5 | 2333.67 | 2469.24 | 2480.51 | 2262.08 | 3344.541 | 3405.16 | 3457.469 | 3967.22 | 3392.87 | 0.5435429 | 2.44E-19 |
| PPP1R15A | 99.5828 | 106.028 | 105.996 | 85.5703 | 139.3559 | 163.319 | 140.9651 | 176.2037 | 135.603 | 0.5291165 | 0.000108 |
| DNAJC3 | 185.289 | 173.798 | 191.19 | 220.469 | 260.7303 | 304.223 | 222.9215 | 292.2659 | 324.511 | 0.4845223 | 5.70E-05 |
| CAPN1 | 240.794 | 286.384 | 300.158 | 273.825 | 383.9029 | 398.158 | 437.101 | 380.8953 | 368.465 | 0.4817369 | 2.73E-07 |
| MAN1A1 | 166.515 | 196.753 | 161.471 | 186.241 | 239.1526 | 229.501 | 244.7766 | 277.4944 | 291.78 | 0.4804584 | 1.99E-05 |
| TRAM1 | 266.098 | 277.64 | 254.59 | 296.979 | 382.1048 | 420.574 | 333.2895 | 424.1549 | 364.725 | 0.4522035 | 3.15E-06 |
| XBP1 | 413.024 | 416.46 | 366.53 | 434.898 | 605.0742 | 567.882 | 554.0255 | 594.0279 | 509.679 | 0.4447983 | 5.86E-08 |
| PDIA4 | 587.702 | 593.537 | 668.669 | 588.925 | 846.0249 | 813.395 | 885.1295 | 890.5143 | 712.616 | 0.4159254 | 2.27E-07 |
| SEC13 | 262.833 | 291.85 | 361.577 | 317.113 | 410.875 | 373.607 | 470.9763 | 452.6429 | 404.003 | 0.409227 | 9.16E-05 |
| SEC24D | 76.7277 | 60.1188 | 66.3716 | 77.5166 | 109.6865 | 113.149 | 85.23469 | 87.57427 | 105.677 | 0.4058586 | 0.008765 |
| EDEM2 | 57.1377 | 72.1426 | 60.4279 | 48.322 | 115.081 | 88.5982 | 56.82313 | 89.68449 | 83.232 | 0.3969461 | 0.027303 |
| SEC31A | 474.243 | 513.743 | 489.367 | 544.63 | 595.1844 | 663.952 | 669.8573 | 697.4288 | 739.736 | 0.3870369 | 1.61E-06 |
| EDEM3 | 130.6 | 128.982 | 145.621 | 134.899 | 189.7038 | 184.668 | 187.9534 | 168.8179 | 171.14 | 0.3634326 | 0.001729 |
| DNAJB1 | 315.073 | 316.99 | 305.111 | 306.04 | 408.1778 | 428.046 | 438.1937 | 465.3043 | 322.641 | 0.3623105 | 0.000476 |
| RPN1 | 288.953 | 307.153 | 296.196 | 280.872 | 395.5908 | 366.135 | 392.2981 | 418.8793 | 352.567 | 0.3613536 | 4.32E-05 |
| PDIA3 | 1171.32 | 1227.52 | 1122.37 | 1169.8 | 1503.245 | 1389.82 | 1379.054 | 1624.872 | 1645.94 | 0.3448267 | 6.22E-07 |
| SEC24C | 144.477 | 160.681 | 156.518 | 150 | 195.0982 | 182.534 | 209.8085 | 190.9752 | 220.705 | 0.3381415 | 0.002932 |
| DNAJC1 | 186.106 | 230.638 | 195.152 | 266.778 | 275.1154 | 285.009 | 255.7041 | 318.6437 | 293.65 | 0.3281856 | 0.005461 |
| CALR | 2654.45 | 2840.89 | 2616.23 | 2702.01 | 3465.016 | 3337.91 | 3226.898 | 3481.869 | 3281.59 | 0.3049401 | 1.64E-10 |
| HYOU1 | 329.766 | 403.343 | 385.352 | 340.268 | 417.1685 | 466.475 | 441.472 | 494.8474 | 453.568 | 0.2904379 | 0.001601 |
| UBE2J1 | 139.579 | 154.123 | 172.368 | 138.926 | 204.0889 | 175.061 | 210.9012 | 185.6997 | 177.686 | 0.2828623 | 0.020239 |
| SEC61A1 | 328.133 | 406.622 | 408.136 | 351.341 | 437.8471 | 464.34 | 481.9038 | 510.6741 | 414.29 | 0.2745675 | 0.005265 |
| RRBP1 | 1820.24 | 1974.08 | 1764.3 | 1927.85 | 2132.594 | 2070.85 | 2339.583 | 2236.837 | 2514.73 | 0.2567178 | 0.000182 |
| BCAP31 | 360.784 | 385.854 | 388.323 | 425.838 | 466.6174 | 408.832 | 559.4893 | 460.0287 | 449.827 | 0.2345447 | 0.021173 |
| UBXN6 | 250.589 | 259.058 | 262.515 | 220.469 | 312.8764 | 315.964 | 302.6924 | 252.1717 | 304.872 | 0.2289681 | 0.033147 |
| LMAN2 | 635.044 | 734.543 | 672.632 | 677.515 | 766.0077 | 772.832 | 929.9323 | 835.6485 | 720.097 | 0.2216363 | 0.011952 |
| ATF4 | 706.058 | 674.424 | 705.322 | 659.394 | 917.9505 | 736.539 | 813.0078 | 803.9951 | 735.061 | 0.2050834 | 0.014739 |
| HSP90B1 | 3169.51 | 3261.72 | 3241.31 | 3374.49 | 3949.615 | 3822.53 | 3539.425 | 3937.677 | 3626.67 | 0.2038975 | 0.000109 |
| RAD23B | 570.56 | 658.028 | 599.326 | 618.119 | 661.7156 | 703.448 | 716.8456 | 743.8538 | 721.968 | 0.2013212 | 0.008114 |
| PDIA6 | 1515.78 | 1470.18 | 1612.73 | 1612.75 | 1704.637 | 1758.09 | 1727.642 | 1756.761 | 1873.19 | 0.1761688 | 0.002971 |
| CANX | 1783.51 | 1889.92 | 1594.9 | 1752.68 | 1920.414 | 1925.68 | 1922.152 | 2100.727 | 2050.87 | 0.1672331 | 0.012427 |
| CKAP4 | 1475.78 | 1487.67 | 1517.63 | 1653.02 | 1710.93 | 1552.07 | 1807.413 | 1833.784 | 1641.26 | 0.1451537 | 0.048097 |

**Table S5.** *Differentially abundance of Phyla between 6-week-old (A) and 80-week-old (B) Wild-type and PiZ mice.*

**A**

| **Phylum_norm** | **SampleID** | **Abundance** | **RelativeAbundance** | **SampleID** | **Abundance** | **RelativeAbundance** |
| --- | --- | --- | --- | --- | --- | --- |
| p__Fusobacteria | WT 1 | 0.000406504 | 0.00041841 | PiZ 1 | 0.00044484 | 0.000456413 |
| p__Fusobacteria | WT 2 | 0.000536769 | 0.000560224 | PiZ 2 | 0.000363769 | 0.00037092 |
| p__Fusobacteria | WT 3 | 0.000421585 | 0.000434972 | PiZ 3 | 0 | 0 |
|  |  |  |  |  |  |  |
| p__Acidobacteria | WT 1 | 0.000406504 | 0.00041841 | PiZ 1 | 0.00044484 | 0.000456413 |
| p__Acidobacteria | WT 2 | 0.000536769 | 0.000560224 | PiZ 2 | 0.000363769 | 0.00037092 |
| p__Acidobacteria | WT 3 | 0.000421585 | 0.000434972 | PiZ 3 | 0.000305344 | 0.00031056 |
|  |  |  |  |  |  |  |
| p__Synergistetes | WT 1 | 0.000406504 | 0.00041841 | PiZ 1 | 0 | 0 |
| p__Synergistetes | WT 2 | 0.000536769 | 0.000560224 | PiZ 2 | 0.000363769 | 0.00037092 |
| p__Synergistetes | WT 3 | 0.000421585 | 0.000434972 | PiZ 3 | 0 | 0 |
|  |  |  |  |  |  |  |
| p__Spirochaetes | WT 1 | 0.000813008 | 0.00083682 | PiZ 1 | 0.00088968 | 0.000912825 |
| p__Spirochaetes | WT 2 | 0.00161031 | 0.001680676 | PiZ 2 | 0.000727537 | 0.00074184 |
| p__Spirochaetes | WT 3 | 0.00084317 | 0.000869943 | PiZ 3 | 0.000610687 | 0.000621118 |
|  |  |  |  |  |  |  |
| p__Tenericutes | WT 1 | 0.000406504 | 0.00041841 | PiZ 1 | 0.00044484 | 0.000456413 |
| p__Tenericutes | WT 2 | 0.000536769 | 0.000560224 | PiZ 2 | 0.000363769 | 0.00037092 |
| p__Tenericutes | WT 3 | 0.000421585 | 0.000434972 | PiZ 3 | 0.000305344 | 0.00031056 |
|  |  |  |  |  |  |  |
| p__Chloroflexi | WT 1 | 0.000406504 | 0.00041841 | PiZ 1 | 0.00044484 | 0.000456413 |
| p__Chloroflexi | WT 2 | 0.000536769 | 0.000560224 | PiZ 2 | 0.000363769 | 0.00037092 |
| p__Chloroflexi | WT 3 | 0.000421585 | 0.000434972 | PiZ 3 | 0.000305344 | 0.00031056 |
|  |  |  |  |  |  |  |
| p__Deinococcus-Thermus | WT 1 | 0.000813008 | 0.00083682 | PiZ 1 | 0.00044484 | 0.000456413 |
| p__Deinococcus-Thermus | WT 2 | 0.00107354 | 0.00112045 | PiZ 2 | 0.000727537 | 0.00074184 |
| p__Deinococcus-Thermus | WT 3 | 0.00084317 | 0.000869943 | PiZ 3 | 0.000610687 | 0.000621118 |
|  |  |  |  |  |  |  |
| p__Cyanobacteria | WT 1 | 0.00121951 | 0.001255228 | PiZ 1 | 0.00133452 | 0.001369238 |
| p__Cyanobacteria | WT 2 | 0.00214707 | 0.00224089 | PiZ 2 | 0.00109131 | 0.001112764 |
| p__Cyanobacteria | WT 3 | 0.00168634 | 0.001739886 | PiZ 3 | 0.000916031 | 0.000931678 |
|  |  |  |  |  |  |  |
| p__Actinobacteria | WT 1 | 0.0272358 | 0.028033497 | PiZ 1 | 0.019573 | 0.020082201 |
| p__Actinobacteria | WT 2 | 0.03489 | 0.036414587 | PiZ 2 | 0.018916 | 0.019287874 |
| p__Actinobacteria | WT 3 | 0.0265599 | 0.027403257 | PiZ 3 | 0.0189313 | 0.019254666 |
|  |  |  |  |  |  |  |
| p__Firmicutes | WT 1 | 0.096748 | 0.099581607 | PiZ 1 | 0.0520463 | 0.053400311 |
| p__Firmicutes | WT 2 | 0.22598 | 0.235854641 | PiZ 2 | 0.0829393 | 0.084569823 |
| p__Firmicutes | WT 3 | 0.110877 | 0.114397681 | PiZ 3 | 0.0473282 | 0.048136614 |
|  |  |  |  |  |  |  |
| p__Proteobacteria | WT 1 | 0.054065 | 0.055648484 | PiZ 1 | 0.0502669 | 0.051574619 |
| p__Proteobacteria | WT 2 | 0.0821256 | 0.08571424 | PiZ 2 | 0.0378319 | 0.038575646 |
| p__Proteobacteria | WT 3 | 0.056914 | 0.058721192 | PiZ 3 | 0.0351145 | 0.035714292 |
|  |  |  |  |  |  |  |
| p__Planctomycetes | WT 1 | 0.00203252 | 0.00209205 | PiZ 1 | 0.00177936 | 0.001825651 |
| p__Planctomycetes | WT 2 | 0.00268384 | 0.002801116 | PiZ 2 | 0.00145507 | 0.001483676 |
| p__Planctomycetes | WT 3 | 0.00210793 | 0.002174863 | PiZ 3 | 0.00122137 | 0.001242232 |
|  |  |  |  |  |  |  |
| p__Verrucomicrobia | WT 1 | 0.000813008 | 0.00083682 | PiZ 1 | 0.0209075 | 0.021451419 |
| p__Verrucomicrobia | WT 2 | 0.00107354 | 0.00112045 | PiZ 2 | 0.000727537 | 0.00074184 |
| p__Verrucomicrobia | WT 3 | 0.00084317 | 0.000869943 | PiZ 3 | 0.000610687 | 0.000621118 |
|  |  |  |  |  |  |  |
| p__Fibrobacteres | WT 1 | 0 | 0 | PiZ 1 | 0.00044484 | 0.000456413 |
| p__Fibrobacteres | WT 2 | 0 | 0 | PiZ 2 | 0 | 0 |
| p__Fibrobacteres | WT 3 | 0 | 0 | PiZ 3 | 0 | 0 |
|  |  |  |  |  |  |  |
| p__Gemmatimonadetes | WT 1 | 0 | 0 | PiZ 1 | 0 | 0 |
| p__Gemmatimonadetes | WT 2 | 0 | 0 | PiZ 2 | 0 | 0 |
| p__Gemmatimonadetes | WT 3 | 0 | 0 | PiZ 3 | 0 | 0 |
|  |  |  |  |  |  |  |
| p__Chlorobi | WT 1 | 0.000406504 | 0.00041841 | PiZ 1 | 0.00044484 | 0.000456413 |
| p__Chlorobi | WT 2 | 0.000536769 | 0.000560224 | PiZ 2 | 0.000363769 | 0.00037092 |
| p__Chlorobi | WT 3 | 0.000421585 | 0.000434972 | PiZ 3 | 0.000305344 | 0.00031056 |
|  |  |  |  |  |  |  |
| p__Bacteroidetes | WT 1 | 0.785366 | 0.808368216 | PiZ 1 | 0.824733 | 0.846188846 |
| p__Bacteroidetes | WT 2 | 0.603328 | 0.629691604 | PiZ 2 | 0.834121 | 0.850519176 |
| p__Bacteroidetes | WT 3 | 0.76602 | 0.79034346 | PiZ 3 | 0.876641 | 0.891614926 |

**B**

| **Phylum_norm** | **SampleID** | **Abundance** | **RelativeAbundance** | **SampleID** | **Abundance** | **RelativeAbundance** |
| --- | --- | --- | --- | --- | --- | --- |
| p__Fusobacteria | WT_1 | 0 | 0 | PiZ_1 | 0 | 0 |
| p__Fusobacteria | WT_2 | 0 | 0 | PiZ_2 | 0 | 0 |
| p__Fusobacteria | WT_3 | 0 | 0 | PiZ_3 | 0 | 0 |
| p__Fusobacteria | WT_4 | 0 | 0 | PiZ_4 | 0 | 0 |
| p__Fusobacteria | WT_5 | 0 | 0 | PiZ_5 | 0 | 0 |
|  |  |  |  |  |  |  |
| p__Acidobacteria | WT_1 | 0 | 0 | PiZ_1 | 0 | 0 |
| p__Acidobacteria | WT_2 | 0 | 0 | PiZ_2 | 0 | 0 |
| p__Acidobacteria | WT_3 | 0 | 0 | PiZ_3 | 0 | 0 |
| p__Acidobacteria | WT_4 | 0 | 0 | PiZ_4 | 0 | 0 |
| p__Acidobacteria | WT_5 | 0 | 0 | PiZ_5 | 0 | 0 |
|  |  |  |  |  |  |  |
| p__Synergistetes | WT_1 | 0 | 0 | PiZ_1 | 0 | 0 |
| p__Synergistetes | WT_2 | 0 | 0 | PiZ_2 | 0 | 0 |
| p__Synergistetes | WT_3 | 0 | 0 | PiZ_3 | 0 | 0 |
| p__Synergistetes | WT_4 | 0 | 0 | PiZ_4 | 0 | 0 |
| p__Synergistetes | WT_5 | 0 | 0 | PiZ_5 | 0 | 0 |
|  |  |  |  |  |  |  |
| p__Spirochaetes | WT_1 | 0.00045788 | 0.00086881 | PiZ_1 | 0.00041339 | 0.00056754 |
| p__Spirochaetes | WT_2 | 0.00063694 | 0.00082713 | PiZ_2 | 0.00039448 | 0.00078678 |
| p__Spirochaetes | WT_3 | 0.00052274 | 0.0009058 | PiZ_3 | 0.00039698 | 0.00056306 |
| p__Spirochaetes | WT_4 | 0.00110375 | 0.00123456 | PiZ_4 | 0.0009311 | 0.0010929 |
| p__Spirochaetes | WT_5 | 0.00056883 | 0.00095238 | PiZ_5 | 0.00077042 | 0.00089525 |
|  |  |  |  |  |  |  |
| p__Tenericutes | WT_1 | 0 | 0 | PiZ_1 | 0 | 0 |
| p__Tenericutes | WT_2 | 0 | 0 | PiZ_2 | 0 | 0 |
| p__Tenericutes | WT_3 | 0 | 0 | PiZ_3 | 0 | 0 |
| p__Tenericutes | WT_4 | 0 | 0 | PiZ_4 | 0 | 0 |
| p__Tenericutes | WT_5 | 0 | 0 | PiZ_5 | 0 | 0 |
|  |  |  |  |  |  |  |
| p__Chloroflexi | WT_1 | 0 | 0 | PiZ_1 | 0 | 0 |
| p__Chloroflexi | WT_2 | 0 | 0 | PiZ_2 | 0 | 0 |
| p__Chloroflexi | WT_3 | 0 | 0 | PiZ_3 | 0 | 0 |
| p__Chloroflexi | WT_4 | 0 | 0 | PiZ_4 | 0 | 0 |
| p__Chloroflexi | WT_5 | 0 | 0 | PiZ_5 | 0 | 0 |
|  |  |  |  |  |  |  |
| p__Deinococcus-Thermus | WT_1 | 0.00045788 | 0.00086881 | PiZ_1 | 0.00041339 | 0.00056754 |
| p__Deinococcus-Thermus | WT_2 | 0.00063694 | 0.00082713 | PiZ_2 | 0.00039448 | 0.00078678 |
| p__Deinococcus-Thermus | WT_3 | 0.00052274 | 0.0009058 | PiZ_3 | 0.00039698 | 0.00056306 |
| p__Deinococcus-Thermus | WT_4 | 0.00110375 | 0.00123456 | PiZ_4 | 0 | 0 |
| p__Deinococcus-Thermus | WT_5 | 0.00056883 | 0.00095238 | PiZ_5 | 0 | 0 |
|  |  |  |  |  |  |  |
| p__Cyanobacteria | WT_1 | 0.00045788 | 0.00086881 | PiZ_1 | 0.00041339 | 0.00056754 |
| p__Cyanobacteria | WT_2 | 0.00063694 | 0.00082713 | PiZ_2 | 0.00039448 | 0.00078678 |
| p__Cyanobacteria | WT_3 | 0.00052274 | 0.0009058 | PiZ_3 | 0.00039698 | 0.00056306 |
| p__Cyanobacteria | WT_4 | 0.00110375 | 0.00123456 | PiZ_4 | 0.0009311 | 0.0010929 |
| p__Cyanobacteria | WT_5 | 0.00056883 | 0.00095238 | PiZ_5 | 0.00077042 | 0.00089525 |
|  |  |  |  |  |  |  |
| p__Actinobacteria | WT_1 | 0.0732601 | 0.13900965 | PiZ_1 | 0.0640761 | 0.08796834 |
| p__Actinobacteria | WT_2 | 0.106369 | 0.13813011 | PiZ_2 | 0.0796844 | 0.15892984 |
| p__Actinobacteria | WT_3 | 0.0799791 | 0.13858712 | PiZ_3 | 0.0575625 | 0.08164407 |
| p__Actinobacteria | WT_4 | 0.0629139 | 0.07037037 | PiZ_4 | 0.0921788 | 0.10819669 |
| p__Actinobacteria | WT_5 | 0.0733788 | 0.122857 | PiZ_5 | 0.0878274 | 0.10205901 |
|  |  |  |  |  |  |  |
| p__Firmicutes | WT_1 | 0.047619 | 0.09035615 | PiZ_1 | 0.0450599 | 0.06186151 |
| p__Firmicutes | WT_2 | 0.0987261 | 0.12820509 | PiZ_2 | 0.0654832 | 0.13060567 |
| p__Firmicutes | WT_3 | 0.0789336 | 0.13677549 | PiZ_3 | 0.0567686 | 0.08051804 |
| p__Firmicutes | WT_4 | 0.112583 | 0.12592618 | PiZ_4 | 0.0716946 | 0.08415296 |
| p__Firmicutes | WT_5 | 0.0995449 | 0.1666665 | PiZ_5 | 0.087057 | 0.10116378 |
|  |  |  |  |  |  |  |
| p__Proteobacteria | WT_1 | 0.150183 | 0.2849694 | PiZ_1 | 0.117817 | 0.16174776 |
| p__Proteobacteria | WT_2 | 0.291083 | 0.37799854 | PiZ_2 | 0.198028 | 0.39496512 |
| p__Proteobacteria | WT_3 | 0.123366 | 0.21376759 | PiZ_3 | 0.137753 | 0.19538268 |
| p__Proteobacteria | WT_4 | 0.16777 | 0.18765387 | PiZ_4 | 0.20298 | 0.2382518 |
| p__Proteobacteria | WT_5 | 0.24289 | 0.40666699 | PiZ_5 | 0.124037 | 0.14413604 |
|  |  |  |  |  |  |  |
| p__Planctomycetes | WT_1 | 0 | 0 | PiZ_1 | 0 | 0 |
| p__Planctomycetes | WT_2 | 0 | 0 | PiZ_2 | 0 | 0 |
| p__Planctomycetes | WT_3 | 0 | 0 | PiZ_3 | 0 | 0 |
| p__Planctomycetes | WT_4 | 0 | 0 | PiZ_4 | 0 | 0 |
| p__Planctomycetes | WT_5 | 0 | 0 | PiZ_5 | 0 | 0 |
|  |  |  |  |  |  |  |
| p__Verrucomicrobia | WT_1 | 0.00045788 | 0.00086881 | PiZ_1 | 0.140554 | 0.19296277 |
| p__Verrucomicrobia | WT_2 | 0.00063694 | 0.00082713 | PiZ_2 | 0.00039448 | 0.00078678 |
| p__Verrucomicrobia | WT_3 | 0.00052274 | 0.0009058 | PiZ_3 | 0.143311 | 0.2032659 |
| p__Verrucomicrobia | WT_4 | 0.00110375 | 0.00123456 | PiZ_4 | 0.0009311 | 0.0010929 |
| p__Verrucomicrobia | WT_5 | 0.00056883 | 0.00095238 | PiZ_5 | 0.00077042 | 0.00089525 |
|  |  |  |  |  |  |  |
| p__Fibrobacteres | WT_1 | 0 | 0 | PiZ_1 | 0 | 0 |
| p__Fibrobacteres | WT_2 | 0 | 0 | PiZ_2 | 0 | 0 |
| p__Fibrobacteres | WT_3 | 0 | 0 | PiZ_3 | 0 | 0 |
| p__Fibrobacteres | WT_4 | 0 | 0 | PiZ_4 | 0 | 0 |
| p__Fibrobacteres | WT_5 | 0 | 0 | PiZ_5 | 0 | 0 |
|  |  |  |  |  |  |  |
| p__Gemmatimonadetes | WT_1 | 0 | 0 | PiZ_1 | 0 | 0 |
| p__Gemmatimonadetes | WT_2 | 0 | 0 | PiZ_2 | 0 | 0 |
| p__Gemmatimonadetes | WT_3 | 0 | 0 | PiZ_3 | 0 | 0 |
| p__Gemmatimonadetes | WT_4 | 0 | 0 | PiZ_4 | 0 | 0 |
| p__Gemmatimonadetes | WT_5 | 0 | 0 | PiZ_5 | 0 | 0 |
|  |  |  |  |  |  |  |
| p__Chlorobi | WT_1 | 0.00045788 | 0.00086881 | PiZ_1 | 0.00041339 | 0.00056754 |
| p__Chlorobi | WT_2 | 0.00063694 | 0.00082713 | PiZ_2 | 0.00039448 | 0.00078678 |
| p__Chlorobi | WT_3 | 0.00052274 | 0.0009058 | PiZ_3 | 0.00039698 | 0.00056306 |
| p__Chlorobi | WT_4 | 0.00110375 | 0.00123456 | PiZ_4 | 0.0009311 | 0.0010929 |
| p__Chlorobi | WT_5 | 0 | 0 | PiZ_5 | 0.00077042 | 0.00089525 |
|  |  |  |  |  |  |  |
| p__Bacteroidetes | WT_1 | 0.253663 | 0.48132075 | PiZ_1 | 0.359239 | 0.49318947 |
| p__Bacteroidetes | WT_2 | 0.270701 | 0.35153061 | PiZ_2 | 0.156213 | 0.31156547 |
| p__Bacteroidetes | WT_3 | 0.292211 | 0.50634081 | PiZ_3 | 0.308059 | 0.43693707 |
| p__Bacteroidetes | WT_4 | 0.545254 | 0.60987675 | PiZ_4 | 0.481378 | 0.56502697 |
| p__Bacteroidetes | WT_5 | 0.179181 | 0.29999999 | PiZ_5 | 0.558552 | 0.64906015 |

**Table S6.** *List of reagents used for the study.*

| **Antigen** | **Source** | **Code** |
| --- | --- | --- |
| Dextran Sulfate Sodium Salt, Colitis Grade (36,000 to 50,000) | MP BIOCHEMICALS | 216011080 |
| Lysozyme human recombinant | MERK | L1667 |
| Stool DNA Isolation Kit (Magnetic Bead System) | NORGEN BIOTEK CORP. | 55700 |
| Thapsigargin >=98% | SIGMA | T9033 |
| 4-phenylbutyric acid (99%) | MEDCHEM | HY-A0281 |
| Carbamylcholine chloride crystalline | MERK | C4382 |
| Chloroquine diphosphate salt | MERK | C6628 |
| Dynabeads™ Protein G | THERMOFISHER | 10003D |
| Thapsigargin >=98% | SIGMA | T9033 |
| 4-phenylbutyric acid (99%) | MEDCHEM | HY-A0281 |
| 4-phenylbutyric acid sodium | MEDCHEM | HY-15654 |
| DynaMag magnet | THERMOFISHER | 12321D |
| PAS-periodic acid Schiff | BIO-OPTICA | 04-130802 |
| Nuclear Fast Red | VECTORLABS | H-3403 |
| Direct Red 80 | MERK | 365548 |
| Hematoxilin | MERK | H3136 |
| Eosin Y solution Alcoholic | MERK | HT110116 |
| Alcian Blue | MERK | B8438 |
| High-capacity cDNA reverse trascription kit | THERMOFISHER | 4368814 |
| LyghtCycler 480 SYBR Green I Master | ROCHE | RO04887352001 |
| Antigen Unmasking Solution Citric Acid based | VECTORLABS | H-3300-250 |
| Recombinant Human/mouse/rat Activin A protein | BIO-TECHNE | 338-AC-010 |
| Recombinant Human FGF-4 Protein | BIO-TECHNE | 235-F4 |
| Geltrex™ Reduced-Growth Factor Basement-Membrane Matrix, LDEV-free, stem-cell qualified | THERMOFISHER | A1413302 |
| mTESR 1 media | STEMCELL TECHNOLOGIES | 85850 |
| Gibco™ B-27™ Supplement (50X), minus vitamin A | THERMOFISHER | 12587010 |
| Gibco™ N-2 Supplement (100X) | THERMOFISHER | 17502048 |
| Recombinant Human Noggin | PEPROTECH | 120-10C |
| Recombinant Human EGF | PEPROTECH | 315-09 |
| Advanced DMEM/F12 | THERMOFISHER | 12634010 |
| GlutaMAX™ Supplement | THERMOFISHER | 04-130802 |
| HEPES (1M) | THERMOFISHER | 15630080 |
| Penicillin-Streptomycin (10,000 U/mL) | THERMOFISHER | 15140122 |
| VECTASHIELD® Antifade Mounting Medium | VECTORLABS | H-1000-10 |
| Falcon® 70 µm Cell Strainer | EUROCLONE | FLC352350 |
| QIAzol Lysis Reagent | QIAGEN | 79306 |
| Rneasy Mini Kit | QIAGEN | 74106 |
| Donkey serum | EUROCLONE | ECS0217L |
| Optimal Cutting Temperature (OCT) compound | KALTEK | 0782 |
| Phosphatase Inhibitor cocktail 3 | MERK | P0044 |
| Protease inhibitor cocktail, 1ml | MERK | P8340 |

**Table S7.** *Primary antibodies used for immunoblots and stainings*

| **Antigen** | **Source** | **Code** |
| --- | --- | --- |
| Polyclonal Anti Human Alpha-1 Antitrypsin rabbit | DAKO | A0012 |
| Anti-Human Alpha-1 Antitrypsin clone 2C1 mouse | HYCULT | HM2289 |
| Anti-lysozyme rabbit | INVITROGEN | MA532154 |
| Anti-Olfm4 (211-510) rabbit | ST JOHN’S LABORATORY | STJ117582 |
| Anti-Chromogranin A rabbit | ABCAM | Ab283265 |
| Anti-MUC2 rabbit | ANTIBODIES.com | A91628 |
| Anti-LC3B rabbit | NOVUS | NB100-2220 |
| Anti-LC3AB rabbit | CELL SIGNALING | 4108 |
| Anti-Lamp1 rabbit | ABCAM | Ab24170 |
| Anti-Vinculin (E1E9V) rabbit | CELL SIGNALING | 13901 |
| Anti-beta Actin | INVITROGEN | AM4302 |
| Anti-phospho-PERK (Thr980) rabbit | CELL SIGNALING | 3179 |
| Anti-PERK rabbit | CELL SIGNALING | 3192 |
| Anti-phospho-IRE1 alpha (Ser724) rabbit | INVITROGEN | PA1-16927 |
| Anti-IRE1 alpha rabbit | CELL SIGNALING | 3294 |
| Anti-ATF6 alpha mouse | SANTA CRUZ | Sc-166659 |
| Anti-phospho-eIF2 alpha rabbit | CELL SIGNALING | 9721 |
| Anti-eIF2 alpha rabbit | CELL SIGNALING | 9722 |
| Anti-BiP rabbit | CELL SIGNALING | 3177 |
| Anti-CHOP mouse | CELL SIGNALING | 2895 |
| Anti-XBP1-S rabbit | CELL SIGNALING | 40435 |
| Anti-P62/SQSTM1mouse | ABNOVA | H00008878-M01 |

**Table S8**. *Primers for real time PCR*

| **Gene** | **Species** |  | **Sequence (5’🡪 3’)** |
| --- | --- | --- | --- |
| *S16* | mouse | forward | AGGAGCGATTTGCTGGTGTGG |
|  |  | reverse | GCTACCAGGGCCTTTGAGATG |
| *TNF alpha* | mouse | forward | CGTCAGCCGATTTGCTATCT |
|  |  | reverse | CGGACTCCGCAAAGTCTAAG |
| *IL1-Beta* | mouse | forward | GCCCATCCTCTGTGACTCAT |
|  |  | reverse | AGGCCACAGGTATTTTGTCG |
| *SERPINA1* | Human | forward | TGCCCAGGTATTTCATCAGC |
|  |  | reverse | CCGTGAAGGTGCCTATGATG |
| *Lyz1* | mouse | forward | GTGCCTGTCCTGATCTTTCT |
|  |  | reverse | GATTTGCTCCTGTGGTTATTGG |

**Table S9**. R*eal time PCR primers for microbiota analysis*

| **Gene** | **Species** |  | **Sequence (5’🡪 3’)** |
| --- | --- | --- | --- |
| *Universal bacteria S16 rRNA* | Bacteria | forward | ACTCCTACGGGAGGCAGCAGT |
|  |  | reverse | GTATTACCGCGGCTGCTGGCAC |
| *Firmicutes 16S rRNA* | Bacteria | forward 1 | GGAGCATGTGGTTTAATTCGAAGCA |
|  |  | forward 2 | GGAGTATGTGGTTTAATTCGAAGCA |
|  |  | reverse | AGCTGACGACAACCATGCAC |
| *Bacteroidetes 16S rRNA* | Bacteria | forward | CGATGGATAGGGGTTCTGAGAGGA |
|  |  | reverse | GCTGGCACGGAGTTAGCCGA |
